# Evaluation of Pd→B Interactions in Diphosphinoborane Complexes and Impact on Inner‐Sphere Reductive Elimination

**DOI:** 10.1002/chem.202001189

**Published:** 2020-09-18

**Authors:** Florian Ritter, Lukas John, Tobias Schindler, Julian P. Schroers, Simon Teeuwen, Michael E. Tauchert

**Affiliations:** ^1^ Institute of Inorganic Chemistry RWTH Aachen University Landoltweg 1A 52074 Aachen Germany

**Keywords:** boranes, donor–acceptor systems, palladium, phosphine ligands, reductive elimination

## Abstract

The dative Pd→B interaction in a series of ^R^DPB^R’^ Pd^0^ and Pd^II^ complexes (^R^DPB^R’^=(*o*‐PR_2_C_6_H_4_)_2_BR’, diphosphinoborane) was analyzed using XRD, ^11^B NMR spectroscopy and NBO/NLMO calculations. The borane acceptor discriminates between the oxidation state Pd^II^ and Pd^0^, stabilizing the latter. Reaction of lithium amides with [(^R^DPB^R’^)Pd^II^(4‐NO_2_C_6_H_4_)I] chemoselectively yields the C−N coupling product. DFT modelling indicates no significant impact of Pd^II^→B coordination on the inner‐sphere reductive elimination rate.

## Graphical abstract

**Ligand effects**: The borane acceptor in diphosphinoborane ligands discriminates between the oxidation state Pd^II^ and Pd^0^, stabilizing the latter. The impact of the Pd→B interaction on inner‐sphere C−N bond reductive elimination of *N*,*N*‐dimethyl‐4‐nitroaniline was investigated (see scheme).

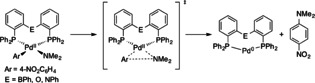

## Introduction

**Figure 1:**
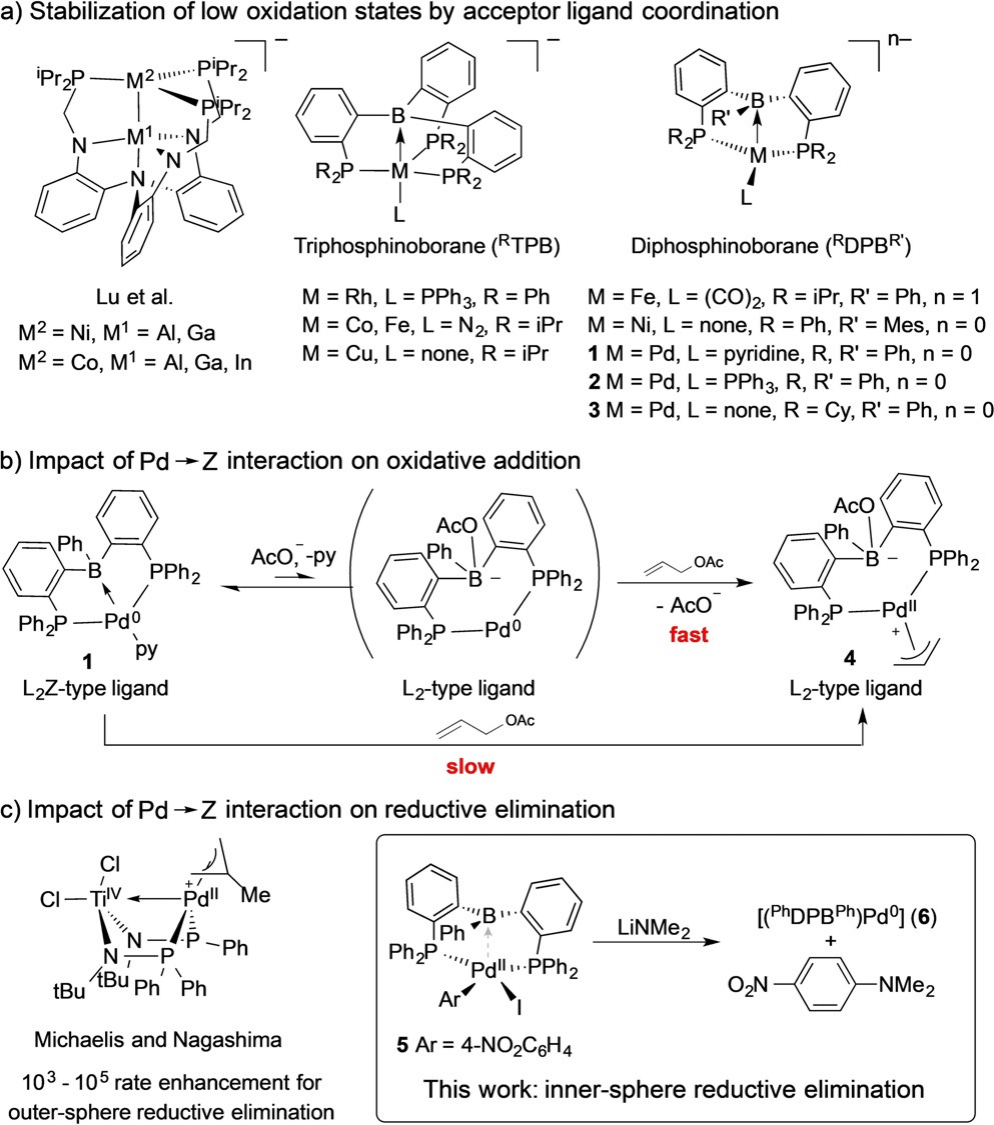
M→Z interaction: stabilization of low oxidation states and impact on oxidative addition and reductive elimination.

Z‐type acceptor ligands have attracted considerable attention over the past decade.^1^ Their coordination to transition metals grants access to complexes with unusual coordination geometries^2^ and electronic properties by formation of dative M→Z bonds. Group 13 acceptor ligands, with a special focus on boranes, have been particularly well studied. M→Z bonds can stabilize low oxidation states at the coordinated transition metal.^3^ Thus, facile access to complexes featuring transition metals with formally negative oxidations states is realized (Figure 1 a).^4^ This stabilization of low oxidation states appears to inhibit oxidative addition reactions.3b, 3e, 5 However, we demonstrated that this obstacle can be overcome for complex **1** by addition of catalytic amounts of acetate, which competes with Pd^0^ for the free coordination site at the borane, thus reversibly breaking the Pd^0^→B interaction (Figure 1 b).3b This concept allowed for the application of **1** in catalytic allylic amination, and most recently of **2** in the catalytic hydro‐/deutero‐dechlorination of aryl chlorides.3e Alternatively, bifunctional substrate activation across the M→Z interaction has been described.3a, 6 The aptitude of hydride,^7^ halide^8^ and carbon group^9^ migration between the Z‐type ligand and the coordinated transition metal has initiated further applications. Catalytic processes have concentrated on transformations in which the catalyst is not required to change its oxidation state quickly, but rather profits from an electronic fine‐tuning by electron‐withdrawing Z‐ligand coordination.^10^ Successful applications include CO_2_ hydrogenation^11^ and hydrosilylation,3d, 12 enyne cycloisomerization^13^ and alkyne hydroamination.^14^ Michaelis used the heterobimetallic Ti^IV^/Pd^II^ complex (Figure 1 c), developed by Nagashima,^15^ for allylic amination of allyl chlorides with hindered secondary amines.5b, 16

Combined experimental and computational investigations indicated a rate enhancement of 10^3−^–10^5^ of the outer‐sphere reductive C−N bond elimination, due to the electron‐withdrawing Pd^II^→Ti^IV^ interaction.5b, 17 This result agrees with previous investigations performed with Pd η^3^‐allyl and Ni η^3^‐allyl complexes, which showed favored reductive outer‐sphere reductive elimination in the presence of less electron‐donating spectator ligands.^18^

We speculated that the electron‐withdrawing properties of the borane functionality in diphosphinoborane (DPB) ligands enhances the rate of inner‐sphere reductive elimination from Pd complexes due to 1) overall reduced electron density at the Pd^II^ center and 2) increasing of the Pd→B interaction strength during reductive elimination. We determine how the oxidation state of Pd and co‐ligands affect the strength of the Pd→B interaction in DPB complexes. NBO/NLMO calculations and solid‐state structures are used to assess the strength of Pd→B interactions. The value of the ^11^B NMR chemical shift as a probe is discussed. The reductive elimination of *N*,*N*‐dimethyl‐4‐nitroaniline from [(^Ph^DPB^Ph^)Pd^II^(4‐NO_2_‐C_6_H_4_)NMe_2_] (**5**) was studied and modelled with DFT calculations to investigate the assumed influence of the borane acceptor.

## Results and Discussion

### Syntheses and reactivity of [(DPB)Pd] complexes

**Scheme 1:**
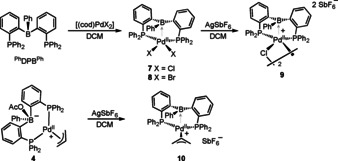
Synthesis of [(^Ph^DPB^Ph^)Pd^II^] complexes.

A series of [(^Ph^DPB^Ph^)Pd^II^] complexes was synthesized to examine a possible correlation between the nature of ligands at Pd and the strength of the Pd^II^→B interaction (Scheme 1).

**Figure 2:**
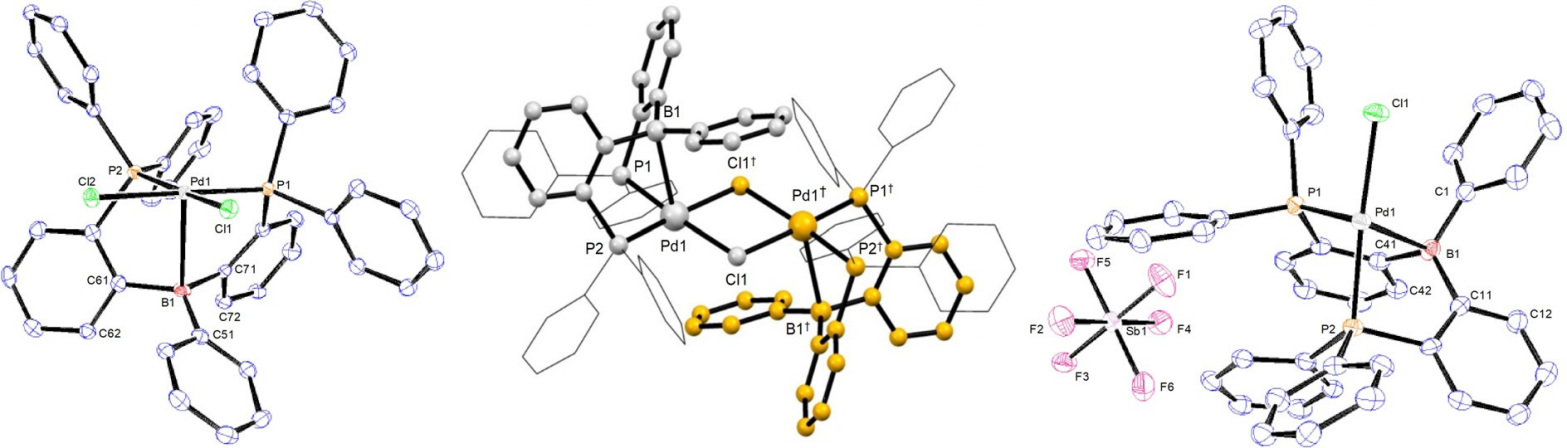
Left: thermal ellipsoid plot of the solid‐state structure of **7** at the 50 % probability level. Hydrogen atoms are omitted for clarity. Selected bond lengths (Å) and angles (°): Pd1−Cl1=2.3355(7), Pd1−Cl2=2.3628(7), Pd1−P1=2.2558(8), Pd1−P2=2.2932(8), Pd1−B1=2.762(3), Pd1−C51=3.405(3), P1‐Pd1‐P2=95.49(3), C51‐B1‐C61=118.3(3), C51‐B1‐C71=118.2(3), C71‐B1‐C61=118.8(3).^22^ Middle: Ball and stick display of [(^Ph^DPB^Ph^)PdCl]‐dimer (**9**) generated by symmetry. Right: thermal ellipsoid plot of the asymmetric unit of **9** at the 50 % probability level. Hydrogen atoms and crystal CH_2_Cl_2_ are omitted for clarity. Selected bond lengths (Å) and angles (°): Pd1−Cl1=2.3781(11), Pd1−Cl1^†^=2.3928(13), Pd1−P1=2.2638(13), Pd1−P2=2.3084(11), Pd1−B1=2.721(5), Pd1−C1=3.338(4), P1‐Pd1‐P2=95.38(5), C11‐B1‐C41=117.5(4), C1‐B1‐C11=119.4(4), C1‐B1‐C41=118.9(4).^23^.

Complex [(^Ph^DPB^Ph^)Pd^II^Cl_2_] (**7**) was produced by reaction of ^Ph^DPB^Ph^ ligand with [(cod)PdCl_2_] in DCM and was isolated in 74 % yield (Scheme 1). Single crystals were grown from CH_2_Cl_2_/benzene and analyzed by X‐ray diffraction (Figure 2). A typical square‐pyramidal coordination around the palladium was observed around the Pd^II^ center. The chloride ligands are located in *cis*‐configuration at the basal position, and the borane adopts the apical position. The Pd,B distance of 2.762(3) Å is shorter than the sum of the van der Waals radii (3.28 Å),^19^ but elongated compared to the sum of the covalent radii (2.23 Å).^20^ A long Pd,C51 distance of 3.405(3) Å seems to rule out a η^2^‐(B,C) type coordination to the Pd^II^ center. A slightly increased pyramidalization at the boron atom is observed (*Σ*B_*α*_=355.4°) compared to complex [(^*i*Pr^DPB^Ph^)PdCl_2_] (*Σ*B_*α*_=359.9°).^21^

**Figure 3:**
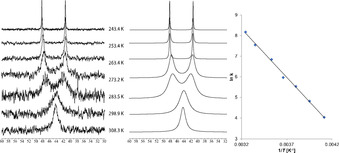
^31^P VT NMR analysis of **7** in CD_2_Cl_2_. Left: recorded ^31^P NMR spectra. Middle: simulated ^31^P NMR spectra. Right: Arrhenius plot.

The ligand backbone is twisted (dihedral angle C62‐C61‐C71‐C72: 35.6(3)°) to allow for a P‐Pd‐P angle of 95.49(3)°. This twist renders the two phosphine groups diastereotopic. The ^31^P NMR spectrum of **7** in CD_2_Cl_2_ displays two broad resonances of equal integral at *δ=*39.0 and 48.2 ppm. A series of ^31^P VT NMR spectra was recorded (Figure 3), covering a temperature range from −29.8 to 35.1 °C. The two singlet resonances coalesced into a single resonance (*δ*=48.2 ppm) at elevated temperatures. The rate constants of the dynamic process were determined by line‐shape analysis using Bruker's TopSpin software. An Arrhenius plot analysis gave an activation energy of *E_a_*=9.3±0.5 kcal mol^−1^ with a pre‐exponential factor of *A*=(14±7) *x* 10^9^.

**Scheme 2:**
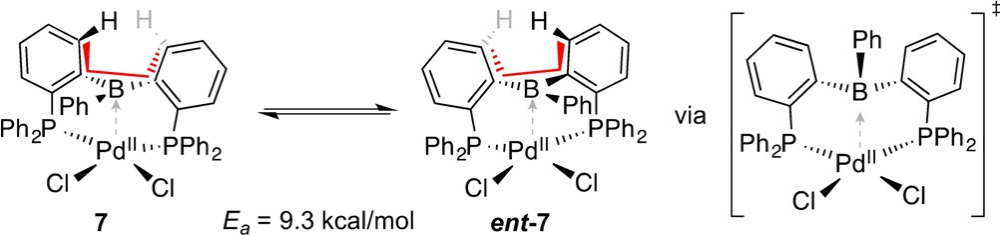
Proposed interconversion between **7** and ***ent*** **‐7** by twisting of the DPB ligand.

We suggest that the observed dynamic process in the ^31^P NMR spectrum of **7** is caused by an interconversion of **7** with its enantiomer ***ent*** **‐7** (Scheme 2).

In order to accommodate for the small P‐Pd‐P angle of 95.49(3)°, the *σ*‐symmetric ^Ph^DPB^Ph^ ligand is twisted. As a result, its B−Ph group points towards one of the two phosphine groups, rendering them chemically inequivalent. This assumption is in line with the observed two ^31^P NMR resonances at low temperatures. Twisting of the C62‐C61‐C71‐C72 dihedral angle converts **7** into its enantiomer ***ent*** **‐7**, presumably via a σ‐symmetric transition in which the B−Ph group is orientated between the two chloro ligands.

Complex **8** was synthesized in the same fashion as **7** from [(cod)PdBr_2_] and was isolated in 67 % yield. The ^31^P NMR spectrum displays two broad resonances of equal intensity at *δ*=45.2 and 38.1 ppm (CD_2_Cl_2_), suggesting a similar dynamic process as in **7**. Due to the poor solubility of both **7** and **8**, no ^11^B NMR spectra could be obtained.

Cationic complex [(^Ph^DPB^Ph^)Pd^II^Cl]SbF_6_ (**9**) was produced in 51 % isolated yield by halide abstraction from **7** with AgSbF_6_ (Scheme 1). Single crystals were grown from CH_2_Cl_2_/hexane and analyzed by X‐ray diffraction (Figure 2). In the solid state a chloro‐bridged dimer [(^Ph^DPB^Ph^)Pd^II^(μ‐Cl)]_2_(SbF_6_)_2_ is observed with an inversion center between the two Pd^II^ centers. Within the dimer, the Pd^II^ center is coordinated in a square‐pyramidal fashion with the borane located in the apical position. The Pd, B distance in complex **9** is 2.721(5) Å, which is slightly shorter than in [(^Ph^DPB^Ph^)Pd^II^Cl_2_] **7** (2.762(3) Å). However, pyramidalization of the borane is almost identical (*Σ*B_*α*_=355.8°). The absence of a relevant η^2^(B,C)→Pd^II^ interaction is suggested by the long Pd1,C1 distance of 3.338(4) Å. The Pd,B distance and lack of significant pyramidalization at the borane suggest a weak Pd^II^→B interaction, which is in line with a broad resonance in the ^11^B NMR spectrum at *δ*=65 ppm (*ω*_1/2_=1900±500 Hz).

The ligand backbone is twisted similarly to that in **7** (dihedral angle C42‐C41‐C11‐C12 of 33.5(5)° (**9**) vs. 35.6(3)° in **7**), resulting in an almost parallel orientation of the B−Ph with the Pd1−Cl1 bond (dihedral angle C1‐B1‐Pd1‐Cl1 of 10.6(3)°). The ^31^P NMR spectrum of **9** displayed only a singlet resonance at *δ*=49.9 ppm which suggests a quick interconversion between the two diastereotopic phosphine donors in solution.

**Figure 4:**
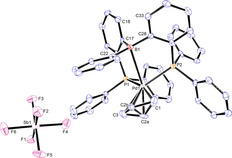
Thermal ellipsoid plot of the solid‐state structure of **10** at the 50 % probability level. Hydrogen atoms and one molecule of CH_2_Cl_2_ are omitted for clarity. Selected bond lengths (Å) and angles (°): Pd1−B1=2.676(5), Pd1−C22=3.066(6), Pd1−P1=2.304(1), Pd1−P2=2.340(1), Pd1−C1=2.191(5), Pd1−C2a=2.186(12), Pd1−C2b=2.192(7), Pd1−C3=2.201(4), P1‐Pd1‐P2=102.86(5), P1‐Pd1‐B1=82.1(1), P2‐Pd1‐B1=75.1(1).^24^.

Cationic allyl complex [(^Ph^DPB^Ph^)Pd^II^(η^3^‐C_3_H_5_)]SbF_6_ (**10**) was synthesized by reaction of AgSbF_6_ with zwitterionic allyl complex [{(*o*‐PPh_2_C_6_H_4_)_2_B(OAc)Ph}Pd^II^(C_3_H_5_)] (**4**) (Scheme 1) and was isolated in 38 % yield by crystallization from CH_2_Cl_2_/hexane. Figure 4 depicts its solid‐state structure. The Pd^II^ center in complex **10** is located in a trigonal‐pyramidal environment in which the borane occupies the pseudo‐apical position and the C_3_H_5_‐ligand and the two phosphines are located in the trigonal‐planar positions. A weak Pd^II^→B interaction is indicated by a Pd,B distance of 2.676(5) Å, which is in line with a minor pyramidalization at the borane center (*Σ*B_*α*_=354.7°) and a broad ^11^B NMR resonance at *δ*=62 ppm (*ω*_1/2_=1200±100 Hz). A large Pd,C22 distance of 3.066(6) Å eliminates the possibility of a strong η^2^(B,C)→Pd^II^ interaction. The η^3^‐coordinated C_3_H_5_‐ligand is disordered. Using the borane as a reference point, a 39:61 mixture of the *exo*‐ and *endo*‐isomers is observed. A wider P‐Pd‐P angle of 102.86(5)° is realized by a decrease in the twisting of the ligand backbone (dihedral angle C18‐C17‐C28‐C33 of 24.04°). The observed disorder of the C_3_H_5_‐ligand is in good agreement with the observed NMR spectra. In the ^31^P NMR spectrum (CD_2_Cl_2_), two singlet resonances are observed in a 40:60 ratio (*δ*=28.1 and 26.9 ppm) and two sets of C_3_H_5_‐units are detected in the ^1^H NMR spectrum. DFT calculations (BP86/def‐SV(P)) based on the solid‐state structures of **10‐*endo*** and **10‐*exo*** indicate a small Gibbs free energy preference of Δ*G*=0.74 kcal mol^−1^ for **10‐*endo***, predicting a 29:71 ratio at 298 K.

**Scheme 3:**
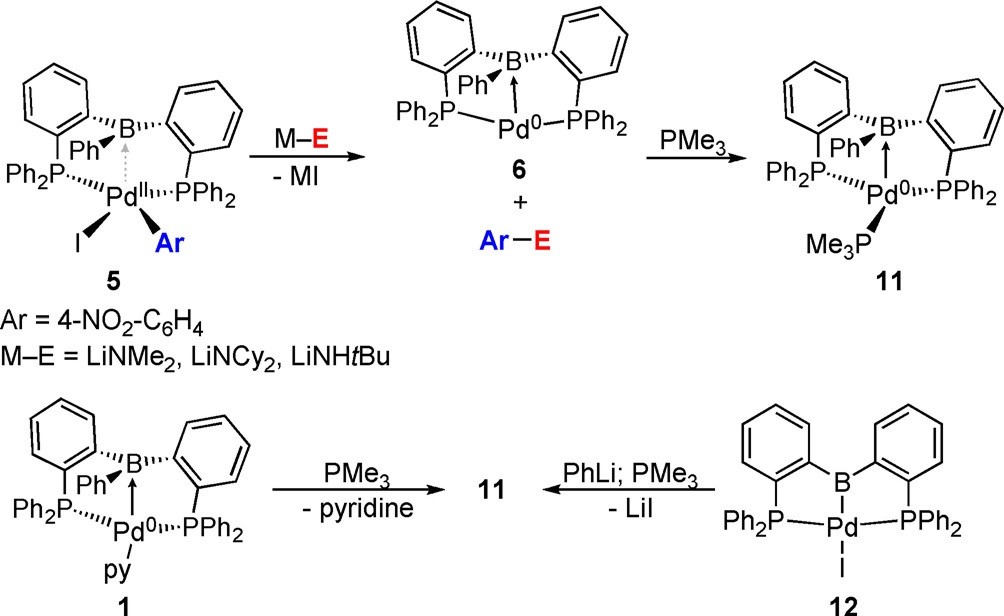
Reductive elimination from **5** and independent synthesis of **11**.

To explore the potential influence of the Pd^II^→B interaction on reductive elimination proceeding via an inner‐sphere mechanism, complex [(^Ph^DPB^Ph^)Pd^II^(4‐NO_2_‐C_6_H_4_)I] (**5**) was reacted with lithium amides. Complex **5** was reacted with LiNMe_2_ (1.1 equiv) at room temperature in [D_8_]THF (Scheme 3).^25^

A conversion of 84 % was observed ^31^P NMR spectroscopically after 1 h. Two complexes were formed with singlet resonances at *δ*=31.1 (70 %) and 38.3 ppm (14 %). After a total of 4.5 h, all resonances in the ^31^P NMR spectrum disappeared in favor of the singlet at *δ*=31.1 ppm. ^11^B NMR spectroscopy suggested formation of a zero‐valent palladium complex by a broad resonance at *δ*=19 ppm (*ω*_1/2_=400±100 Hz). The concurrent formation of the expected reductive elimination product *N*,*N*‐dimethyl‐4‐nitroaniline was confirmed by GC/MS analysis, using an independently prepared sample as a reference. The absence of an intermediate complex *cis*‐[(^Ph^DPB^Ph^)Pd^II^(4‐NO_2_‐C_6_H_4_)NMe_2_] suggests that transmetalation is rate‐limiting in this transformation. The intermediate occurrence of the ^31^P NMR resonance at *δ*=38.3 ppm is possibly due to a reversible reaction of LiNMe_2_ with complex **6**. In a control experiment complex [(^Ph^DPB^Ph^)Pd^0^(pyridine)] (**1**) was reacted with LiNCy_2_ and LiNMe_2_ in [D_8_]THF. In both cases ca. 7 % of a new complex at *δ*=38.5 (s) and 37.7 ppm (s) were observed.

Complex **6** decomposed within hours with simultaneous precipitation of palladium black. Addition of PMe_3_ as a stabilizing co‐ligand led to the formation of complex [(^Ph^DPB^Ph^)Pd^0^(PMe_3_)] **11**. The ^31^P NMR spectrum of **11** showed a doublet at *δ*=35.3 and a triplet at −40.1 ppm (*J*=15.1 Hz) in a 2:1 ratio, which is consistent with the expected κ^3^P‐coordination. The broad resonance in the ^11^B NMR spectrum at *δ*=25 ppm (*ω*_1/2_=400±100 Hz) suggested a strong Pd^0^→B interaction. Complex **11** could also be synthesized independently by reaction of PBP pincer **12** with PhLi and PMe_3_, or reaction of **1** with PMe_3_, thus confirming unambiguously the identity of **11** (Scheme 3).

Complex **5** reacted in a similar fashion with LiNCy_2_ (26 % **6** after 3 h) and LiNH*t*Bu (14 % **6** after 5.5 h). However, the reaction proceeded slower with these sterically more demanding substrates. The reaction of complex **5** with LiNH*t*Bu was monitored for 96 h by ^31^P NMR spectroscopy (46 % conversion towards **6**) without any side products being observed (cf. Table S1). This is in line with the assumption of a rate‐determining transmetalation followed by a quick reductive elimination.

### Analyses of Pd→B interactions

The solid‐state structures of Pd^0/II^ DPB complexes were analyzed to identify factors which affect the strength of Pd→B interactions. In addition to the new Pd complexes presented in this work (**6**–**10**), the structurally characterized DPB complexes *cis*‐[(^Ph^DPB^Ph^)Pd^II^(4‐NO_2_‐C_6_H_4_)I] (**5**),9d [(^Ph^DPB^Ph^)Pd^0^(pyridine)] (**1**),3b [(^Ph^DPB^Me^)Pd^0^(PMe_3_)] (**13**)9d and [(^Cy^DPB^Ph^)Pd^0^] (**3**)3c (Figure 4) were included to cover a broad range of B‐/P‐substituents and co‐ligands at the Pd^0/II^ center. The shorter Pd,B distances and higher degree of borane pyramidalization (Table 1) confirm a significantly stronger Pd,B interaction in Pd^0^ complexes, than in Pd^II^ complexes. Surprisingly, within a given oxidation state only a very moderate variation of the Pd→B bond strength is observed, regardless of substituents at the borane and phosphines, or the number and nature of co‐ligands (Pd^0^: *Σ*B_*α*_=338–346°, d(Pd^0^,B)=2.194(3) −2.243(2) Å vs. Pd^II^: *Σ*B_*α*_=354–356°, d(Pd^II^,B)=2.676(5) −2.762(2) Å). Remarkably, even the generation of cationic Pd^II^ complexes (**9** and **10**) has no significant impact on the strength of Pd^II^→B interactions. The oxidation state at Pd is unambiguously the dominant factor for the strength of the Pd,B bond.

**Table 1:** Experimental and computational analysis of the Pd→B interactions.^[a]^

	**7**	**8**	**9^[e]^**	**10‐*endo***	**5**	**1**	**13**	**3**	**6**
*d*(Pd,B) [Å] (XRD/DFT)	2.762(3) 2.740	– −2.654	2.721(5) 2.554	2.676(5) 2.731	2.7402(4) 2.781	2.194(3) 2.193	2.278(3) 2.360	2.243(2) 2.264	– −2.253
(Pd,C_*ipso*_) [Å] (XRD/DFT)	3.405(3) 3.256	– −3.292	3.338(4) 3.112	3.066(6) 3.259	3.346(4) 3.440	2.463(3) 2.865	2.815(2) 2.685	3.079(2) 3.054	– −2.768
*Σ*B_*α*_ [°] (xrd/dft)	355/355	–/352	356/355	355/355	354/351	346/346	338/341	341/343	–/349
^11^B NMR (*δ*, *ω*_1/2_)	–	–	65 ppm 1900 Hz	67 ppm 1400 Hz	63 ppm 3000 Hz	20 ppm 400 Hz	25 ppm 500 Hz	22 ppm 800 Hz	19 ppm 400 Hz
*E*_2_(Pd,B)^[b]^ [kcal/mol]	11.46	10.42	11.41	8.04	8.72	23.46	19.53	46.83	42.12
NLMO %B^[c]^/Pd^[c]^	6.6/91.9	6.3/92.2	5.4/92.9	3.7/93.9	4.7/93.4	16.0/78.7	15.0/81.5	15.5/81.7	14.3/83.0
occ. B^[d]^	0.391	0.387	0.400	0.360	0.353	0.618	0.621	0.498	0.519
occ. Pd^[d]^	1.859	1.865	1.870	1.887	1.879	1.666	1.702	1.686	1.704
B‐hybrid % (s/p)	7.6/92.4	7.2/2.7	7.2/92.7	6.7/93.3	6.4/93.6	11.6/88.4	13.9/86.1	12.8/87.2	10.7/89.3
WBI (Pd,B)	0.2164	0.2063	0.2119	0.1738	0.1801	0.4207	0.3634	0.5032	0.4604
WBI (Pd,C_*ipso*_)	0.0079	0.0079	0.0208	0.0093	0.0062	0.0697	0.0171	0.0103	0.0325

[a] Structure optimization: Turbomole 7.0.1, BP86/def‐SV(P); NBO analysis: Gaussian 09/NBO 6.0, BP86/6‐31G(d), MWB10 (P,Cl), MWB28 (Pd, Br), MWB46 (I). [b] NBO stabilizing energy E_2_ associated with the Pd→B interaction. [c] Contribution of the donor/acceptor NBO to the NLMO. [d] Occupancy of the donor/acceptor NBO. [e] Calculated structure parameters of **9** are based on the monomer.

**Figure 5:**
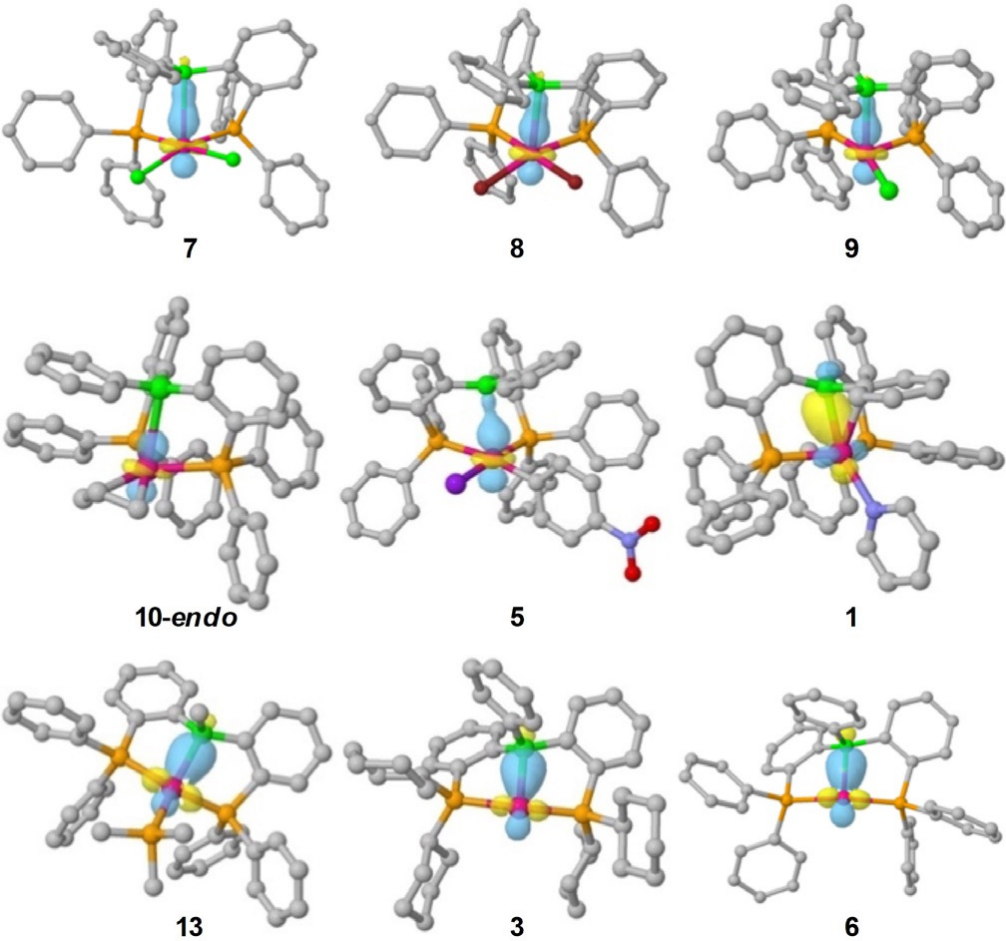
Graphical representation of the NLMOs associated with the Pd→B interactions in [(^Ph^DPB^Ph^)Pd(0/II)] complexes.

The Pd→B interactions were further analyzed using QM calculations. Complexes **1**, **3**, **5**–**11** and **13** were geometrically optimized using Turbomole 7.0.1 (BP86/def‐SV(P)). A good agreement was observed between the optimized structures and their corresponding solid‐state structures (Table 1). Complexes **6** and **8** were constructed based on the solid‐state structure of complexes **1** and **7**. The Pd→B interactions were further analyzed using NBO/NLMO calculations. In all cases, an NBO donor/acceptor interaction was found between an occupied d‐orbital at Pd and an unoccupied *p*‐orbital at B (Figure 5). For all examined complexes no relevant η^2^(B,C)‐coordination was found in the NBO calculations. The Wiberg bond index for Pd,C_ipso_ was below 0.02, with the exception of Pd^0^ complexes **1** (0.0697) and **6** (0.0325). Reactivity studies of [(DPB)Pd]‐complexes presented in this paper thus appear to be unaffected from significant η^2^(B,C)‐coordination.

**Figure 6:**
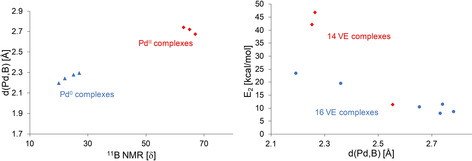
Left: correlation between solid state Pd,B distances and δ(^11^B). Right: correlation between calculated Pd,B distances and NBO stabilizing energies.

The NBO stabilizing energy of this Pd→B interaction varied depending on the Pd oxidation state. For Pd^II^→B interactions, a narrow range of NBO stabilizing energies between 8.04 and 11.46 kcal mol^−1^ was observed. Surprisingly, generation of cationic complexes (**9**, **10‐*endo***), exchange of chloro‐ligands by bromide (**8**) or iodide/aryl (**5**) had very little effect. In the case of Pd^0^→B interactions, significantly higher NBO stabilizing energies of 19.53–46.83 kcal mol^−1^ were found. Regardless of the oxidation state at Pd an approximately linear correlation between the Pd,B distance and the NBO stabilizing energy (*E*_2_) associated with the Pd,B interaction was observed (Figure 6) for 16 valence electron (VE) complexes **1**, **5**, **7**, **8**, **10** and **13**. The Pd,B distance appears to be dictated by the Pd,B bond strength, and not by constraints imposed by the chelating ligand. Substitution of PPh_2_‐groups (**6**) by PCy_2_‐groups (**3**) had only a minor effect. The *E*_2_ values for the Pd^0^→B interaction in the 14 VE complexes **3** (46.83 kcal mol^−1^) and **6** (42.12 kcal mol^−1^) significantly deviate from this correlation and are almost twice as much as for 16 VE complexes **1** (23.46 kcal mol^−1^) and **13** (19.53 kcal mol^−1^). Neither the ^11^B NMR chemical shift, Pd,B distance or pyramidalization at B indicate a change of the Pd^0^→B interaction strength in this magnitude between the 14 VE and the 16 VE complexes (Table 1). This discrepancy might be explained by the difficulty to compare the 2^nd^ order perturbation interaction energies from NBO analysis from 14 VE with 16 VE complexes.

The ^11^B NMR resonances are shifted linearly towards higher field with an increasing Pd,B distance for Pd^0^ complexes, regardless of the valence electron count at the Pd center (Figure 6). Complex [(^Ph^DPB^Ph^)Pd^0^(PPh_3_)] (**2**) reported by Kameo and Bourissou3e also fits perfectly into this correlation (d(Pd,B)=2.294(2) Å, *δ*(^11^B) 27 ppm). In contrast, the ^11^B NMR resonance shifts linearly towards lower field with an increasing Pd,B distance in case of Pd^II^ complexes. ^11^B NMR spectroscopy therefore can be used as a tool to assess the strength of Pd→B interactions within a given ligand system, provided that the oxidation state at the Pd center is taken into account. However, given the difficulty to determine the precise *δ*(^11^B) of [(DPB)Pd^II^] complexes (poor solubility and *ω*_1/2_ >1000 Hz ), a certain error for weak Pd^II^→B interactions needs to be factored in.^26^

**Scheme 4:**
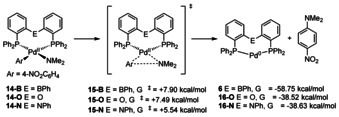
Reductive elimination of *N*,*N*‐dimethyl‐4‐nitroaniline from PEP complexes **14‐B**, **14‐O** and **14‐N**.

**Figure 7:**
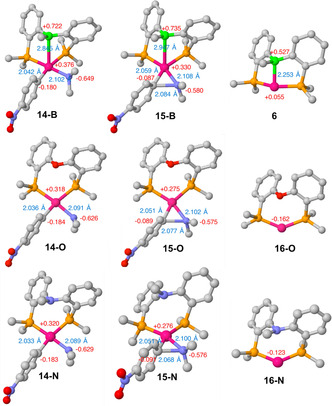
Calculated intermediates of reductive elimination from **14‐B** (top), **14‐O** (middle) and **14‐N** (bottom). For clarity the H atoms are omitted, and only the C_*ipso*_ atoms of the Ph‐groups at B and P are shown. Red: NPA charges, blue: bond distances.

Quantum chemical calculations (DFT) were used to model the inner‐sphere reductive elimination of *N*,*N*‐dimethyl‐4‐nitroaniline from complex **14‐B** (Scheme 4). C−N bond formation is predicted to proceed via an inner sphere reductive elimination with a low activation barrier of Δ*G* ^≠^=+7.90 kcal mol^−1^ (transition state **15‐B**), yielding Pd^0^ complex **6** and *N*,*N*‐dimethyl‐4‐nitroaniline (overall Δ*G*=−58.75 kcal mol^−1^). In order to understand how the Pd^II^→B interaction affects the reductive elimination, the reaction was also modeled for bis[(2‐diphenylphosphino)phenyl]ether (DPEphos) complex **14‐O** and diphosphinoamine complex **14‐N**. DPEphos is well established as an effective ligand in palladium catalyzed Buchwald–Hartwig‐type coupling reactions,^27^ and commands very similar structural features to ^Ph^DPB^Ph^ (Table 2). However, DPEphos cannot mimic the potential steric effect of the B−Ph group on the coordinated reactive ligands. For this reason, the diphosphinoamine ligand (*o*‐PPh_2_C_6_H_4_)_2_NPh^28^ has also been included in the theoretical considerations, as its *N*‐Ph bridgehead gives a good model of the B‐Ph group in **14‐B**. Elimination of *N*,*N*‐dimethyl‐4‐nitroaniline from complexes **14‐O** and **14‐N** gave very similar Gibbs free reaction energies of Δ*G*=−38.52 kcal mol^−1^ and Δ*G*=−38.63 kcal mol^−1^, respectively. No Pd^0/II^→E interactions were observed in complexes featuring DPEphos and the diphosphinoamine ligand (Table 2, WBI(Pd,E)=0.005, E=O, N). Given the high structural similarity of complexes **6**, **16‐O** and **16‐N** the increase of Δ*G* by ca. 20 kcal mol^−1^ in case of the ^Ph^DPB^Ph^ ligand is a good approximation for the increase of the Pd^0^→B interaction strength in **6** compared to the Pd^II^→B interaction strength in complex **14‐B**. When switching from ^Ph^DBP^Ph^ to DPEphos, a small decrease of ΔΔ*G* ^≠^=0.41 kcal mol^−1^ was found for the reductive elimination barrier (Scheme 4). This was surprising, as a more facile reductive elimination was expected from **14‐B** than from **14‐O**, due to 1) an electronic effect by Pd→B coordination and 2) increased steric bulk of the DPB ligand imposed by the B‐Ph group. In case of diphosphinoamine complex **14‐N** the reductive elimination barrier decreased to Δ*G* ^≠^=5.54 kcal mol^−1^ (ΔΔ*G* ^≠^=2.46 kcal mol^−1^), possibly as a result of the increased steric pressure imposed by the *N*‐Ph group (Table 2). Reductive elimination from **14‐E** (E=B, O, N) proceeds via structurally early transition‐state **15‐E** (Figure 7).

**Table 2:** Computational analysis of C−N bond formation from complexes **14‐B**, **14‐O** and **14‐N**.^[a]^

E=B, O, N	**14‐B**	**15‐B**	**6**	**14‐O**	**15‐O**	**16‐O**	**14‐N**	**15‐N**	**16‐N**
*d*(Pd,E) [Å]	2.845	2.947	2.253	3.343	3.349	2.955	3.360	3.381	3.023
*d*(C,N) [Å]	2.904	2.084	–	2.816	2.077	–	2.801	2.068	–
*d*(Pd,C) [Å]	2.042	2.059	–	2.036	2.051	–	2.033	2.051	–
*d*(Pd,N) [Å]	2.102	2.108	–	2.091	2.102	–	2.089	2.100	–
∢(P,Pd,P) [°]	101.2	101.0	147.1	100.4	102.0	136.4	97.5	98.8	132.9
q(Pd) ^[b]^	+0.376	+0.330	+0.055	+0.318	+0.275	−0.162	+0.320	+0.276	−0.123
q(E)^[b]^	+0.722	+0.735	+0.527	−0.498	−0.496	−0.485	−0.448	−0.448	−0.444
WBI(Pd,E)^[c]^	0.193	0.162	0.460	0.005	0.005	0.005	0.005	0.005	0.005
*Σ*B_*α*_ [°]	355.4	354.6	348.8	–	–	–	–	–	–

[a] Structure optimization: Turbomole 7.0.1, BP86/def‐SV(P); NBO analysis: Gaussian 09/NBO 6.0, BP86/6‐31G(d), MWB10 (P), MWB28 (Pd). [b] Natural population analysis (NPA) charge. [c] Wiberg bond index.

Unexpectedly, the Pd→B interaction is slightly weakened in transition‐state **15‐B**, compared to starting complex **14‐B**, as indicated by a slightly elongated Pd,B distance (2.947 Å) in **15‐B** compared to **14‐B** (2.906 Å). Similarly, the Wiberg bond index for the Pd→B interaction is reduced to 0.162 in **15‐B** (**14‐B**: 0.176), and the NPA charge at the borane remains unchanged (**14‐B**: +0.737 vs. **15‐B**: +0.735). The increase of the Pd→B interaction strength occurs after the reductive elimination, explaining why the inner‐sphere reductive elimination of the C−N bond does not kinetically profit from the substantial increase of the Pd→B strength in the course of the reaction.

To rule out effects originating from restraints imposed by a chelating ligand frame work, the reductive elimination of *N*,*N*‐dimethyl‐4‐nitroaniline was also modeled using *cis*‐[(PMe_3_)_2_Pd^II^(4‐NO_2_C_6_H_4_)NMe_2_] (**17**, Δ*G*=37.47 kcal mol^−1^) and its BH_3_ adduct [(PMe_3_)_2_(BH_3_)Pd^II^(4‐NO_2_C_6_H_4_)NMe_2_] (**17‐B**, Δ*G*=49.19 kcal mol^−1^) as substrates (cf. Scheme S1). Again, a more favorable transition state was found for the acceptor free complex **17** (Δ*G* ^≠^=+7.35 kcal mol^−1^), than for the borane adduct **17‐B** (Δ*G* ^≠^=+8.55 kcal mol^−1^).

## Conclusions

The strength of Pd→B interactions in [(DPB)Pd] complexes depends primarily on the oxidation state of Pd. In contrast, modifications of the DPB ligand or co‐ligands have only a minor effect. ^11^B NMR spectroscopy has been established as a useful tool to assess the strength of Pd→B interactions in solution. Reaction of lithium amides with [(^Ph^DPB^Ph^)Pd^II^(4‐NO_2_C_6_H_4_)I] (**5**) chemoselectively yields the C‐N coupling product and [(^Ph^DPB^Ph^)Pd^0^] (**6**). Inner‐sphere reductive C−N bond elimination was modelled with DFT methods for the ^Ph^DPB^Ph^ ligand. In contrast to reports on acceptor promoted outer‐sphere reductive C−N bond elimination,5b, 17 no significant effect of the borane acceptor on the inner‐sphere reductive elimination rate was found. This is explained by the fact that the strengthening of the Pd→B bond occurs after the reductive elimination.

## Experimental Section

### General

All manipulations were performed under an argon atmosphere using standard Schlenk line and glovebox techniques. Glassware was oven dried at 120 °C overnight and dried with a heat gun under vacuum prior to use. Tetrahydrofuran was dried by an MBraun solvent purification system. Benzene and *n*‐hexane were dried over sodium, distilled under argon prior to use and stored over activated molecular sieves (4 Å).

CD_2_Cl_2_ and C_6_D_6_ were degassed employing the freeze‐pump‐thaw technique and stored over activated molecular sieves (4 Å). [D_8_]THF was dried over activated molecular sieves (3 Å), distilled under an argon atmosphere and degassed employing the freeze‐pump‐thaw technique. ^Ph^DPB^Ph^, [(^Ph^DPB^Ph^OAc)Pd(C_3_H_5_)] (**4**), [(^Ph^DPB^Ph^)Pd(4‐NO_2_C_6_H_4_)I] (**5**) and [{(*o*‐PPh_2_C_6_H_4_)_2_BPh}PdI] (**12**) were synthesized according to published procedures.3b, 9d

NMR‐experiments were performed in Wilmad^®^ quick pressure valve NMR tubes. ^1^H, ^11^B{^1^H}, ^13^C{^1^H}, ^19^F{^1^H}, and ^31^P{^1^H} NMR spectra were recorded on a Bruker Avance II (400.1 MHz, probe: BBO) or a Bruker Avance (400.3 MHz, probe: ATM BBFO) spectrometer. ^1^H and ^13^C{^1^H} NMR spectra were referenced to residual solvent resonances as implemented in MesReNova 10.0.2. Infrared spectra were recorded on an Avatar 360 FT‐IR E.S.P. device by Nicolet. CHN combustion analysis were carried out on an Elementar EL device by Elementar Analysesysteme GmbH.

Deposition Number(s) 1987620 (**7**), 1987625 (**9**) and 1987626 (**10**)  contain(s) the supplementary crystallographic data for this paper. These data are provided free of charge by the joint Cambridge Crystallographic Data Centre and Fachinformationszentrum Karlsruhe Access Structures service www.ccdc.cam.ac.uk/structures.

### Reactivity studies

A solution of the respective lithium amide (5.7 μmol, 1.1 equiv) in [D_8_]THF (0.25 mL) was added dropwise over a period of 4 min to a stirred solution of nitroarene complex **5** (5.0 mg, 5.2 μmol, 1.0 equiv) in [D_8_]THF (0.25 mL). The resulting mixture was stirred for another 5 min and then transferred into an NMR tube. Reductive elimination was monitored by ^31^P NMR spectroscopy.

### Synthesis of [(^Ph^DPB^Ph^)PdCl_2_] (7)

CH_2_Cl_2_ (8 mL) was added to a mixture of ^Ph^DPB^Ph^ (400 mg, 0.665 mmol, 1.0 equiv) and [(cod)PdCl_2_] (187 mg, 0.665 mmol, 1.0 equiv). The mixture was stirred for 30 min at room temperature. Yellow crystals (380 mg, 0.482 mmol, 74 %) were formed by overlaying the solution *n*‐pentane (16 mL). Single crystals suitable for X‐ray diffraction were grown from a solution of [(cod)PdCl_2_] (9.7 mg, 34 μmol, 1.0 equiv) and ^Ph^DPB^Ph^ (21.2 mg, 34.7 μmol, 1.0 equiv) in CD_2_Cl_2_ (0.7 mL) overlaid with benzene (0.3 mL). ^11^B and ^13^C NMR data have not been collected due to poor solubility. ^1^H NMR (400.13 MHz, CD_2_Cl_2_, 25 °C): *δ* 7.81–7.76 (m, 2 H), 7.55 (tdd, *J=*7.3, 3.0, 1.1 Hz, 3 H), 7.50–7.46 (m, 3 H), 7.46–7.38 (m, 6 H), 7.35–7.14 (m, 13 H), 6.97–6.78 (m, 5 H), 5.32 (s, 2 H, CH_2_Cl_2_). ^31^P{^1^H} NMR (161.98 MHz, CD_2_Cl_2_, 26 °C): *δ* 44.5 (s, w_1/2_=570 Hz). IR (KBr): $\tilde{\nu }$ =3643‐3284 (w), 3049 (w), 1587 (w), 1497 (m), 1433 (vs., sh), 1223 (s), 1158 (vw), 1128 (w), 1093 (vs.), 987 (w), 889 (vw), 864 (vw), 754 (s), 744 (s), 733 (m), 688 (vs.), 667 (w), 611 (m), 600 (s), 542 (m), 523 (vs.), 505 (m) cm^−1^. Elemental analysis calcd (%) for C_42_H_33_BCl_2_P_2_Pd⋅CH_2_Cl_2_: C 59.18, H 4.04, found: C 59.61, H 4.33.

### Synthesis of [(^Ph^DPB^Ph^)PdBr_2_] (8)

The ^Ph^DPB^Ph^ ligand (200 mg, 0.328 mmol, 1.0 equiv) and [(cod)PdBr_2_] (122.7 mg, 0.328 mmol, 1.0 equiv) were solved in DCM (10 mL) and stirred at r.t. for 30 min. The solution was overlaid with n‐hexane (20 mL) yielding title compound **8** as orange crystals (192.0 mg, 0.219 mmol, 67 %). ^11^B and ^13^C NMR data have not been collected due to poor solubility. ^1^H NMR (400.30 MHz, CD_2_Cl_2_): *δ* 7.85–7.76 (m, 3 H), 7.59–7.19 (m, 30 H). ^31^P{^1^H} NMR (162.04 MHz, CD_2_Cl_2_): *δ* 45.2 (bs, 1P, w_1/2_=450 Hz), 38.1 (bs, 1P, w_1/2_=450 Hz). IR (KBr): $\tilde{\nu }$ =3424 (s), 3048 (m), 1621 (w), 1587 (w), 1478 (m), 1455 (w), 1432 (s), 1311 (w), 1237 (w), 1220 (s), 1205 (m), 1187 (m), 1153 (w), 1126 (m), 1092 (s), 1027 (w), 1000 (m), 887 (w), 863 (w), 753 (s), 741 (s), 713 (m), 699 (s), 690 (s), 667 (m), 610 (s), 600 (s), 539 (s), 522 (s), 505 (s), 465 (m) cm^−1^. Elemental analysis calcd (%) for C_42_H_33_BBr_2_P_2_Pd⋅0.25CH_2_Cl_2_: C 56.51; H 3.76, found: C 56.72, H 3.83.

### Synthesis of [(^Ph^DPB^Ph^)PdCl]SbF_6_ (9)

Complex **7** (200 mg, 254 μmol, 1.0 equiv) and AgSbF_6_ (87.2 mg, 254 μmol, 1.0 equiv) were stirred in DCM (15 mL) for 40 minutes. The suspension was filtered through a syringe filter (0.2 μm, PTFE membrane). The clear solution was overlaid with n‐hexane (30 mL) yielding the title compound **9** as long colorless needles (128 mg 130 μmol, 51 %). ^1^H NMR (400.30 MHz, CD_2_Cl_2_): *δ* 7.97–7.92 (m, 2 H), 7.80 (tdd, *J*=7.5, 2.8, 0.9 Hz, 2 H), 7.69 (dd, *J*=7.6, 2.6 Hz, 2 H), 7.65 (t, *J*=7.5 Hz, 2 H), 7.55 (tt, *J*=7.4, 1.4 Hz, 1 H), 7.47–7.34 (m, 6 H), 7.27–7.16 (m, 10 H), 7.00 (dt, *J*=7.6, 2.4 Hz, 4 H), 6.83 (dd, *J*=12.4, 7.9 Hz, 4 H). ^11^B{^1^H} NMR (128.43 MHz, CD_2_Cl_2_): *δ*=65 (bs, w_1/2_=1900±300 Hz). ^13^C{^1^H} NMR (100.67 MHz, CD_2_Cl_2_): *δ*=*δ* 141.79, 135.43 (d, *J=*8.5 Hz), 134.88 (d, *J=*11.1 ‐Hz), 134.25, 133.69 (d, *J=*19.5 Hz), 133.22 (d, *J=*17.4 Hz), 132.49 (d, *J=*3.7 Hz), 129.67 (d, *J=*8.9 Hz), 129.33–128.82 (m), 128.10, 127.13, 126.74, 126.16. ^31^P{^1^H} NMR (162.04 MHz, CD_2_Cl_2_): *δ* 49.9 (s, w_1/2_=30 Hz). IR (KBr): $\tilde{\nu }$ =3441 (s), 3058 (w), 1588 (w), 1482 (w), 1435 (s), 1230 (m), 1200 (w), 1125 (w), 1034 (m), 1001 (w), 867 (vw), 752 (s), 702 (s), 692 (s), 659 (vs.), 614 (m), 538 (s), 517 (s), 697 (w) cm^−1^. Elemental analysis calcd (%) for C_42_H_33_BClF_6_P_2_PdSb⋅0.25 C_6_H_14_: C 51.75, H 3.64, found: C 51.77, H 3.785.

### Synthesis of [(^Ph^DPB^Ph^)Pd(C_3_H_5_)]SbF_6_ (10)

Allyl complex **4** (120 mg, 143 μmol, 1.0 equiv) and AgSbF_6_ (49.0 mg, 143 μmol, 1.0 equiv) were solved in CH_2_Cl_2_ (7 mL) and stirred at r.t. for 20 min. The suspension was filtered through a syringe filter (0.2 μm, PTFE membrane). The clear solution was overlaid with n‐hexane (10 mL). The obtained crystals showed insufficient purity and were crystallized again under the same conditions yielding **10** as slightly yellow crystals (50.2 mg, 53.8 μmol, 38 %). ^1^H NMR (400.30 MHz, CD_2_Cl_2_): *δ* 7.72–7.59 (m, 4 H), 7.58–7.53 (m, 2 H), 7.53–7.44 (m, 13 H), 7.43–7.29 (m, 6 H), 7.23–7.15 (m, 2 H), 7.05–6.87 (m, 5.5 H), 6.78–6.67 (bs, 2 H), 5.88–5.70 (bs, 0.7 H), 3.77–3.61 (bs, 1.3 H), 3.59–3.33 (bs, 1.3 H), 3.03–2.85 (bs, 0.9 H), 2.49–2.29 (bs, 1.2 H) (fractional integrals are a result from signal splitting caused by a dynamic process). ^11^B{^1^H} NMR (128.38 MHz, CD_2_Cl_2_): *δ* 64 (bs, w_1/2_=1550±50 Hz). ^13^C{^1^H} NMR (100.67 MHz, CD_2_Cl_2_): *δ* 141.1, 140.2, 136.1, 135.5, 135.3, 135.0, 134.4, 134.3, 134.0, 133.2 (t, *J*=5.8 Hz), 132.3, 132.2, 132.1, 131.6, 131.5, 131.2, 131.0, 129.6 (t, *J*=5.3 Hz), 129.3, 128.9, 123.1, 80.4, 80.2. ^31^P{^1^H} NMR (162.04 MHz, CD_2_Cl_2_): *δ* 28.1 (s, 0.6P), 26.9 (s, 0.4P). IR (KBr): $\tilde{\nu }$ =3430 (s), 3000 (m), 1588 (m), 1480 (m), 1458 (w), 1434 (s), 1268 (m), 1227 (s), 1127 (m), 1095 (m), 1031 (w), 999 (w), 950 (vw), 875 (w), 772 (w), 754 (m), 742 (m), 733 (m), 695 (s), 659 (vs.), 609 (s), 537 (m), 521 (s), 478 (w), 430 (w) cm^−1^. Elemental analysis calcd (%) for C_46_H_40_BCl_2_F_6_P_2_PdSb: C 51.22, H 3.74, found: C 51.04, H, 3.86.

### Synthesis of [(^Ph^DPB^Ph^)Pd] (6)

A solution of LiNMe_2_⋅THF (0.7 mg, 6 μmol, 1.1 equiv) in [D_8_]THF (0.25 mL) was added over a period of 3 min to a solution of complex **5** (5.0 mg, 5 μmol, 1 equiv) in [D_8_]THF (0.25 mL). The combined solutions were transferred to an NMR tube and NMR spectra were recorded after 1.5 and 4.5 h. ^11^B{^1^H} NMR (128.38 MHz, [D_8_]THF): *δ* 19 (bs, w_1/2_=550 Hz±50 Hz). ^31^P{^1^H} NMR (162.04 MHz, [D_8_]THF): *δ* 30.93 (s).

### Synthesis of [(^Ph^DPB^Ph^)Pd(PMe_3_)] (11)

A solution of PhLi (3.2 mg, 38 μmol, 1.2 equiv) in THF (0.5 mL) was slowly added to a solution of complex **12** (25 mg, 33 μmol, 1.0 equiv) in THF (0.5 mL). After stirring for 10 min at r.t. a solution of PMe_3_ in toluene (1.0 m, 50 μL, 50 μmol, 1.5 equiv) was added. The precipitate was removed by filtration and the solution was concentrated in vacuo. The resulting solid was washed with pentane and dried in vacuo (20.7 mg, 26.1 μmol, 79 %). ^1^H NMR (400.13 MHz, C_6_D_6_): *δ* 8.34 (d, 2 H, *J*=7.8 Hz), 7.69–7.58 (m, 4 H), 7.44–7.37 (m, 2 H), 7.36–7.28 (m, 4 H, Ar‐*H*), 7.12 (t, 2 H, *J*=6.7 Hz), 7.09–7.05 (m, 13 H), 6.85 (m, 2 H), 6.68 (pt, 4 H, *J*=7.8 Hz), 0.64 (d, ^2^ *J*_P‐H_=5.0 Hz, 9 H, PMe_3_). ^11^B{^1^H} NMR (128.38 MHz, C_6_D_6_): *δ* 25 (bs, w_1/2_=740 Hz ±50 Hz). ^13^C{^1^H} NMR (100.62 MHz, C_6_D_6_): *δ* 168.7 (bs), 143.2 (d, *J*=16.3 Hz), 143.0 (d, *J*=16.3 Hz), 141.5 (td, *J*=15.2, 2.0 Hz), 138.9 (t, *J*=13.5 Hz), 135.8 (t, *J*=6.4 Hz), 135.7 (t, *J*=2.7 Hz), 133.5 (t, *J*=7.7 Hz), 133.0 (dt, *J*=16.7, 5.0 Hz), 132.3 (s), 132.3 (s), 132.4 (t, *J*=6.7 Hz), 129.5 (s), 129.0 (s), 128.6 (s), 127.2 (s), 126.1 (t, *J*=2.8 Hz), 125.2 (s), 18.1 (dt, *J*=11.8, 2.2 Hz, PMe_3_). ^31^P{^1^H} NMR (162.04 MHz, C_6_D_6_): *δ* 35.44 (d, ^2^ *J*_P‐P_=14.1 Hz, 2P, ArPPh_2_), −40.13 (t, ^2^ *J*_P‐P_=14.2 Hz, 1P, PMe_3_).

## Conflict of interest

The authors declare no conflict of interest.

## Supporting information

As a service to our authors and readers, this journal provides supporting information supplied by the authors. Such materials are peer reviewed and may be re‐organized for online delivery, but are not copy‐edited or typeset. Technical support issues arising from supporting information (other than missing files) should be addressed to the authors.

Supplementary
