# Supplementary material for: Evaluation of Pd→B Interactions in Diphosphinoborane Complexes and Impact on Inner‐Sphere Reductive Elimination
Source: Chemistry. 2020 Sep 18;26(59):13436–44. doi: 10.1002/chem.202001189 (PMC7692930; doi:10.1002/chem.202001189)
Supplement: Supplementary file 1 — Supplementary [file CHEM-26-13436-s001.pdf]

Chemistry–A European Journal

Supporting Information

Evaluation of Pd→B Interactions in Diphosphinoborane Complexes and Impact on Inner-Sphere Reductive Elimination

Florian Ritter, Lukas John, Tobias Schindler, Julian P. Schroers, Simon Teeuwen, and Michael E. Tauchert*[a]

1 EXPERIMENTAL SECTION 1
2 SINGLE CRYSTAL X-RAY DIFFRACTION 2
3 COMPUTATIONAL STUDY 4
4 NMR SPECTRA 6
5 GEOMETRIES OF STATIONARY POINTS 18

1 Experimental Section

1.1 Reactivity studies: reaction of [(PhDPBPh)Pd^{II}(4-$NO_2C_6H_4$)I] **5** with lithium amides

Within a glove box a solution of the respective amide (5.7 µmol, 1.1 equiv.) in THF-d_8 (0.25 mL) was added dropwise over a period of 4 min to a stirred solution of nitroarene complex **5** (5.0 mg, 5.2 µmol, 1.0 equiv.) in THF-d_8 (0.25 mL). The resulting mixture was stirred for another 5 min and then transferred into a J-Young NMR tube. Reductive elimination was monitored by ^{31}P NMR spectroscopy.

Table S1. Reaction of **5** with 1.1 equiv. of lithium amides.

#	amide	*t* [h]	**6** [%]
1	LiNHtBu	2.0	11
2	LiNHtBu	5.5	14
3	LiNHtBu	68	37
4	LiNHtBu	96	46
5	$LiNCy_2$	3.0	26
6	$LiNMe_2$	1.0	84
7	$LiNMe_2$	4.5	100

1.2 VT-NMR

[(^{Ph}DPB^{Ph})$PdCl_2$] **7** (1.5 mg, 1.9 *µ*mol) was solved in CD_2Cl_2 (0.8 mL). ^{1}H and ^{31}P{^{1}H} NMR spectra were recorded at different temperatures. The temperatures and the ^{31}P chemical shifts are depicted in Table S2. Rate constants were determined by line shape analysis using Bruker's TopSpin 3.0 software.

Table S2: Temperatures, measured and simulated $^{31}P\{^1H\}$ NMR chemical shifts/signal shapes and simulated rate constants of **7**.

#	*T* [K]	*δ* [ppm] (signal shape)	$w_{1/2}$ [Hz] (simulated)	*δ* P1 [ppm] (simulated)	*δ* P2 [ppm] (simulated)	*k* [s^{-1}]
1	308.3	42.25–47.10 (br. sig.)	290	44.26	44.26	3557
2	298.9	39.02–48.17 (br. sig.)	344	44.86	43.43	1900
3	283.5	41.91 (s, br. sig.), 47.36 (s, br. sig.)	234	47.21	41.87	936
4	273.2	41.49 (s, br. sig.), 47.79 (s, br. sig.)	161	47.89	41.41	391
5	263.4	41.39 (s), 47.84 (s)	29	48.01	41.34	254
6	253.4	41.37 (s), 48.10 (s)	18	48.13	41.37	124
7	243.4	41.41 (s), 48.29 (s)	19	48.29	41.41	57

2 Single Crystal X-ray diffraction

Crystal data, data collection parameters and refinement results for **7**, **9** and **10** have been compiled in Table S3.

Intensity data for **7** and **10** was collected on a Bruker D8 goniometer with APEX CCD area detector in *ω*-scan mode using Mo-$K_α$ radiation ($λ$ = 0.71073 Å) from an Incoatec microsource with multilayer optics. A temperature of 100(2) K was maintained with the help of an Oxford Cryostream 700 instrument. Data were collected with SMART,[1] integrated with SAINT+[2] and corrected for absorption by multi-scan methods with SADABS.[3] Intensity data for **9** was collected on a Stoe Stadivari equipped with a Pilatus 3R 200K detector (Mo-$K_α$, $λ$ = 0.71073 Å). Data collection, reduction, cell refinement and spherical absorption correction were performed within Stoe X-Area.[4] Using Olex2,[5] the structures were solved with the ShelXS structure solution program employing direct methods.[6] Full-matrix least-square refinements based on F^2 were performed with SHELXL.[6] Non-hydrogen atoms were assigned anisotropic displacement parameters unless stated otherwise.

These data can be obtained free of charge from The Cambridge Crystallographic Data Centre via www.ccdc.cam.ac.uk/data_request/cif. The presentation of crystal structures was done with Ortep3.[7]

Table S3. Crystal data and structure refinement for **7**, **9** and **10**.

Complex / CCDC	**7** 1987620	**9** · 1 CH_2Cl_2 1987625	**10** · 1 CH_2Cl_2 1987626
Empirical formula	$C_{42}H_{33}BCl_2P_2Pd$	$C_{86}H_{70}B_2Cl_6F_{12}P_4Pd_2Sb_2$	$C_{46}H_{40}BCl_2F_6P_2PdSb$
Formula weight	787.73	2145.94	1078.58
Temperature/K	100(2)	100(2)	100(2)
Crystal system	Triclinic	Triclinic	Monoclinic
Space group	P-1	P-1	$P2_1/c$
a/Å	10.3478(8)	12.236(2)	12.059(2)
b/Å	10.9350(8)	13.207(3)	18.635(2)
c/Å	17.6683(13)	13.749(3)	19.780(2)
α/°	77.1150(10)	79.74(3)	90
β/°	76.6190(10)	72.99(3)	107.340(2)
γ/°	63.6820(10)	74.83(3)	90
Volume/Å^3	1726.7(2)	2038.2(8)	4242.8(8)
Z	2	1	4
ρ_{calc}g/cm^3	1.515	1.748	1.689
μ/mm^{-1}	0.816	1.439	1.322
F(000)	800.0	1060.0	2144.0
Crystal size/mm^3	0.08 x 0.05 x 0.04	0.25 x 0.17 x 0.12	0.12 × 0.12 × 0.08
2Θ range /°	4.442 to 52.976	1.61 to 26.59	3.07 to 53.1
Index ranges	$-12 \le h \le 12$ $-13 \le k \le 13$ $-22 \le l \le 22$	$-15 \le h \le 12$ $-16 \le k \le 16$ $-17 \le l \le 16$	$-15 \le h \le 15$ $-23 \le k \le 23$ $-24 \le l \le 24$
Refls collected	20998	15975	51175
Independent reflections	7120	8222	8800
Data/restraints/parameters	7120/0/433	8222/0/514	8800/0/532
GOF	1.039	0.922	1.008
Final R indexes [I>=2σ (I)]	R_1 = 0.0367, wR_2 = 0.0800	R_1 = 0.0371, wR_2 = 0.0825	R_1 = 0.0412, wR_2 = 0.0837
Final R indexes [all data]	R_1 = 0.0453, wR_2 = 0.0851	R_1 = 0.0569, wR_2 = 0.0870	R_1 = 0.676, wR_2 = 0.0952
Largest diff. peak/ hole / e Å^{-3}	0.63 / -0.64	0.81 / -1.05	0.60/-0.64

3 Computational Study

Geometry optimizations were carried out without any symmetry restrictions. The minimum on the potential energy surface was confirmed by the absence of an imaginary frequency in the vibrational spectrum. Geometry optimizations and frequency analysis were performed using Turbomole 7.0.1.[8] The RI approximation[9] was used applied throughout. Calculations were performed with the BP86[10] functional, utilizing a def-SV(P)[11] basis set. The transition state optimizations of **15-B** and **15-O** were carried out using the TRIM method maximizing the energy along one of the Hessian eigenvectors, while minimizing it in all other directions.[8] Complexes **1**, **3**, **5**, **7**, **9** and **13** were optimized using xyz-coordinates (excluding anion and solvent) from their solid-state structures.[12] Complex **8** was optimized based on the xyz-coordinates of **7** with the chloro ligands replaced by bromide. Optimization of complex **9** was based on the asymmetric unit of its crystal structure. Additionally, the respective diastereomer was optimized (**9:** dihedral angle C_{ipso}–B–Pd–Cl = -59.8 °; **9-isomer**: dihedral angle C_{ipso}–B–Pd–Cl = +5.0 °). Complex **9** was found 1.17 kcal/mol lower in energy and was used for further analysis. In case of **10** both isomers **10-*endo*** and **10-*exo*** were calculated based on the solid-state structure, with an energy preference of **10-*endo*** of 0.0032 kcal/mol. The ΔG value was calculated using the thermal correction implemented in Turbomole's freeh routine (scaling factor for BP/SVP: 0.9914, *T* = 298.15 K, *p* = 0.100 MPa). Further analysis was carried out based on the **10-*endo*** isomer. Structure optimization of complex **6** was based on the solid-state structure of **1** in which the pyridine ligand was removed.

Model complexes **17**, **17-B**, **19**, **19-B** (Scheme S1) were constructed in Gaussview using idealized coordination geometries (square-planar, square-pyramidal, T-shape and linear).

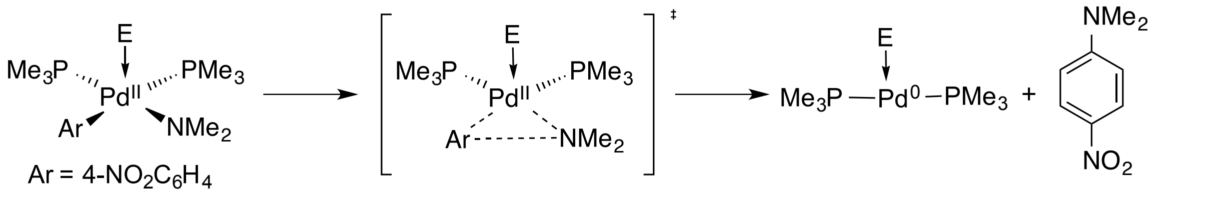

17 E = Ø
17-B E = BH_3

18 E = Ø, ΔG‡ = +7.35 kcal/mol
18-B E = BH_3, ΔG‡ = +8.55 kcal/mol

19 E = Ø, ΔG = -37.47 kcal/mol
19-B E = BH_3, ΔG = -49.19 kcal/mol

Scheme S1. Inner-sphere reductive elimination from model complexes **17** and **17-B**.

Electronic structures of complexes **1**, **3**, **5 - 11** and **13** were studied using Natural Bond Orbital analysis (NBO-6 program)[13] implemented in the Gaussian 09 suite of programs.[14] NBO and NLMO analyses were performed with the BP86 functional, utilizing 6-31G(d)[15] (H, B, C, N), MWB10 (P, Cl),[16] MWB28 (Pd, Br),[16] and MWB46 (I)[17] basis sets. Results are summarized in Table S4.

Table S4. Experimental and computational analysis of the Pd→B interaction.[a]

	d(Pd,B) [Å] (XRD / DFT)	d(Pd,C_{ipso}) [Å] (XRD / DFT)	ΣB_{α} [°] (XRD / DFT)	^{11}B NMR (δ, $\omega_{1/2}$)	E_2(Pd,B)[b] [kcal/mol]	% B[c]	% Pd[c]	occ. B[d]	occ. Pd[d]	B-hybrid
**7**	2.762(3) / 2.740	3.405(3) / 3.256	355 / 355	-	11.46	6.6	91.9	0.391	1.859	7.6% s, 92.4% p
**8**	- / 2.654	- / 3.292	- / 352	-	10.42	6.3	92.2	0.387	1.865	7.2% s, 92.7% p
**9**[e]	2.721(5) / 2.554	3.338(4) / 3.112	356 / 355	65 ppm 1900 Hz	11.41	5.4	92.9	0.400	1.870	7.2% s, 92.7% p
**10-*endo***	2.676(5) / 2.708	3.066(6) / 3.259	355 / 355	67 ppm 1400 Hz	8.04	3.7	93.9	0.360	1.887	6.7% s, 93.3% p
**5**	2.7402(4) / 2.781	3.346(4) / 3.440	354 / 351	63 ppm 3000 Hz	8.72	4.7	93.4	0.353	1.879	6.4% s, 93.6% p
**1**	2.194(3) / 2.193	2.463(3) / 2.865	346 / 346	20 ppm 400 Hz	23.46	16.0	78.7	0.618	1.666	11.6% s, 88.4% p
**13**	2.19(6) / 2.360	2.815(2) / 2.685	338 / 341	25 ppm 500 Hz	19.53	15.0	81.5	0.621	1.702	13.9% s, 86.1 % p
**3**	2.243(2) / 2.264	3.079(2) / 3.054	341 / 343	22 ppm 800 Hz	46.83	15.5	81.7	0.498	1.686	12.8% s, 87.2% p
**6**	- / 2.253	- / 2.768	- / 349	19 ppm 400 Hz	42.12	14.3	83.0	0.519	1.704	10.7% s, 89.3% p

[a] Structure optimization: Turbomole 7.0.1, BP86/def-SV(P); NBO analysis: Gaussian 09/NBO 6.0, BP86/6-31G(d), MWB28 (Pd, Br), MWB46 (I). [b] NBO stabilizing energy E_2 associated with the Pd→B interaction. [c] Contribution of the donor/acceptor NBO to the NLMO. [d] Occupancy of the donor/acceptor NBO. [e] Calculated structure parameters of **9** are based on the monomer.

4 NMR Spectra

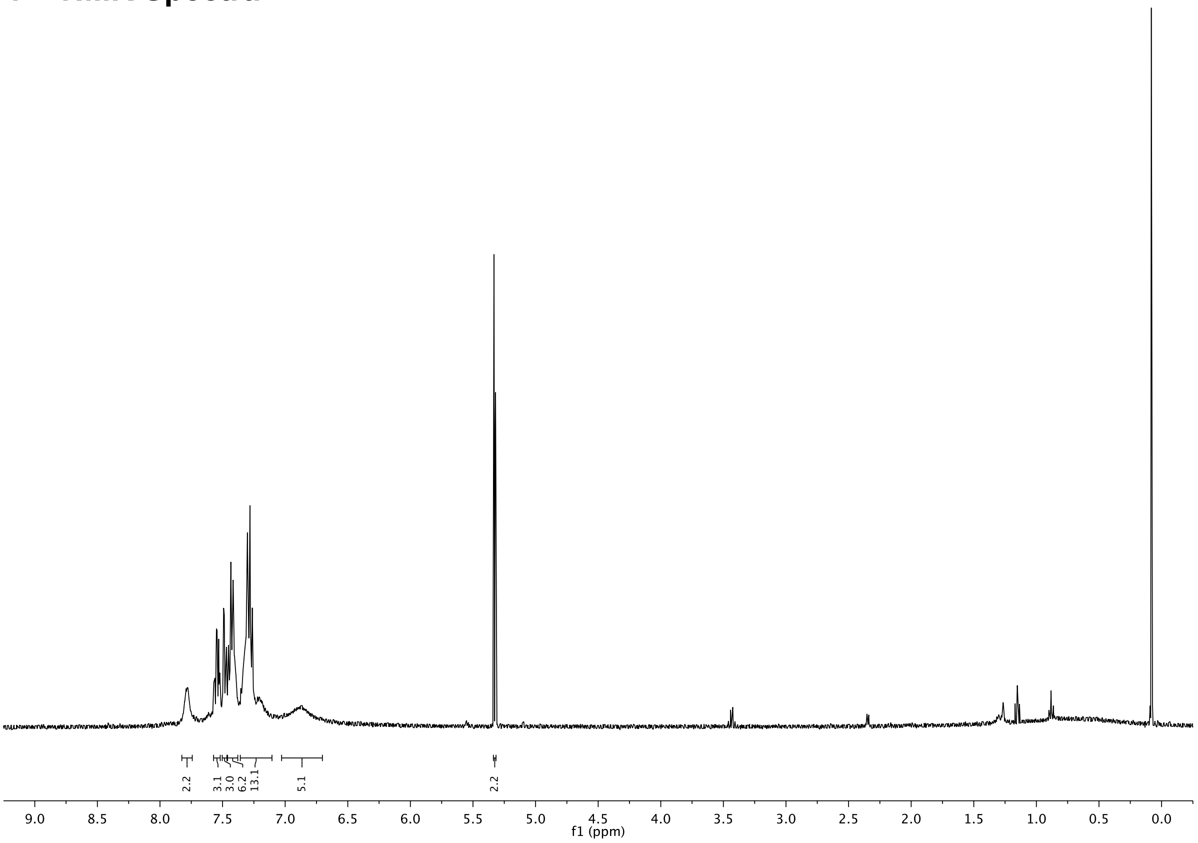

Figure S1. ^{1}H NMR (400 MHz, Methylene Chloride-d_2) [(^{Ph}DPB^{Ph})PdCl$_2$] **7**.

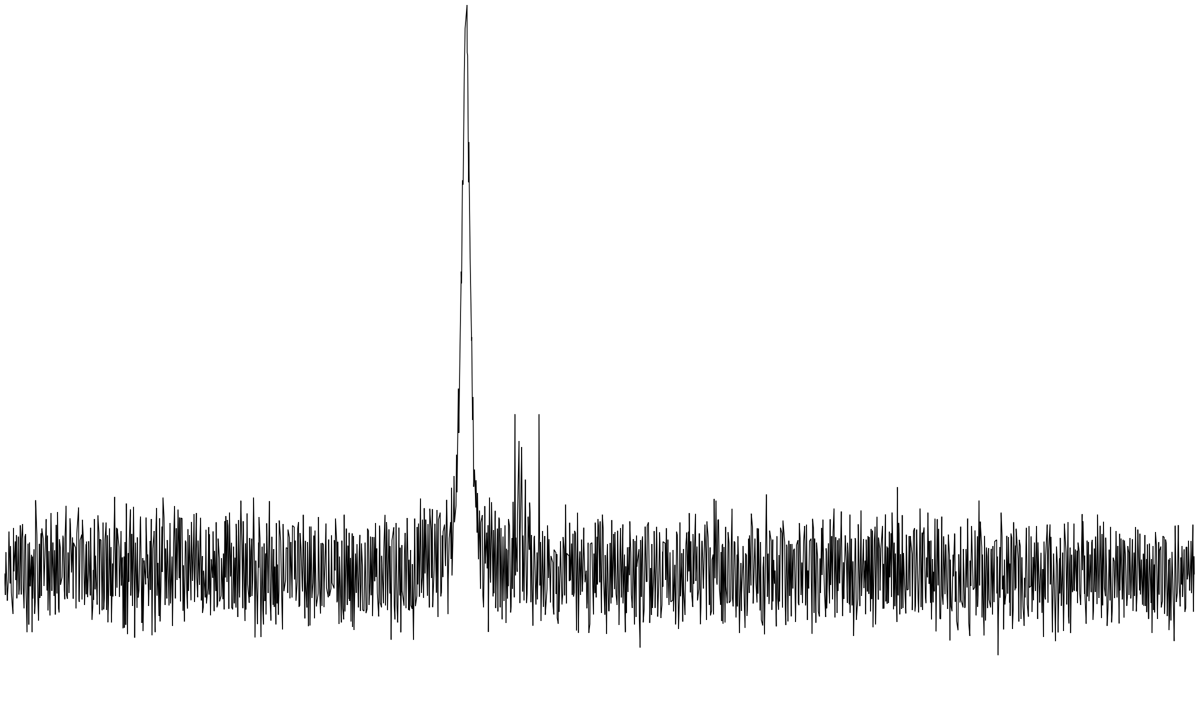

Figure S2. ^{31}P NMR (162 MHz, Methylene Chloride-d_2) [(^{Ph}DPB^{Ph})PdCl$_2$] **7**.

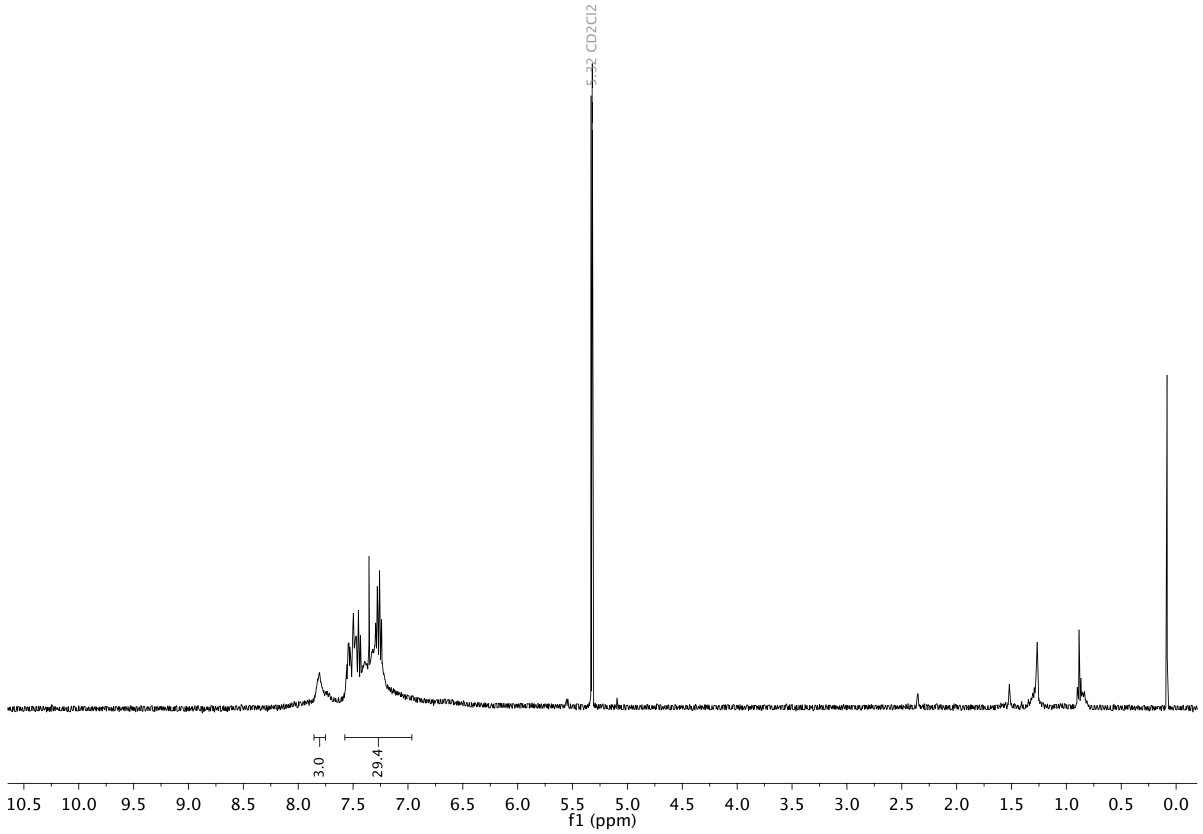

Figure S3. ^{1}H NMR (400 MHz, Methylene Chloride-d_2) [(^{Ph}DPB^{Ph})$PdBr_2$] **8.**

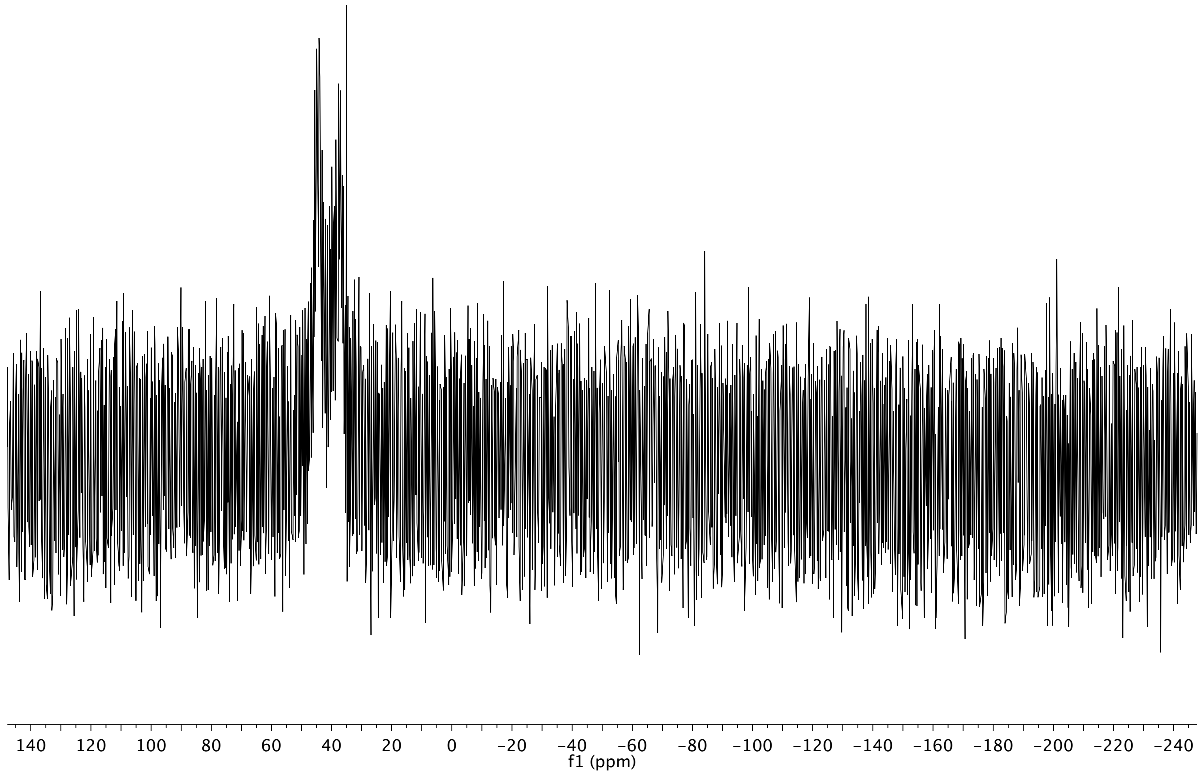

Figure S4. ^{31}P NMR (162 MHz, Methylene Chloride-d_2) [(^{Ph}DPB^{Ph})$PdBr_2$] **8.**

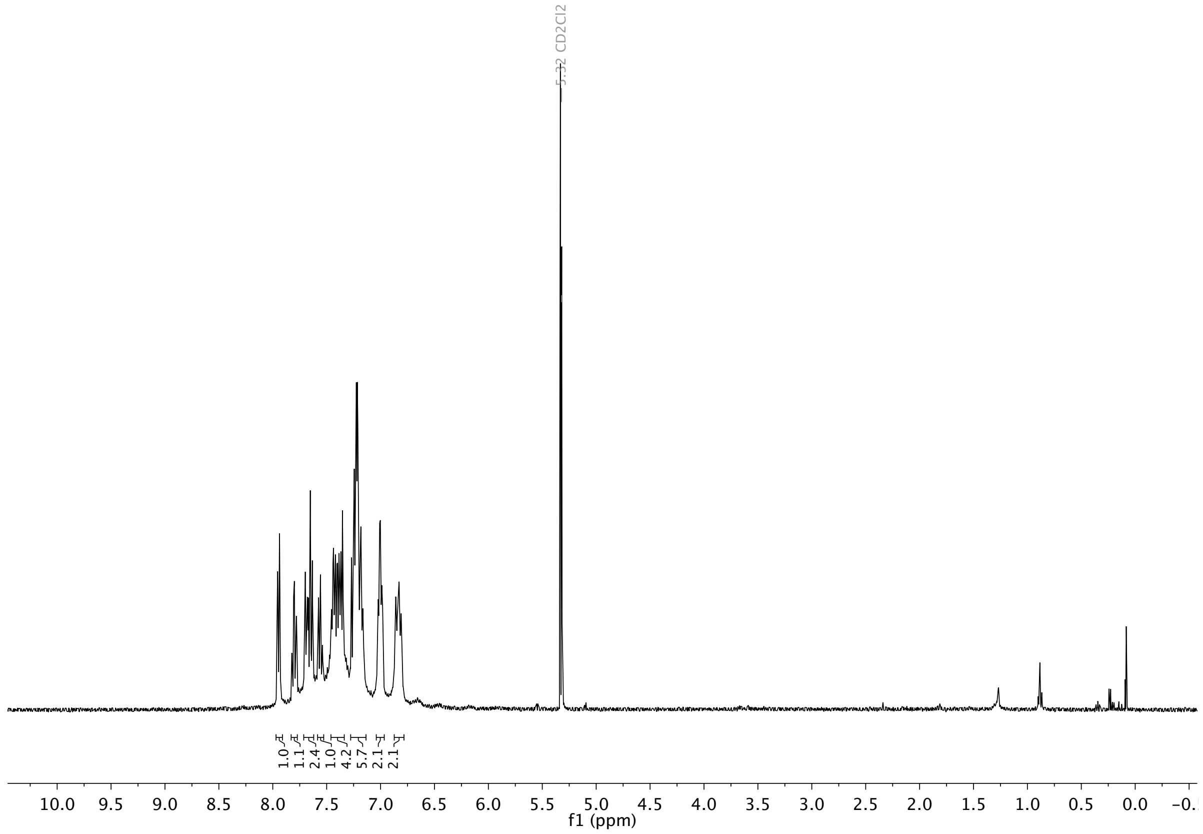

Figure S5. ^{1}H NMR (400 MHz, Methylene Chloride-d_2) [(^{Ph}DPB^{Ph})PdCl]SbF_6 **9**.

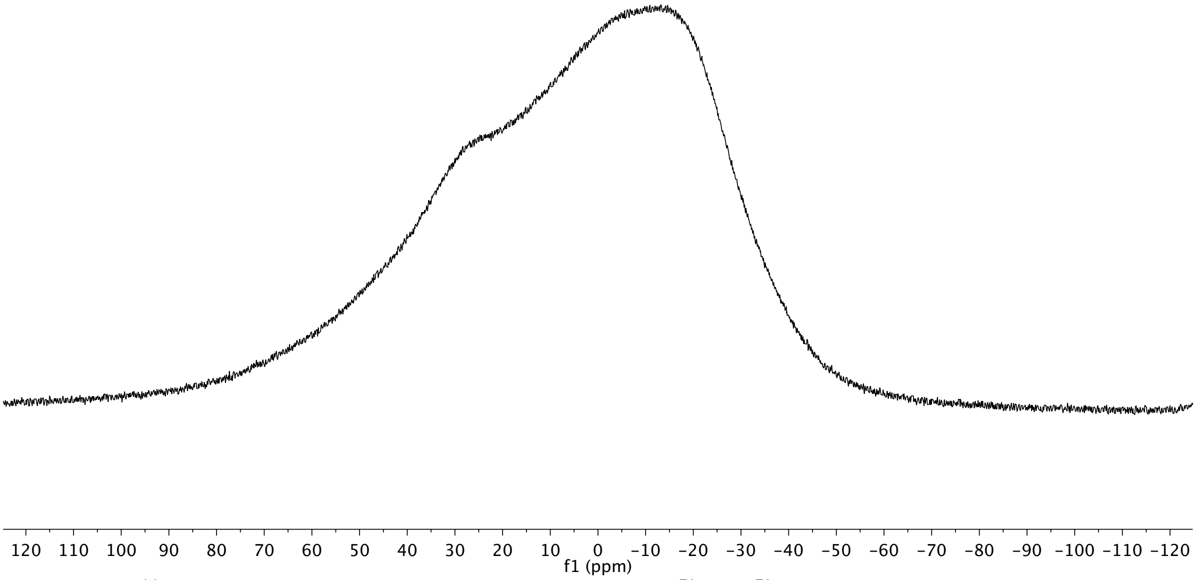

Figure S6. ^{11}B NMR (128 MHz, Methylene Chloride-d_2) [(^{Ph}DPB^{Ph})PdCl]SbF_6 **9**.

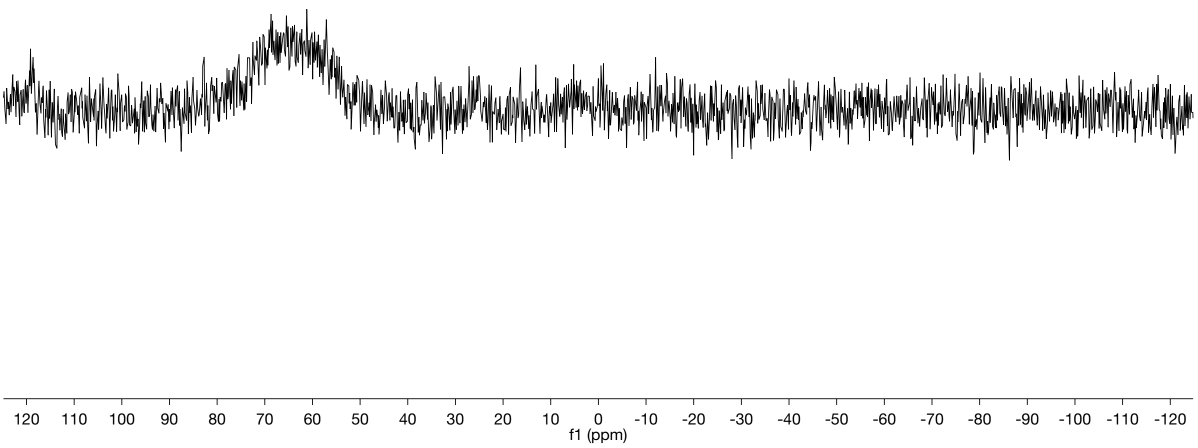

Figure S7. ^{11}B NMR (128 MHz, Methylene Chloride-d$_2$) $[(^{Ph}DPB^{Ph})PdCl]SbF_6$ **9** after background subtraction.

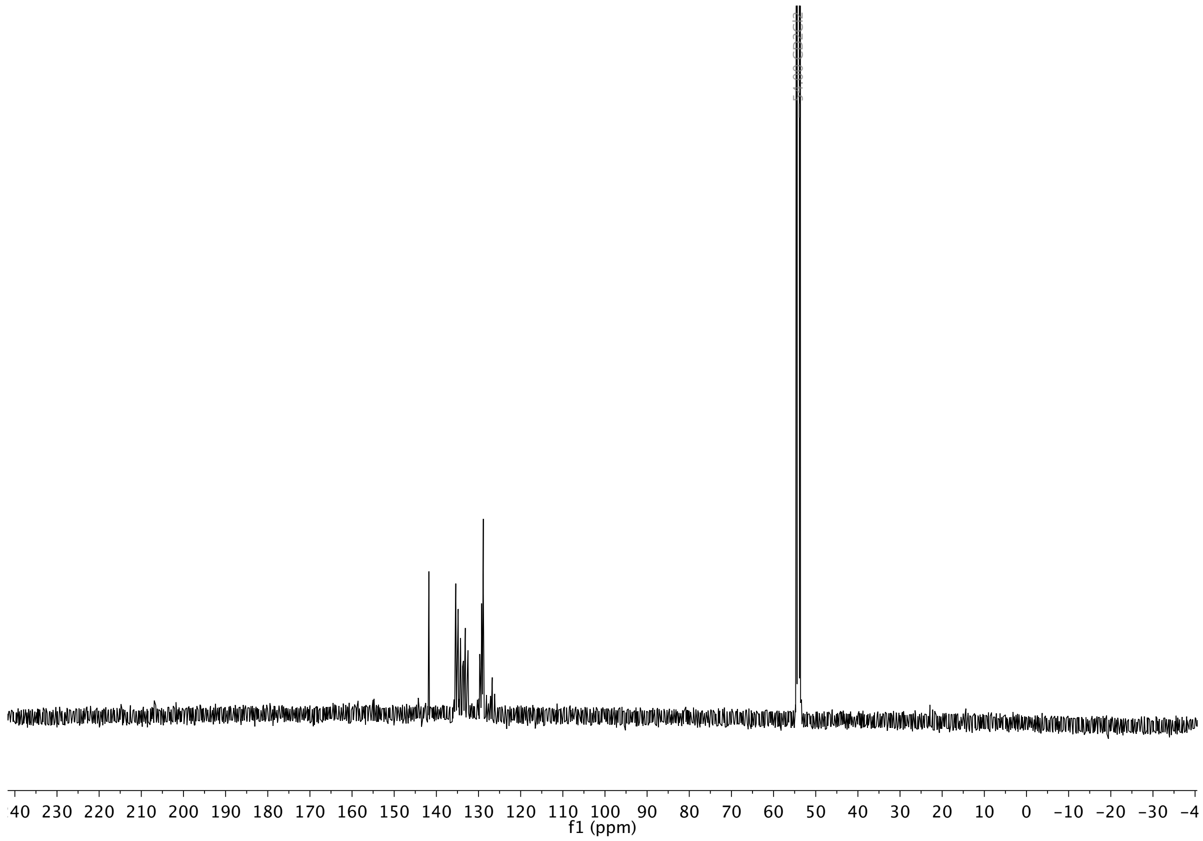

Figure S8. ^{13}C NMR (101 MHz, Methylene Chloride-d$_2$) $[(^{Ph}DPB^{Ph})PdCl]SbF_6$ **9**.

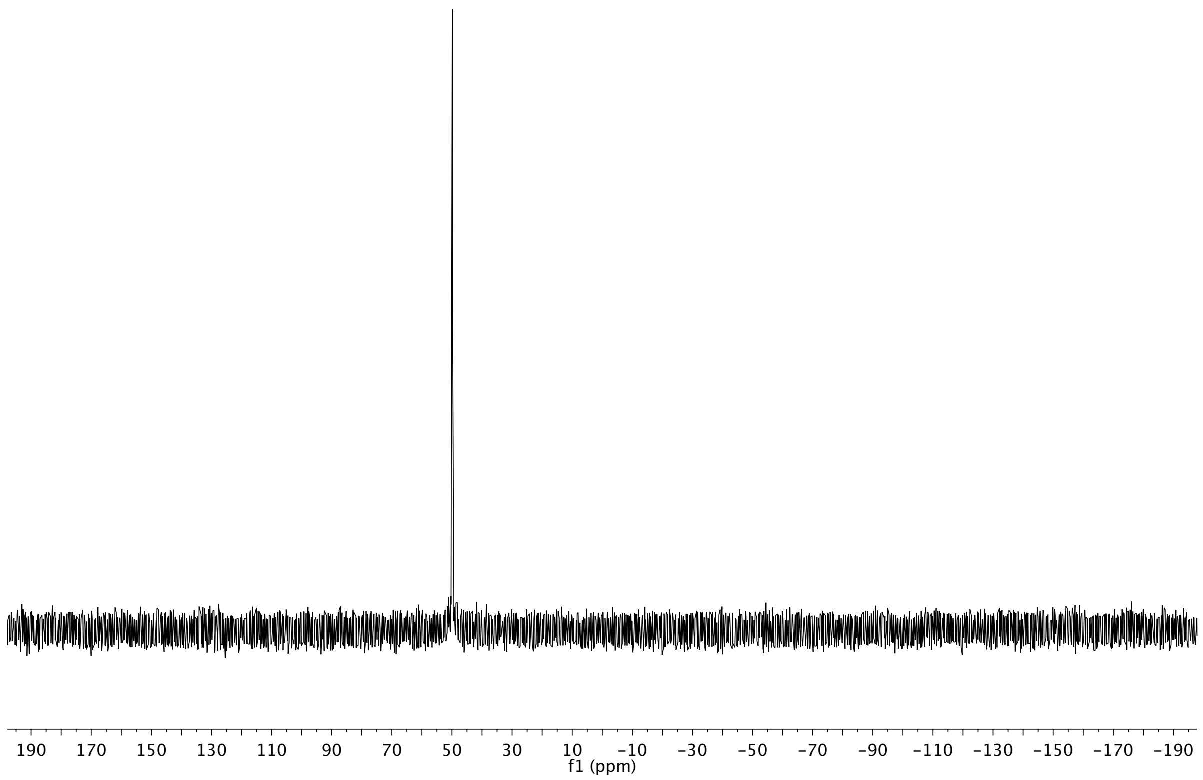

Figure S9. ^{31}P NMR (162 MHz, Methylene Chloride-d_2) [($^{Ph}DPB^{Ph}$)PdCl]SbF_6 **9**.

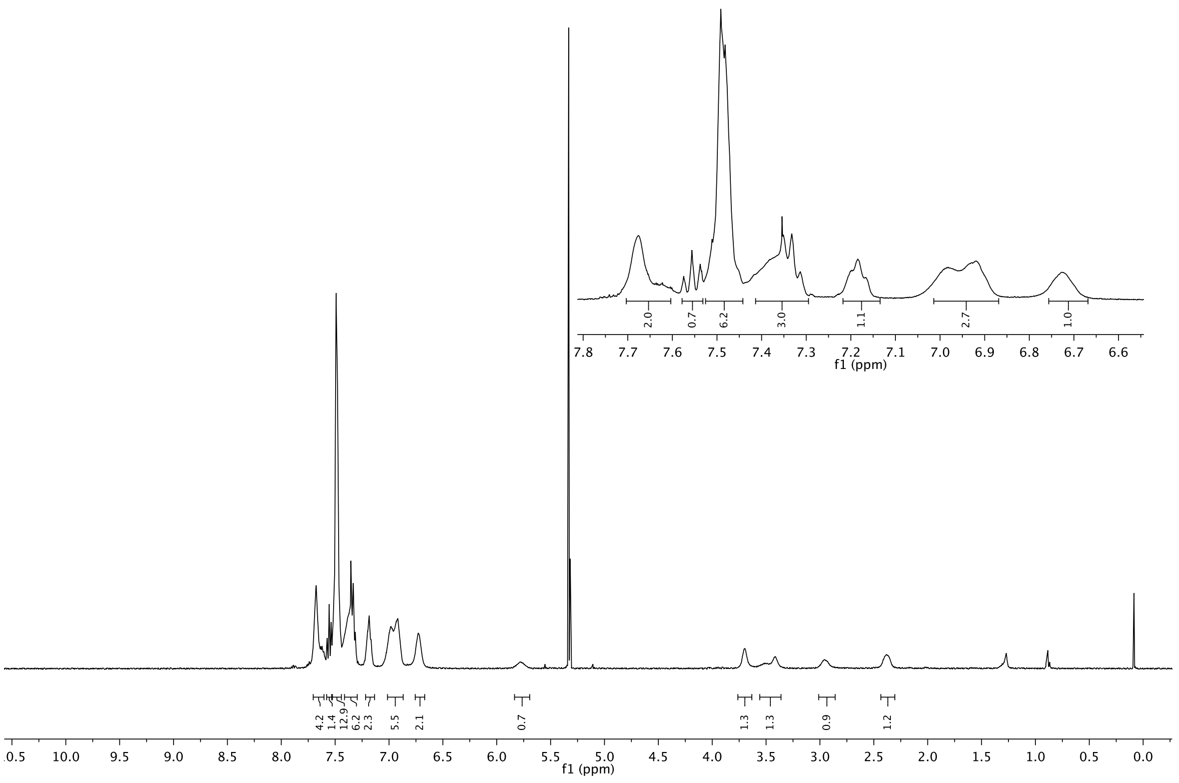

Figure S10. ^{1}H NMR (400 MHz, Methylene Chloride-d_2) [($^{Ph}DPB^{Ph}$)Pd(C_3H_5)]SbF_6 **10.**

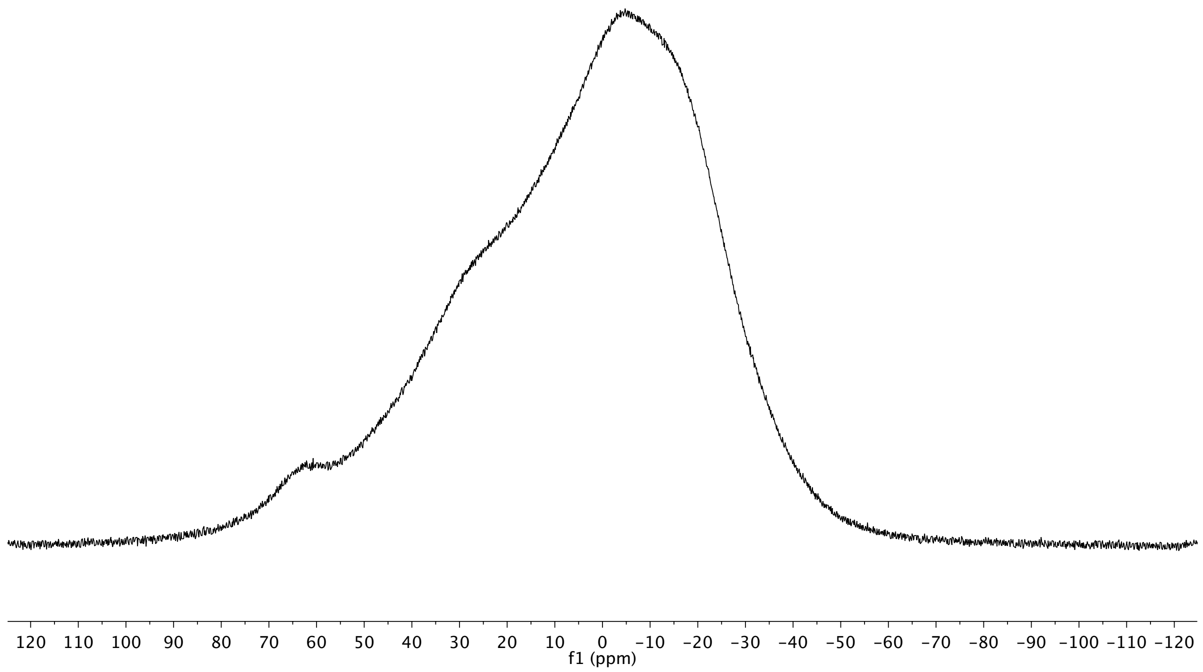

Figure S11. ^{11}B NMR (128 MHz, Methylene Chloride-d$_2$) [(^{Ph}DPB^{Ph})Pd(C_3H_5)]SbF_6 **10.**

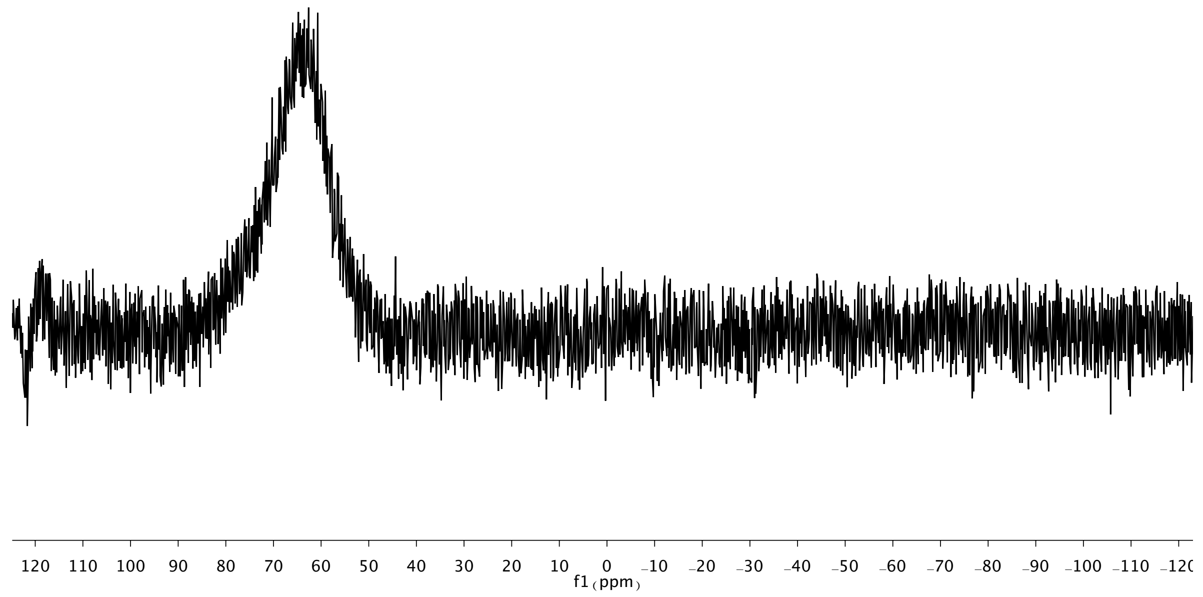

Figure S12. ^{11}B NMR (128 MHz, Methylene Chloride-d$_2$) [(^{Ph}DPB^{Ph})Pd(C_3H_5)]SbF_6 **10** after background subtraction.

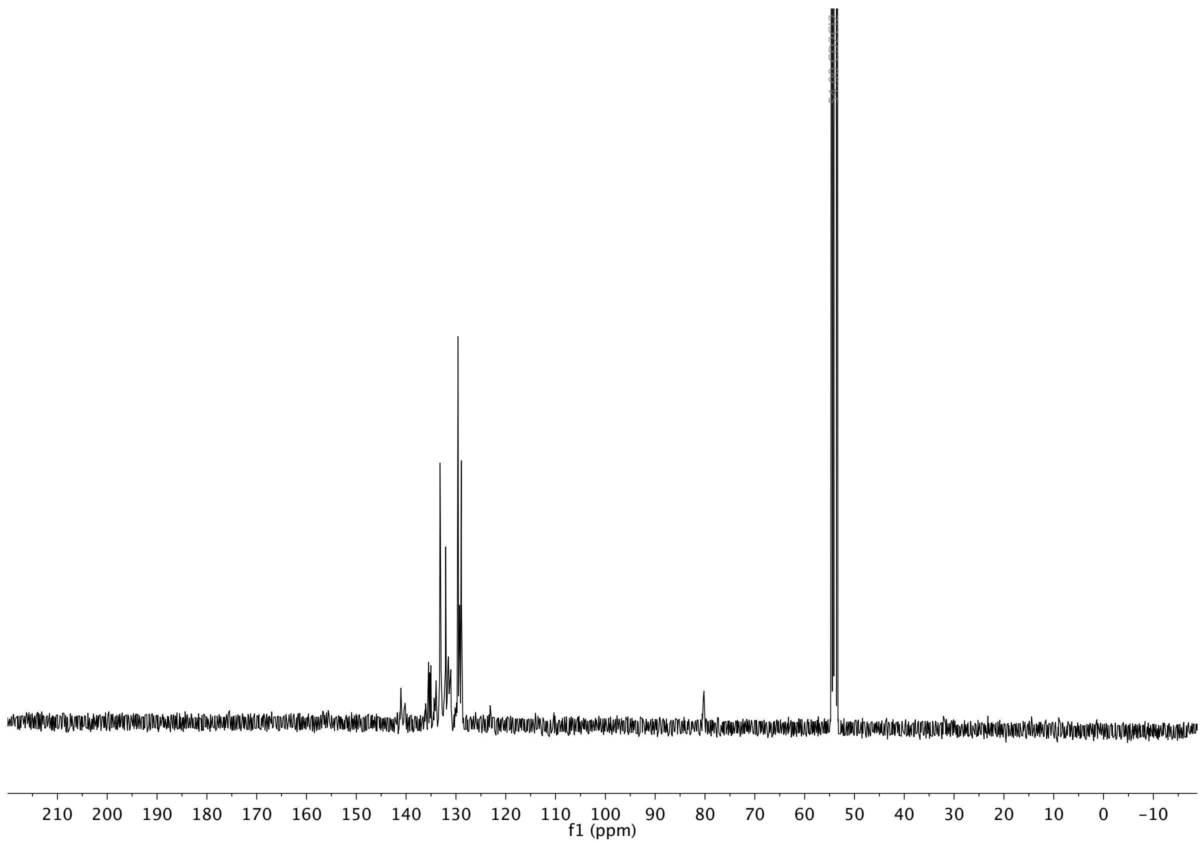

Figure S13. ^{13}C NMR (101 MHz, Methylene Chloride-d_2) [(^{Ph}DPB^{Ph})Pd(C_3H_5)]SbF_6 **10.**

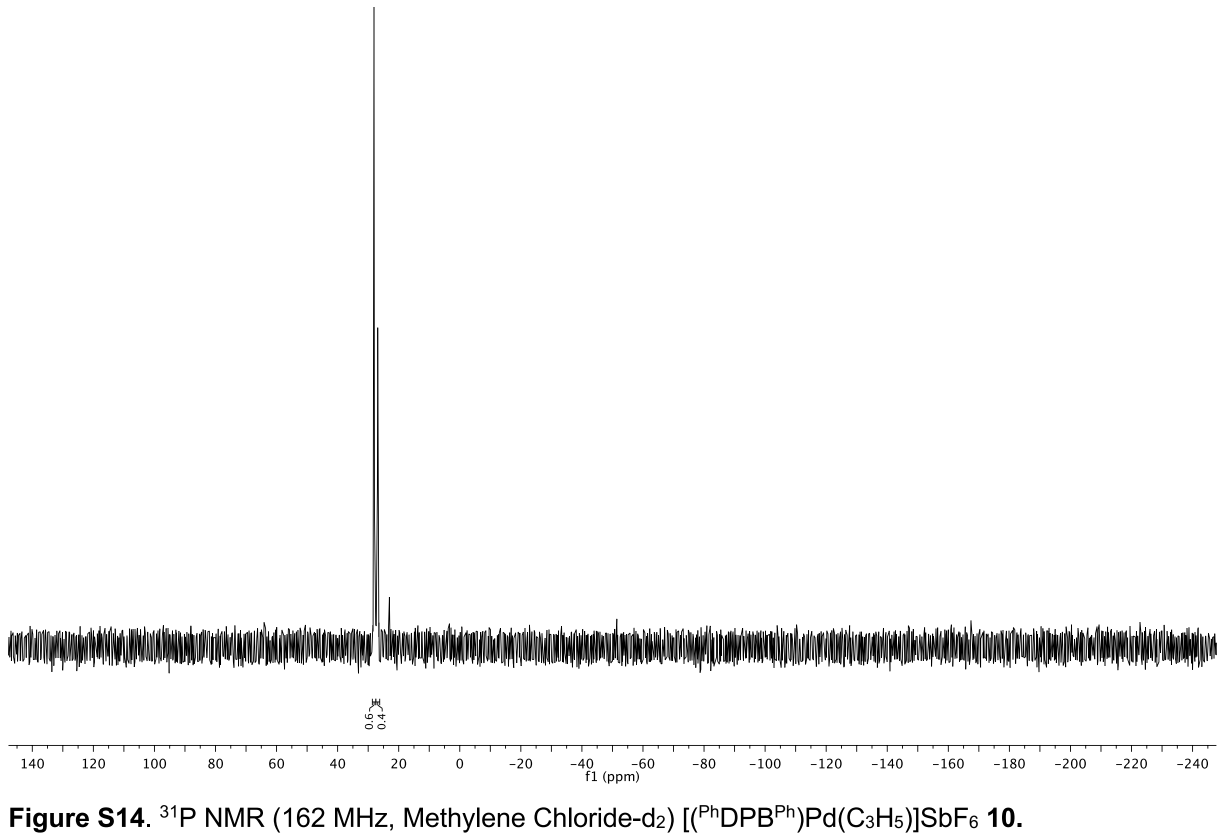

Figure S14. ^{31}P NMR (162 MHz, Methylene Chloride-d_2) [(^{Ph}DPB^{Ph})Pd(C_3H_5)]SbF_6 **10.**

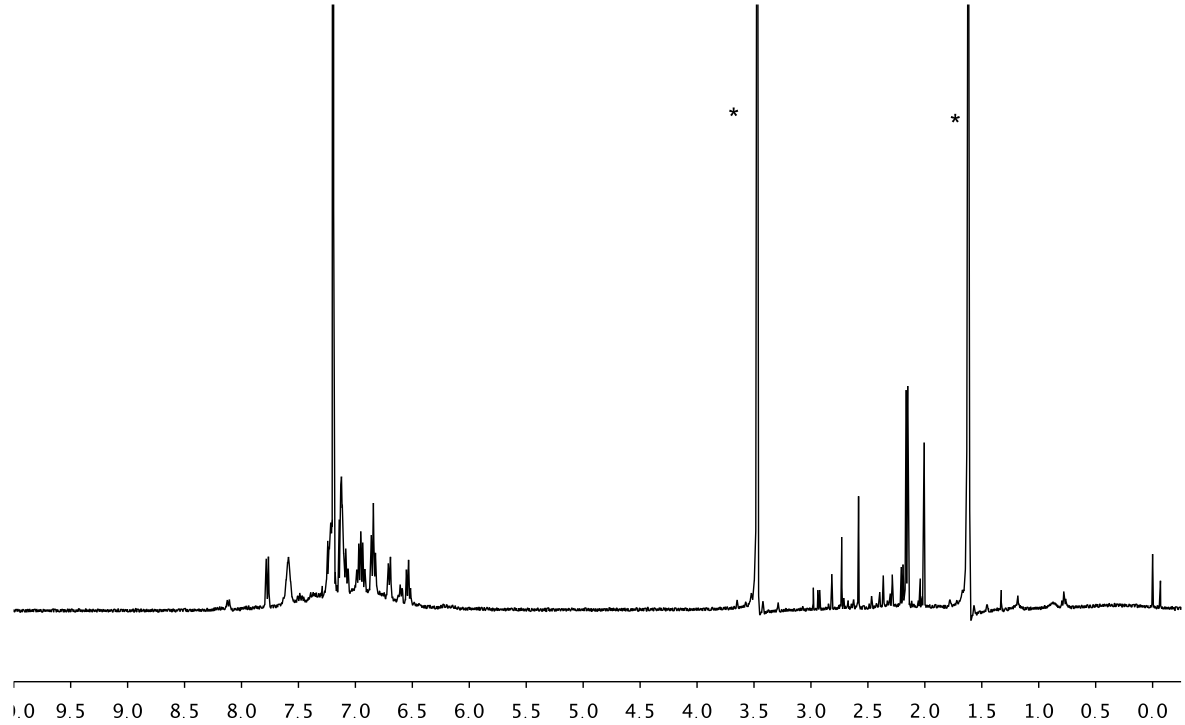

Figure S15. ^{1}H NMR (400 MHz, THF-d$_8$) [(^{Ph}DPB^{Ph})Pd] **6.** THF (*).

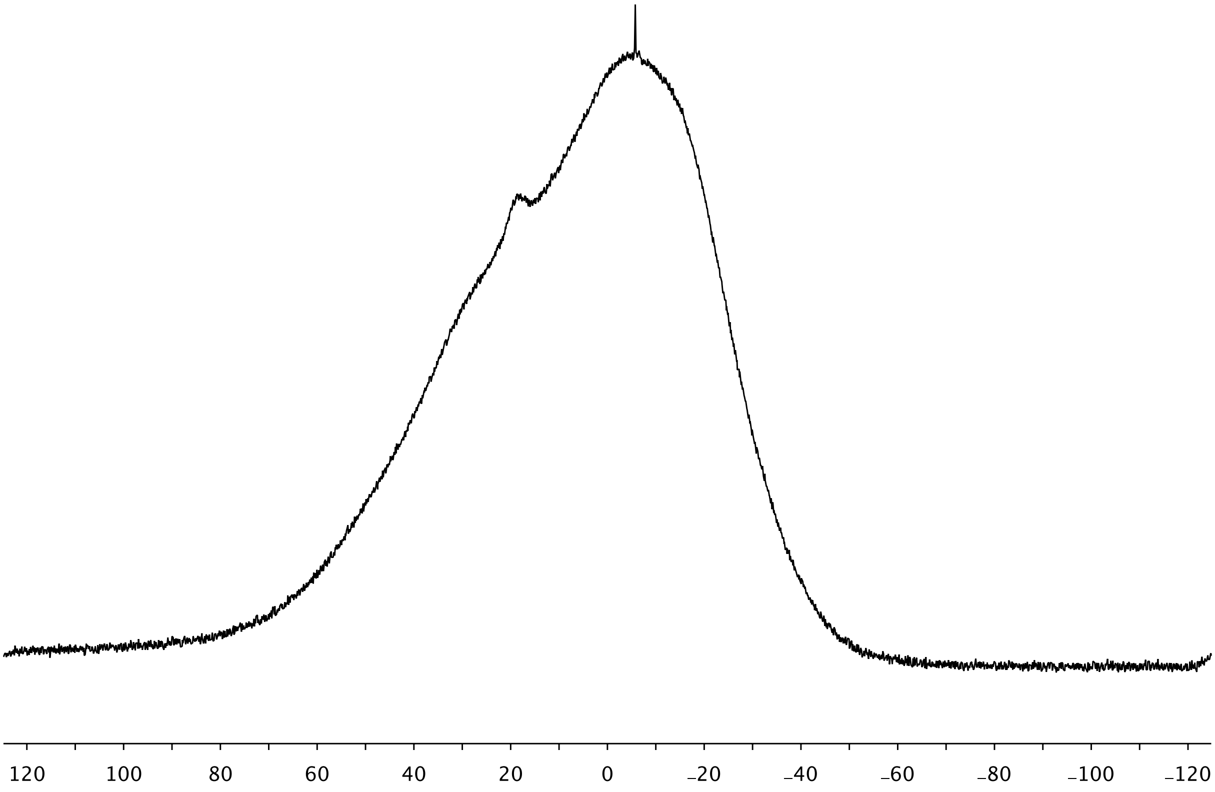

Figure S16. ^{11}B NMR (128 MHz, THF-d$_8$) [(^{Ph}DPB^{Ph})Pd] **$$6.**

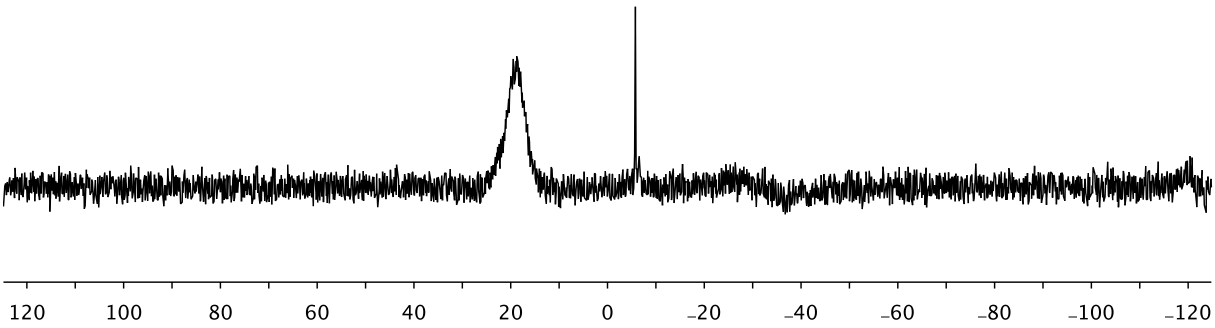

Figure S17. ^{11}B NMR (128 MHz, THF-d$_8$) [(^{Ph}DPB^{Ph})Pd] **6** after background subtraction.

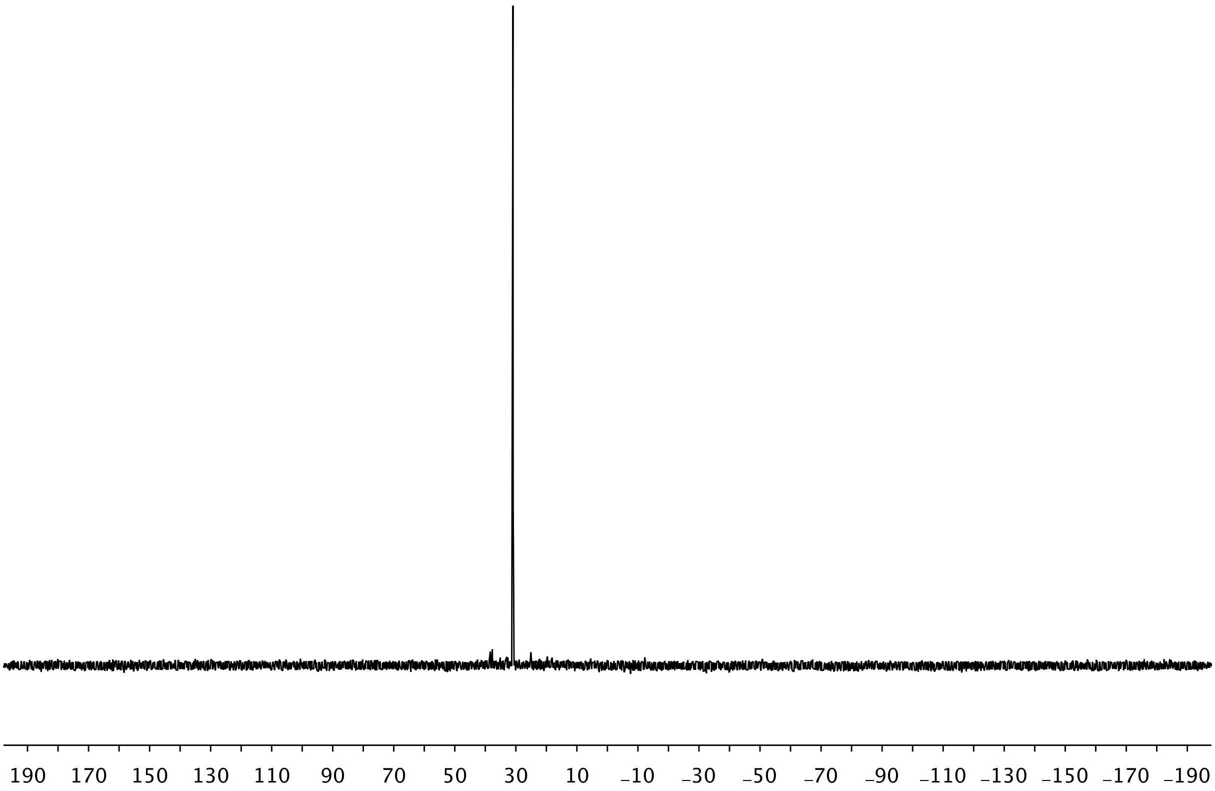

Figure S18. ^{31}P NMR (162 MHz, THF-d$_8$) [(^{Ph}DPB^{Ph})Pd] **6**.

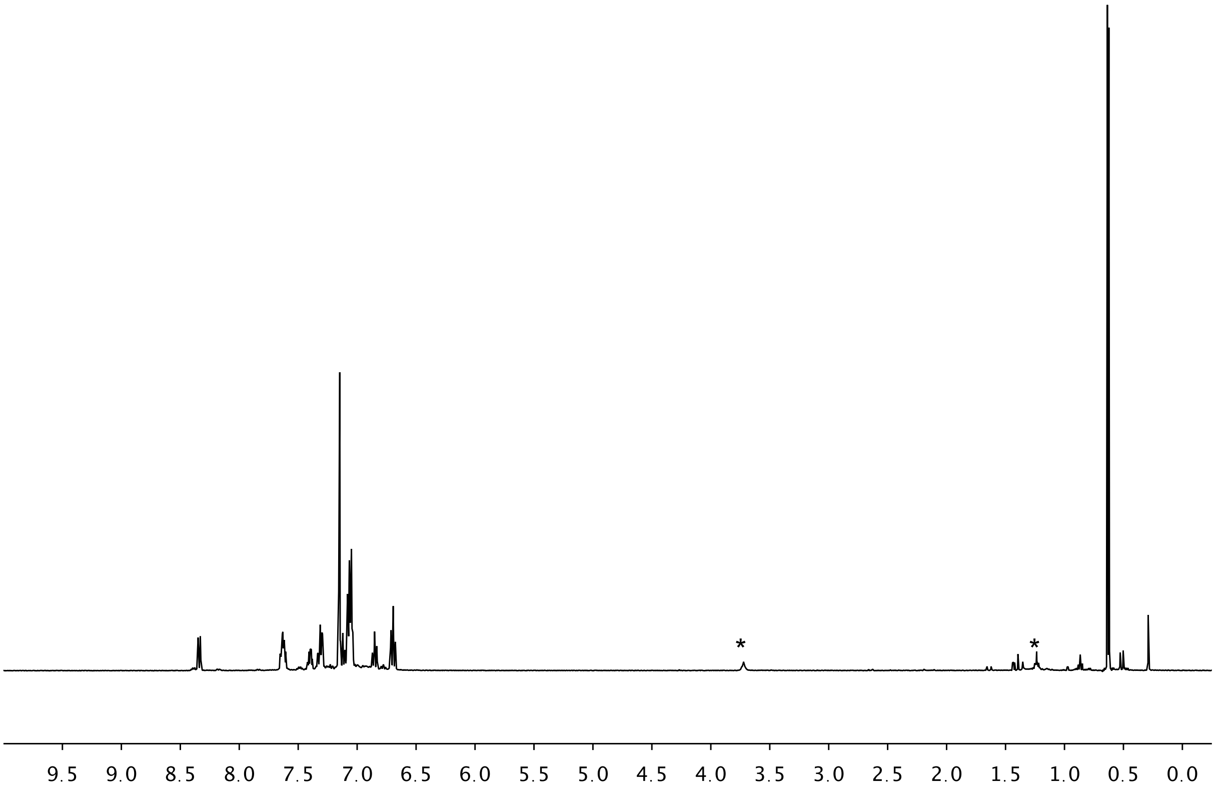

Figure S19. ^{1}H NMR (400 MHz, benzene-d$_6$) [(^{Ph}DPB^{Ph})Pd(PMe$_3$)] **11.** THF (*).

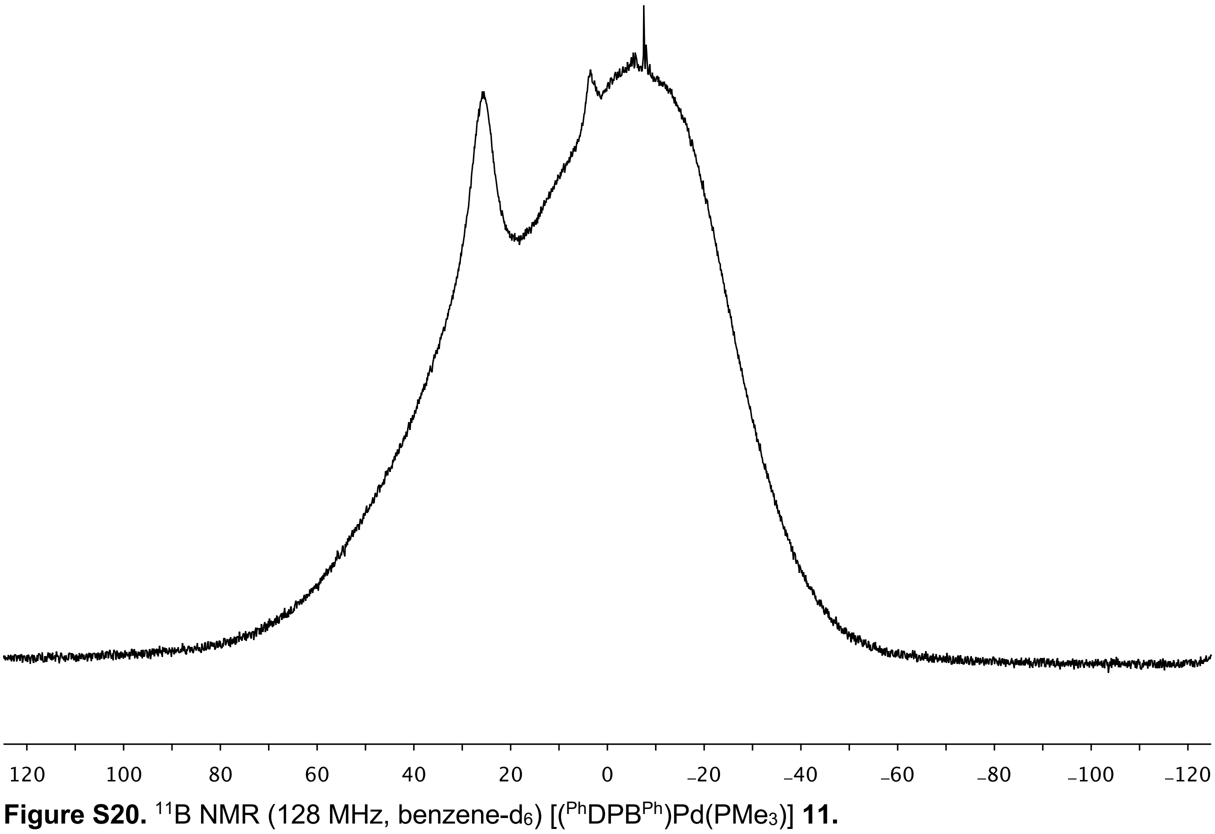

Figure S20. ^{11}B NMR (128 MHz, benzene-d$_6$) [(^{Ph}DPB^{Ph})Pd(PMe$_3$)] **11.**

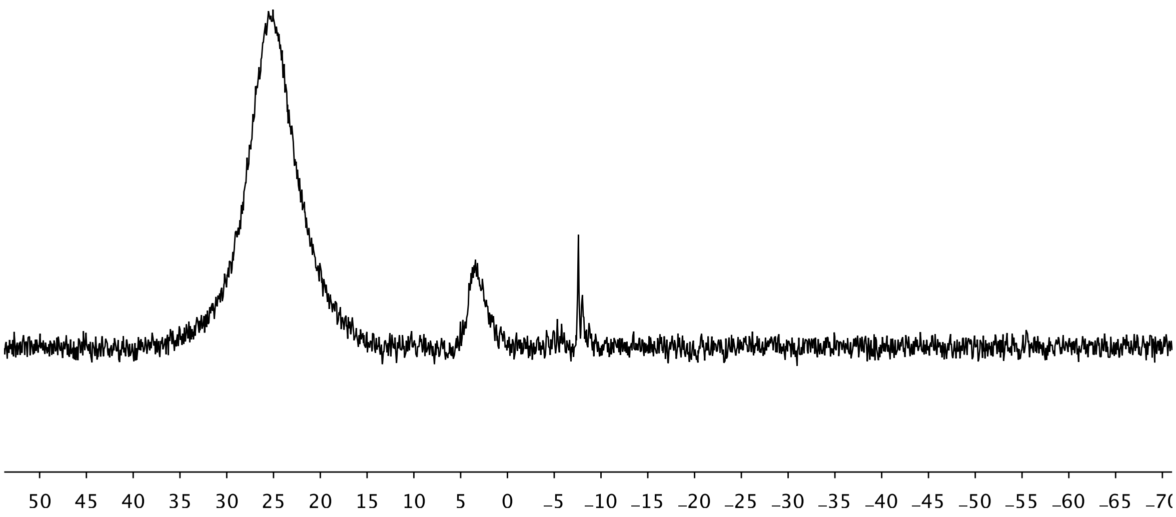

Figure S21. ^{11}B NMR (128 MHz, benzene-d_6) [(^{Ph}DPB^{Ph})Pd(PMe_3)] **11** after background subtraction.

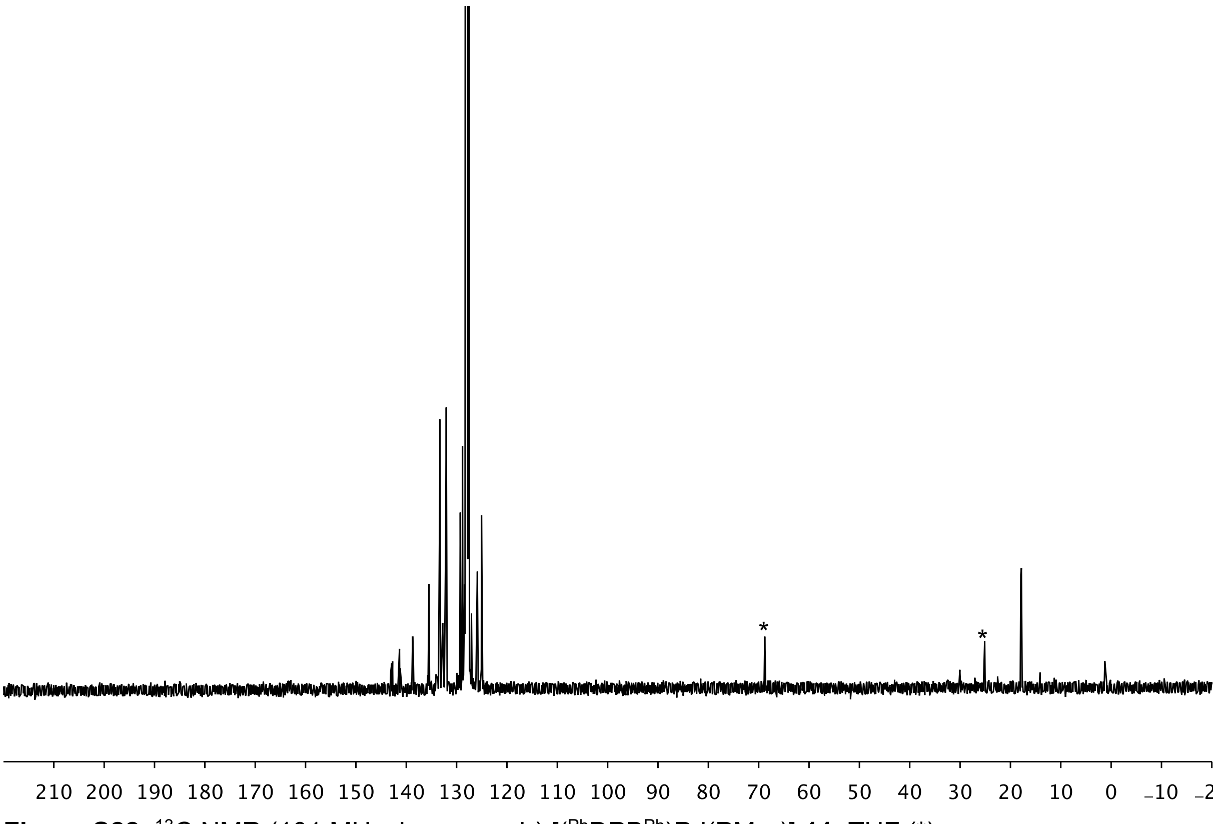

Figure S22. ^{13}C NMR (101 MHz, benzene-d_6) [(^{Ph}DPB^{Ph})Pd(PMe_3)] **11**. THF (*).

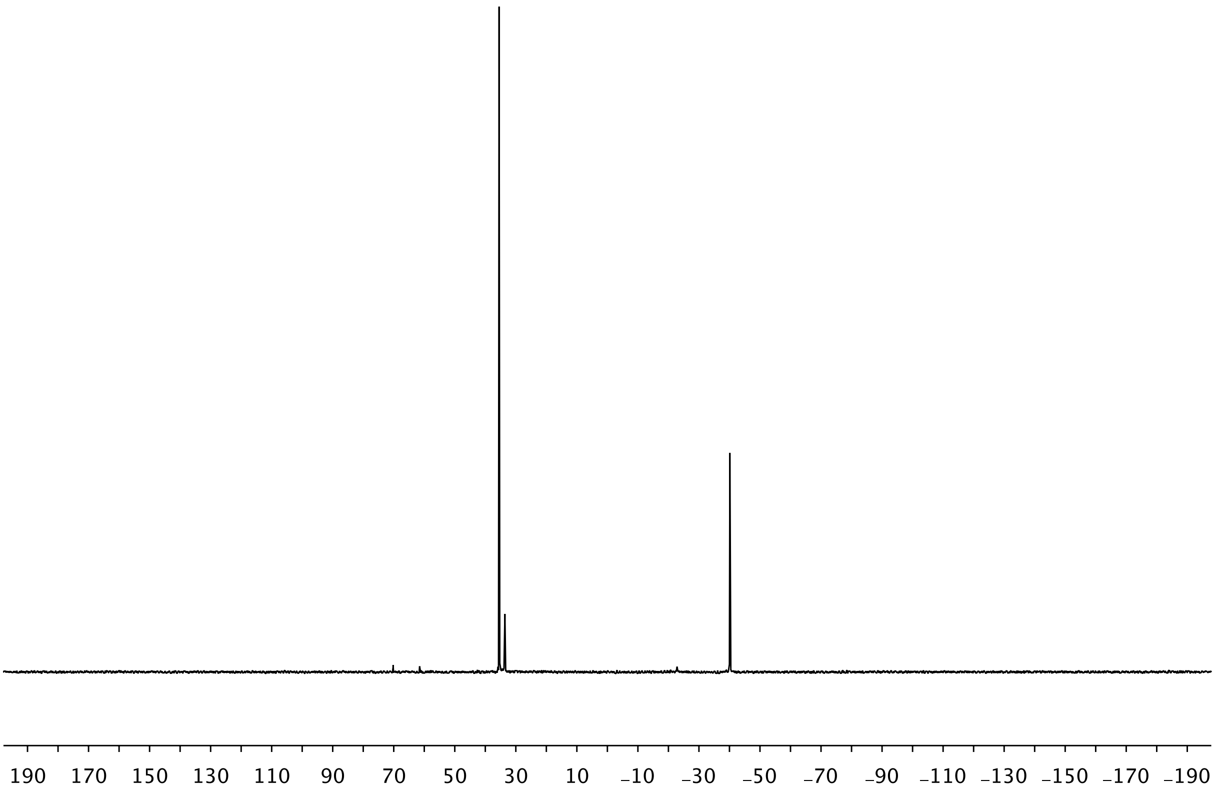

Figure S23. ^{31}P NMR (162 MHz, benzene-d_6) [(^{Ph}DPB^{Ph})Pd(PMe$_3$)] **11**.

5 Geometries of Stationary Points

1

Energy = -2702.8309355480

Pd	-0.0508214	-0.4014561	0.5430944
P	-0.2779472	1.8458160	1.0724915
P	0.3212187	-1.3073183	-1.5830009
C	2.0805921	-0.9195481	-1.9791247
C	-1.3502567	2.4208235	2.4839180
N	-1.8657166	-1.5697980	1.3561605
C	2.7683259	-0.1546020	-0.9945665
C	-0.6607776	-0.4315465	-2.8894568
C	1.4016917	2.4674697	1.5010625
C	2.4446382	1.5662477	1.1670406
C	1.8368256	-0.8810074	2.9029506
H	1.6355547	0.1455886	3.2518904
C	-0.2696484	-4.8781062	-3.7109378
H	-0.4276816	-5.1913718	-4.7567094
C	-0.8575387	2.9365259	-0.3120858
C	-2.0071980	-0.8079593	-3.1114722
H	-2.4386076	-1.6584623	-2.5562291
C	2.6868275	-1.2977091	-3.1959980
H	2.1285870	-1.9055157	-3.9274181
C	0.0824156	-3.0905838	-2.0748229
C	1.6432102	3.6892041	2.1660831
H	0.8038779	4.3574526	2.4231834
C	4.0791375	0.2653871	-1.3434805
H	4.6525025	0.8901829	-0.6406714
C	-0.1229645	-3.5137169	-3.4084122
H	-0.1843809	-2.7701676	-4.2199981
C	-2.7978767	-0.1235207	-4.0478575
H	-3.8414201	-0.4394390	-4.2165592
C	3.9954927	-0.8883211	-3.4886169
H	4.4689322	-1.1784172	-4.4416508
C	0.1193590	-4.0650476	-1.0532436
H	0.2654887	-3.7444418	-0.0084363
C	-3.1254509	-1.3687591	0.9119235
H	-3.2456821	-0.5867437	0.1433807
C	2.9534649	4.0441956	2.5186948
H	3.1497235	4.9958069	3.0406985
C	2.4852062	-3.4821201	2.0877343
H	2.7669684	-4.4987990	1.7636525
B	2.1303332	0.1328218	0.4714352
C	3.7524619	1.9447314	1.5690246
H	4.5945466	1.2519561	1.4022693
C	2.4757025	-2.4314783	1.1571956
H	2.7593131	-2.6419783	0.1118939
C	4.0078542	3.1598764	2.2256315
H	5.0385089	3.4127312	2.5284268
C	2.1497413	-3.2397545	3.4341697
H	2.1629142	-4.0630887	4.1687526
C	-2.7592134	4.0548561	3.6383712
H	-3.2541543	5.0406028	3.6536282
C	4.6852015	-0.0897814	-2.5588798
H	5.7091374	0.2563901	-2.7817409
C	-0.1353902	0.6612602	-3.6122209
H	0.9071171	0.9759142	-3.4475075
C	-0.0251641	-5.4296949	-1.3567249
H	0.0066164	-6.1766163	-0.5458116
C	-0.9317283	1.3487662	-4.5444673
H	-0.5023079	2.1980549	-5.1011929
C	-1.6684963	-2.5201778	2.2980809
H	-0.6273935	-2.6553950	2.6409701
C	-2.2618370	0.9577491	-4.7706894
H	-2.8813110	1.4946777	-5.5082297
C	-2.8944807	3.1816652	4.7317821
H	-3.4969801	3.4788561	5.6063959
C	2.1394707	-1.0937205	1.5223953
C	-0.0006306	3.8579583	-0.9510734
H	1.0422137	3.9627379	-0.6111293
C	-2.2636877	1.9253571	4.7014881
H	-2.3707458	1.2316155	5.5520638
C	-1.5040557	1.5458364	3.5826010
H	-1.0220947	0.5538306	3.5496116
C	-2.7116397	-3.2963789	2.8252696
H	-2.4919350	-4.0559162	3.5923182
C	-0.2184469	-5.8401577	-2.6867573
H	-0.3364705	-6.9103834	-2.9258093
C	-2.1882900	2.8118250	-0.7738996
H	-2.8691359	2.0878574	-0.2941378
C	-2.6572381	3.6063886	-1.8301842
H	-3.6995339	3.4993439	-2.1737563
C	-1.9923873	3.6788568	2.5214349
H	-1.8974913	4.3705342	1.6686277
C	1.8296774	-1.9307434	3.8382570
H	1.5924524	-1.7214527	4.8956716
C	-4.2274944	-2.0996238	1.3807972
H	-5.2330040	-1.8921149	0.9812760
C	-1.7990028	4.5301904	-2.4544882
H	-2.1670964	5.1541767	-3.2860610
C	-4.0192916	-3.0853683	2.3586437
H	-4.8620265	-3.6786755	2.7503918
C	-0.4704320	4.6482299	-2.0157004
H	0.2112538	5.3666301	-2.5016239

3

Energy = -2469.134047194

Pd	0.0500259	-0.2173041	0.3769459
P	0.6932825	1.2501874	2.0437288
P	-0.3312051	-1.0166001	-1.7843418
C	1.1980684	-1.5736450	-2.7441335
H	0.8804230	-1.7422045	-3.8006079
C	2.3826623	2.0744321	1.7658639
H	2.6976639	2.4981294	2.7508039
C	-3.9030119	0.2490579	-0.9492004
H	-3.7128348	0.7140878	-1.9320274
C	-1.5259508	2.4837811	0.9434528
C	0.7163721	0.7878327	3.8811568
H	0.8922117	1.7415058	4.4352190
C	-0.5454423	2.6110596	1.9612379
C	-1.2564105	1.5972740	-1.6864509

C	1.8466935	-0.1976388	4.2502464
H	2.8409375	0.2446790	4.0232443
H	1.7509711	-1.1149781	3.6216558
C	-5.1173717	-0.4337618	-0.7487157
H	-5.8542422	-0.4956256	-1.5684591
C	-0.6937241	-0.1612167	5.7934688
H	-0.5866788	0.7601404	6.4138825
H	-1.6886457	-0.5937010	6.0443149
C	-1.7447206	-2.7972660	-3.6228891
H	-0.8297787	-3.3973343	-3.8403183
H	-1.7587413	-1.9525784	-4.3479551
C	3.6695946	3.8724593	0.4873839
H	3.5686301	4.6740620	-0.2790752
H	4.0157742	4.3749148	1.4225891
C	-0.8397265	0.5706501	-2.5905831
C	3.0322171	-3.3447158	-2.9392829
H	3.4241948	-4.2838994	-2.4873220
H	2.7667309	-3.5918261	-3.9951730
C	-2.5539922	4.5604909	1.8033413
H	-3.3590243	5.3128618	1.7428803
C	-0.9729029	2.0971145	-4.4955750
H	-0.8656761	2.2857421	-5.5770518
C	2.2896351	-0.4807593	-2.7309910
H	1.8984856	0.4616131	-3.1710494
H	2.5451994	-0.2472527	-1.6700855
C	4.1118678	-2.2508006	-2.9313412
H	4.4663947	-2.0926465	-1.8849751
H	4.9994114	-2.5782907	-3.5192393
C	0.4289403	-1.1492891	6.1495867
H	0.2518942	-2.1144319	5.6174012
H	0.4146246	-1.3818858	7.2384726
C	-1.5528505	4.6788004	2.7852645
H	-1.5593715	5.5254714	3.4921252
C	-2.9854452	-3.6887431	-3.8407965
H	-3.9028684	-3.0614091	-3.7470197
H	-2.9821685	-4.0920387	-4.8792494
C	-2.9307038	0.3761966	0.0779187
C	-2.9874610	-4.3056709	-1.3751952
H	-2.9856800	-5.1518235	-0.6507427
H	-3.9027903	-3.7061832	-1.1607513
C	1.7598964	-2.9028481	-2.1897056
H	1.9911751	-2.7713217	-1.1053422
H	0.9968597	-3.7090725	-2.2530812
C	-1.6952568	-2.2671143	-2.1759688
H	-2.6052640	-1.6472822	-2.0060649
C	3.5574746	-0.9270582	-3.4830685
H	3.3192079	-1.0507169	-4.5668059
H	4.3291499	-0.1261291	-3.4227488
C	-3.2549289	-0.2195151	1.3261059
H	-2.5410073	-0.1231674	2.1606121
C	-0.7137366	0.8172337	-3.9728236
H	-0.3829939	0.0167395	-4.6566155
C	3.4347690	1.0388451	1.3041422
H	3.5312844	0.2094593	2.0373815
H	3.0662422	0.5739456	0.3587635
C	-4.4517266	-0.9228889	1.5315763
H	-4.6597100	-1.3800057	2.5143045
C	-1.4797886	2.8788791	-2.2451485
H	-1.7700861	3.7099468	-1.5809910
C	-1.3419455	3.1320797	-3.6222319
H	-1.5227122	4.1476160	-4.0143227
C	2.2935304	3.2271130	0.7403269
H	1.8845405	2.8261380	-0.2169152
H	1.5746675	3.9995250	1.0868954
C	-1.7474878	-3.4193723	-1.1467646
H	-0.8301183	-4.0482769	-1.2270949
H	-1.7569656	-2.9980122	-0.1168474
C	4.8118636	1.6846529	1.0597231
H	5.2160575	2.0691772	2.0265582
H	5.5304847	0.9110087	0.7050056
C	-2.5394721	3.4789188	0.9097113
H	-3.3561770	3.3884704	0.1726690
C	-3.0492525	-4.8364158	-2.8181267
H	-2.1921809	-5.5304131	-2.9931668
H	-3.9747137	-5.4367906	-2.9708328
C	-0.5557385	3.6968626	2.8674748
H	0.2199149	3.7815801	3.6492750
C	-5.3937280	-1.0334319	0.4913703
H	-6.3420092	-1.5741537	0.6517301
C	-0.6571236	0.2220452	4.3022719
H	-0.8708353	-0.6823577	3.6842002
H	-1.4592524	0.9578451	4.0774286
C	1.8008944	-0.5893589	5.7412423
H	2.6050868	-1.3290114	5.9568013
H	2.0284815	0.3111704	6.3608252
C	4.7217668	2.8401672	0.0498557
H	4.4408705	2.4314980	-0.9497826
H	5.7159298	3.3264950	-0.0749759
B	-1.5894235	1.2602789	-0.1284112

5

Energy = -2902.046852735

I	0.2564921	-2.7192928	-2.7986292
Pd	0.5136275	-0.7441608	-0.9502431
P	-1.5962510	0.2698514	-1.7576186
P	0.9247607	0.7522115	0.7737123
O	7.2034507	-2.7265577	-0.9356769
O	6.5048155	-4.0281999	0.6790761
N	6.3216184	-3.1696196	-0.1908678
C	-2.8345862	-1.0533567	-1.4199298
C	-3.8966108	-1.3561641	-2.2898316
H	-4.0127661	-0.8042604	-3.2358733
C	-4.8002268	-2.3859696	-1.9731686
H	-5.6254618	-2.6244698	-2.6642803
C	-4.6318434	-3.1126211	-0.7854034
H	-5.3303903	-3.9266581	-0.5289282
C	-3.5503350	-2.8248723	0.0664431
H	-3.4173148	-3.4277158	0.9799640
C	-2.6184286	-1.7960330	-0.2236048
C	-0.7675256	-2.7597524	1.5257940
C	-0.6358498	-4.0005270	0.8410573
H	-0.9402477	-4.0641381	-0.2163445

C	-0.0924375	-5.1317099	1.4661410
H	0.0091259	-6.0740663	0.9026918
C	0.3317249	-5.0614081	2.8065534
H	0.7599357	-5.9503493	3.2999444
C	0.2097542	-3.8518986	3.5128759
H	0.5355295	-3.7906965	4.5647066
C	-0.3277687	-2.7216192	2.8765404
H	-0.4345339	-1.7873957	3.4504866
C	-0.6525365	0.9518365	1.7138820
C	-0.9173444	2.1052341	2.4834789
H	-0.2199676	2.9572330	2.4641740
C	-2.0727177	2.1831726	3.2739002
H	-2.2737784	3.0895717	3.8682597
C	-2.9668632	1.0998539	3.2967556
H	-3.8754475	1.1425932	3.9208065
C	-2.7176338	-0.0293371	2.5026325
H	-3.4503295	-0.8552713	2.5076781
C	-1.5638797	-0.1381484	1.6826751
C	2.1809017	0.4042796	2.0906199
C	1.8146317	0.1599024	3.4305928
H	0.7546622	0.1789307	3.7251621
C	2.8001997	-0.0853939	4.4034674
H	2.4986976	-0.2711593	5.4476354
C	4.1584158	-0.0896183	4.0488551
H	4.9302274	-0.2847658	4.8117382
C	4.5309756	0.1587441	2.7153629
H	5.5948144	0.1574907	2.4273599
C	3.5514776	0.4030299	1.7420448
H	3.8590363	0.5922280	0.7015279
C	1.5331935	2.3909897	0.1577340
C	2.0123917	3.3879158	1.0389724
H	2.0279777	3.2074730	2.1260139
C	2.5087097	4.6015752	0.5366086
H	2.8745056	5.3714966	1.2358999
C	2.5587008	4.8263332	-0.8506798
H	2.9613509	5.7748848	-1.2428078
C	2.1142775	3.8294786	-1.7350211
H	2.1707120	3.9877488	-2.8246191
C	1.6042760	2.6197508	-1.2327334
H	1.2686251	1.8306531	-1.9277020
C	-2.4081124	1.7988125	-1.0618198
C	-3.6030272	1.7428863	-0.3148153
H	-4.0755306	0.7712184	-0.1054624
C	-4.2051785	2.9231471	0.1562759
H	-5.1421020	2.8602328	0.7342966
C	-3.6237123	4.1730105	-0.1107053
H	-4.0999645	5.0972365	0.2568267
C	-2.4336362	4.2381097	-0.8565513
H	-1.9701653	5.2133895	-1.0797568
C	-1.8322890	3.0617732	-1.3291905
H	-0.9134566	3.1353047	-1.9309367
C	-1.6559870	0.7210294	-3.5595687
C	-2.7964180	1.3638064	-4.0984745
H	-3.6584577	1.5966617	-3.4526952
C	-2.8337409	1.7333573	-5.4526999
H	-3.7333353	2.2255169	-5.8582951
C	-1.7258630	1.4878351	-6.2830254
H	-1.7541553	1.7836531	-7.3449544
C	-0.5812066	0.8725394	-5.7510758
H	0.2948815	0.6802123	-6.3918937
C	-0.5462009	0.4881866	-4.3992490
H	0.3461436	-0.0172706	-3.9985940
C	2.3586291	-1.5941898	-0.6336695
C	3.4097408	-1.1510768	-1.4732639
H	3.2203218	-0.4051419	-2.2627397
C	4.7089863	-1.6615985	-1.3347314
H	5.5347463	-1.3304106	-1.9809815
C	4.9550001	-2.6304087	-0.3480532
C	3.9304207	-3.0937480	0.4918469
H	4.1600535	-3.8571337	1.2488448
C	2.6376413	-2.5678184	0.3475944
H	1.8400013	-2.9334235	1.0105159
B	-1.4605459	-1.5129552	0.8511401

6

Energy = -2454.708409366

Thermal correction to Gibbs free Energy = 1368.48 KJ/mol

Pd	-0.5342745	-0.3394008	0.5251939
B	0.1516063	-0.6718444	-1.5951851
P	-1.9664349	1.2337070	-0.3332065
P	1.6423415	-0.8538916	1.1800239
C	2.2329262	-2.5139371	1.7536183
C	3.3435698	-3.1865812	1.2019864
H	3.9175178	-2.7220022	0.3838576
C	3.7202498	-4.4530037	1.6842319
H	4.5889956	-4.9681403	1.2412066
C	2.9943399	-5.0600354	2.7229843
H	3.2903377	-6.0537656	3.0983379
C	1.8825628	-4.3983452	3.2752313
H	1.3012688	-4.8727124	4.0833423
C	1.5005023	-3.1381623	2.7888109
H	0.6151777	-2.6331911	3.2124881
C	2.3845657	0.2927996	2.4383299
C	1.9066189	1.6222411	2.4773568
H	1.0992798	1.9272441	1.7879839
C	2.4442597	2.5451615	3.3876653
H	2.0627692	3.5794780	3.4026495
C	3.4547867	2.1489946	4.2824740
H	3.8711067	2.8720380	5.0037618
C	3.9261111	0.8258468	4.2586145
H	4.7148514	0.5066135	4.9605930
C	3.3968054	-0.0991221	3.3408620
H	3.7739604	-1.1346772	3.3335760
C	-3.7956654	1.1699462	-0.0294119
C	-4.6368782	2.3025963	-0.1055554
H	-4.2076603	3.2962235	-0.3145203
C	-6.0213339	2.1734326	0.0981577
H	-6.6675681	3.0650278	0.0368948
C	-6.5811778	0.9148968	0.3803001
H	-7.6676674	0.8168026	0.5414733
C	-5.7503226	-0.2158182	0.4652118

H	-6.1812975	-1.2042049	0.6957809
C	-4.3653215	-0.0880793	0.2676687
H	-3.7092039	-0.9721938	0.3476933
C	-1.5215207	2.9712588	0.1256973
C	-1.8337206	3.4472416	1.4212049
H	-2.3959082	2.8069842	2.1222308
C	-1.4370466	4.7320467	1.8238898
H	-1.6961436	5.0911070	2.8339577
C	-0.7099439	5.5564035	0.9448391
H	-0.3962975	6.5646892	1.2623467
C	-0.3847895	5.0859174	-0.3383642
H	0.1875683	5.7233054	-1.0329197
C	-0.7876565	3.8021746	-0.7481527
H	-0.5271057	3.4425416	-1.7564273
C	-0.2842710	-2.2144298	-1.4951218
C	0.6524259	-3.2666002	-1.3043406
H	1.7245662	-3.0233588	-1.2214434
C	0.2580589	-4.6126747	-1.2401146
H	1.0171644	-5.3997515	-1.0934610
C	-1.0990589	-4.9577874	-1.3655960
H	-1.4118861	-6.0144841	-1.3143852
C	-2.0524370	-3.9437821	-1.5736652
H	-3.1181170	-4.2040229	-1.6934523
C	-1.6474634	-2.6025425	-1.6473968
H	-2.4054992	-1.8254028	-1.8444721
C	-0.7785484	0.2003130	-2.6021933
C	-0.6824250	0.0059904	-4.0059460
H	0.0212519	-0.7475411	-4.3997662
C	-1.4858684	0.7139319	-4.9135896
H	-1.3827464	0.5295887	-5.9965798
C	-2.4346920	1.6419944	-4.4462148
H	-3.0724451	2.1962728	-5.1549282
C	-2.5821905	1.8342625	-3.0656530
H	-3.3491911	2.5326443	-2.6898634
C	-1.7647366	1.1206606	-2.1610203
C	1.7405633	-0.3180411	-1.5603406
C	2.4195114	0.1222129	-2.7249029
H	1.8488442	0.2734139	-3.6552851
C	3.7984263	0.3926504	-2.7398216
H	4.2779402	0.7352672	-3.6726787
C	4.5600217	0.2496822	-1.5686266
H	5.6387621	0.4785959	-1.5670997
C	3.9226509	-0.1603058	-0.3868267
H	4.5007972	-0.2256378	0.5504047
C	2.5414510	-0.4487296	-0.3844029

7

Energy = -3374.974001473

Pd	-1.22920	-1.15876	0.50883
B	-0.04443	-1.13899	-1.82786
P	-1.75059	0.96588	-0.34162
P	0.93310	-0.76977	1.20963
Cl	-3.43362	-1.65866	-0.24850
Cl	-1.16041	-3.23587	1.62772
C	2.04772	-2.16809	1.67441
C	3.15686	-2.52522	0.88073
H	3.37394	-1.97443	-0.04649
C	4.00379	-3.57506	1.27782
H	4.86943	-3.84177	0.64910
C	3.74820	-4.27696	2.46593
H	4.41098	-5.10262	2.77440
C	2.64406	-3.92309	3.26192
H	2.43492	-4.47234	4.19467
C	1.79455	-2.87812	2.87155
H	0.92919	-2.61675	3.49804
C	1.00344	0.25680	2.74745
C	-0.20000	0.67549	3.35315
H	-1.16358	0.41246	2.88440
C	-0.17889	1.40083	4.55705
H	-1.12855	1.71140	5.02286
C	1.04620	1.71767	5.16686
H	1.06376	2.28593	6.11167
C	2.25009	1.28593	4.58176
H	3.21404	1.51124	5.06723
C	2.23025	0.54628	3.38867
H	3.17753	0.16614	2.97383
C	-3.24112	1.76629	0.41676
C	-3.56208	3.09884	0.06286
H	-2.92998	3.65348	-0.64899
C	-4.67943	3.73494	0.62824
H	-4.92366	4.76925	0.33423
C	-5.47411	3.05938	1.57053
H	-6.34739	3.56152	2.01916
C	-5.14795	1.74425	1.94093
H	-5.76405	1.20660	2.68023
C	-4.04163	1.09552	1.36602
H	-3.81646	0.05107	1.62967
C	-0.56865	2.40153	-0.30126
C	-0.32486	3.06806	0.92093
H	-0.82148	2.73238	1.84403
C	0.53192	4.17811	0.97027
H	0.70960	4.68498	1.93299
C	1.15239	4.64398	-0.20218
H	1.82180	5.51953	-0.16453
C	0.90963	3.99203	-1.42221
H	1.38555	4.35387	-2.34863
C	0.05615	2.87649	-1.47388
H	-0.13098	2.37867	-2.43715
C	0.09730	-2.69700	-2.03610
C	1.37237	-3.31447	-2.13730
H	2.28097	-2.70080	-2.02055
C	1.50966	-4.68282	-2.41846
H	2.51392	-5.13220	-2.49952
C	0.36462	-5.47931	-2.59109
H	0.46702	-6.55791	-2.79999
C	-0.91252	-4.89626	-2.49149
H	-1.81454	-5.51859	-2.61440
C	-1.04263	-3.52683	-2.22620
H	-2.04779	-3.08706	-2.11820
C	-1.18537	-0.37353	-2.66475
C	-1.35369	-0.67955	-4.03665
H	-0.74769	-1.48459	-4.48536

C	-2.29159	-0.00018	-4.83568
H	-2.40350	-0.27099	-5.89912
C	-3.10435	0.99778	-4.27545
H	-3.86052	1.51401	-4.88981
C	-2.96799	1.32158	-2.91400
H	-3.62950	2.07863	-2.46240
C	-2.01297	0.65057	-2.13154
C	1.27547	-0.26848	-1.49648
C	1.99560	0.23661	-2.60961
H	1.62483	0.02442	-3.62744
C	3.15684	1.01019	-2.45830
H	3.68978	1.38172	-3.34991
C	3.62457	1.33067	-1.17237
H	4.51823	1.96293	-1.04186
C	2.94328	0.83974	-0.04881
H	3.30944	1.09555	0.95757
C	1.79479	0.03581	-0.20799

8

Energy = -7603.032841220

Pd	-1.25971	-1.19233	0.52148
B	-0.05509	-1.15371	-1.84353
P	-1.75961	0.95025	-0.33634
P	0.91897	-0.79622	1.21813
Br	-3.59229	-1.75014	-0.25281
Br	-1.22507	-3.36818	1.73858
C	2.06957	-2.16505	1.69116
C	3.16365	-2.52751	0.87890
H	3.35139	-1.99545	-0.06523
C	4.03512	-3.55587	1.27922
H	4.88812	-3.82601	0.63491
C	3.82114	-4.23082	2.49114
H	4.50340	-5.03937	2.80238
C	2.73480	-3.86997	3.30792
H	2.55907	-4.39576	4.26082
C	1.86049	-2.84684	2.91326
H	1.01178	-2.57758	3.55928
C	0.97615	0.24059	2.75127
C	-0.23381	0.64165	3.35578
H	-1.19240	0.35693	2.88956
C	-0.22553	1.37511	4.55482
H	-1.18055	1.67165	5.01872
C	0.99389	1.71718	5.16220
H	1.00190	2.29150	6.10348
C	2.20483	1.30242	4.57932
H	3.16487	1.54707	5.06321
C	2.19770	0.55528	3.39076
H	3.15154	0.18873	2.97907
C	-3.23432	1.78149	0.42267
C	-3.56043	3.10218	0.03114
H	-2.94399	3.63051	-0.71366
C	-4.66141	3.76167	0.60182
H	-4.91091	4.78548	0.27707
C	-5.43250	3.12359	1.58887
H	-6.29294	3.64427	2.04114
C	-5.09749	1.82331	2.00100
H	-5.69268	1.31565	2.77762
C	-4.00856	1.15119	1.41956
H	-3.77408	0.11973	1.72358
C	-0.56625	2.37953	-0.29910
C	-0.31995	3.04575	0.92282
H	-0.81532	2.71043	1.84683
C	0.53785	4.15503	0.97124
H	0.71734	4.66126	1.93396
C	1.15625	4.62142	-0.20207
H	1.82599	5.49676	-0.16533
C	0.91049	3.97027	-1.42190
H	1.38433	4.33260	-2.34921
C	0.05645	2.85503	-1.47256
H	-0.13274	2.35854	-2.43608
C	0.08936	-2.70836	-2.07017
C	1.36537	-3.32970	-2.13030
H	2.27017	-2.72110	-1.96932
C	1.51014	-4.69396	-2.42569
H	2.51554	-5.14557	-2.47229
C	0.37096	-5.48367	-2.65806
H	0.47864	-6.55927	-2.87928
C	-0.90725	-4.89710	-2.60459
H	-1.80531	-5.51322	-2.77739
C	-1.04314	-3.53162	-2.32276
C	-1.19495	-0.37713	-2.67055
C	-1.35924	-0.66917	-4.04632
H	-0.75130	-1.46905	-4.50152
C	-2.29441	0.01753	-4.84215
H	-2.40241	-0.24284	-5.90858
C	-3.10896	1.00938	-4.27436
H	-3.86421	1.53102	-4.88525
C	-2.97524	1.32102	-2.90982
H	-3.63887	2.07331	-2.45409
C	-2.02248	0.64383	-2.12971
C	1.26114	-0.28714	-1.49325
C	1.98605	0.21940	-2.60320
H	1.61972	0.00785	-3.62277
C	3.14621	0.99309	-2.44710
H	3.68255	1.36535	-3.33634
C	3.60829	1.31233	-1.15885
H	4.50110	1.94476	-1.02356
C	2.92294	0.81913	-0.03912
H	3.28644	1.07299	0.96851
C	1.77496	0.01464	-0.20233
H	-1.98623	-3.11332	-2.29779

9

Energy = -2914.630170151

Pd	-1.0936807	-1.0414433	0.4295341
B	0.0257521	-1.0354924	-1.8659909
C	0.0778439	-2.6040145	-1.9934096
P	-1.6662339	1.0946919	-0.2049646
P	1.0544447	-0.5887148	1.0576040
Cl	-3.1482048	-2.1297767	0.3202882
C	1.7491717	-2.2368034	1.4772479
C	2.9877795	-2.6857142	0.9692730

H	3.5667782	-2.0547672	0.2754923
C	3.4903455	-3.9358967	1.3649526
H	4.4657814	-4.2770271	0.9817462
C	2.7540501	-4.7504373	2.2438787
H	3.1495801	-5.7342181	2.5443372
C	1.5155677	-4.3100513	2.7440775
H	0.9374718	-4.9451935	3.4342689
C	1.0152537	-3.0533534	2.3718812
H	0.0551982	-2.7042227	2.7903546
C	1.2432775	0.3346606	2.6365305
C	0.0911534	0.7047917	3.3640200
H	-0.9110542	0.5048661	2.9477704
C	0.2142865	1.3059037	4.6283792
H	-0.6916816	1.5820714	5.1919892
C	1.4867502	1.5439727	5.1741755
H	1.5828650	2.0156097	6.1656071
C	2.6376901	1.1577895	4.4641877
H	3.6373089	1.3207923	4.8985831
C	2.5204347	0.5410449	3.2086999
H	3.4313000	0.1932312	2.6960466
C	-3.1515969	1.7967373	0.6145790
C	-3.3769455	3.1954696	0.5268107
H	-2.6504911	3.8530921	0.0252762
C	-4.5441204	3.7508654	1.0727696
H	-4.7160564	4.8364944	0.9913724
C	-5.4848135	2.9303521	1.7200324
H	-6.3952896	3.3734964	2.1554889
C	-5.2606624	1.5464201	1.8117477
H	-5.9941733	0.8970987	2.3162575
C	-4.1012215	0.9757616	1.2624075
H	-3.9425325	-0.1142031	1.3191254
C	-0.4370197	2.4551409	-0.1655865
C	-0.1642222	3.1327177	1.0471484
H	-0.6679016	2.8363295	1.9792158
C	0.7310918	4.2116103	1.0645110
H	0.9318058	4.7365558	2.0122272
C	1.3607084	4.6265606	-0.1231043
H	2.0598465	5.4786433	-0.1072175
C	1.0895164	3.9610732	-1.3308849
H	1.5698425	4.2908242	-2.2660622
C	0.1974769	2.8775619	-1.3567734
H	-0.0185237	2.3710724	-2.3090763
C	1.3180769	-3.3040522	-1.9627191
H	2.2506561	-2.7417031	-1.7954091
C	1.3895810	-4.6856816	-2.1843385
H	2.3654626	-5.1984047	-2.1714823
C	0.2127779	-5.4175765	-2.4299413
H	0.2651495	-6.5059719	-2.5995042
C	-1.0300577	-4.7578437	-2.4636520
H	-1.9533955	-5.3281321	-2.6556336
C	-1.0937856	-3.3746674	-2.2538921
H	-2.0752539	-2.8751256	-2.2755163
C	-1.1760277	-0.2511244	-2.5877741
C	-1.4454903	-0.5252886	-3.9510906
H	-0.8513335	-1.2932945	-4.4737340
C	-2.4652112	0.1422701	-4.6536499
H	-2.6475175	-0.1013819	-5.7133083
C	-3.2703013	1.0926585	-4.0044090
H	-4.0856211	1.5990896	-4.5455409
C	-3.0443959	1.3824601	-2.6490646
H	-3.6891651	2.1067789	-2.1257326
C	-1.9976516	0.7311497	-1.9661995
C	1.4036664	-0.2289481	-1.6306717
C	2.1147698	0.1990801	-2.7782685
H	1.7045977	-0.0174962	-3.7796301
C	3.3262041	0.9036164	-2.6794995
H	3.8530445	1.2180926	-3.5956335
C	3.8565971	1.2250324	-1.4181261
H	4.7944356	1.7976622	-1.3353443
C	3.1823235	0.8143424	-0.2580020
H	3.5916083	1.0827726	0.7277352
C	1.9823091	0.0797135	-0.3667932

9-*isomer*

Energy = -2914.628313162

Cl	-2.1101952	-1.3913410	2.9180724
Pd	-1.3845408	-0.1425422	1.1021370
B	0.0904060	-1.0464878	-1.9031183
C	0.0653932	-2.6025408	-1.8550546
P	-1.4548161	1.4360472	-0.5485616
P	0.8360611	-0.5872221	1.3124674
C	1.3842865	-2.3389113	1.4556255
C	2.7001418	-2.6655789	1.0466940
H	3.3336575	-1.9192375	0.5407530
C	3.2087892	-3.9516654	1.2883877
H	4.2385407	-4.1958008	0.9797700
C	2.4073227	-4.9225410	1.9132807
H	2.8073228	-5.9332542	2.0965682
C	1.0942859	-4.6035961	2.2993091
H	0.4586696	-5.3625787	2.7834345
C	0.5798855	-3.3156330	2.0785900
H	-0.4447459	-3.0624137	2.3978244
C	1.4255556	0.2069493	2.8708064
C	0.6038543	1.1171833	3.5705560
H	-0.4152304	1.3281670	3.2063872
C	1.0549376	1.7099663	4.7611350
H	0.3973221	2.4065088	5.3059922
C	2.3283508	1.3984695	5.2659425
H	2.6792793	1.8582816	6.2040822
C	3.1449888	0.4797669	4.5840530
H	4.1366945	0.2145335	4.9849251
C	2.6963053	-0.1227281	3.3978405
H	3.3358134	-0.8656522	2.8955801
C	-3.1070212	1.7582648	0.1824705
C	-3.4442417	2.9386152	0.8883224
H	-2.7567570	3.7998090	0.8891892
C	-4.6649396	3.0136684	1.5719865
H	-4.9347096	3.9402837	2.1041288
C	-5.5484468	1.9144341	1.5831665
H	-6.5014130	1.9824481	2.1323496
C	-5.2214739	0.7387636	0.8912048
H	-5.9119569	-0.1193576	0.8911688

C	-4.0086312	0.6546414	0.1843943
H	-3.7841983	-0.2519471	-0.4046782
C	-0.4709394	2.9724021	-0.5739064
C	-0.0038489	3.5153249	0.6452960
H	-0.2022943	2.9971184	1.5969722
C	0.7268094	4.7131234	0.6463356
H	1.0844617	5.1330492	1.6003441
C	1.0092871	5.3680036	-0.5657981
H	1.5861540	6.3071905	-0.5630400
C	0.5640747	4.8202773	-1.7810957
H	0.7909733	5.3268154	-2.7330710
C	-0.1724200	3.6247911	-1.7912544
H	-0.5149905	3.2014411	-2.7485627
C	1.2655585	-3.3647370	-1.8457324
H	2.2360757	-2.8421858	-1.8252379
C	1.2412599	-4.7650954	-1.9015892
H	2.1847100	-5.3344526	-1.9199172
C	0.0093217	-5.4442833	-1.9266207
H	-0.0116964	-6.5463402	-1.9573559
C	-1.1967558	-4.7187948	-1.9165562
H	-2.1615736	-5.2517900	-1.9327830
C	-1.1655168	-3.3181240	-1.9023824
H	-2.1167954	-2.7592718	-1.9125481
C	-1.0503592	-0.2986052	-2.7166492
C	-1.3661333	-0.7596882	-4.0202930
H	-0.8666985	-1.6639916	-4.4051647
C	-2.2938525	-0.0904691	-4.8374472
H	-2.5061235	-0.4698544	-5.8504487
C	-2.9675154	1.0444556	-4.3573253
H	-3.7112014	1.5610842	-4.9855500
C	-2.7038880	1.5109733	-3.0585068
H	-3.2582410	2.3801756	-2.6669990
C	-1.7407277	0.8576718	-2.2599228
C	1.3488883	-0.1771064	-1.4425834
C	2.1140203	0.3776366	-2.4989070
H	1.8000687	0.1981988	-3.5416863
C	3.2635890	1.1531192	-2.2650945
H	3.8359238	1.5559657	-3.1167110
C	3.6580144	1.4354148	-0.9500112
H	4.5390400	2.0667361	-0.7513647
C	2.9114442	0.9203266	0.1240011
H	3.2116027	1.1634701	1.1552391
C	1.7857477	0.1042523	-0.1132551

10-*endo*

Energy = -2571.721946633

Thermal correction to Gibbs free Energy = 1551.06 KJ/mol

Pd	0.0400802	-0.1228735	-1.3920401
P	-1.6369685	-0.8372907	0.0671105
P	1.0484415	1.6363937	-0.1281826
B	1.6361219	-1.4431575	0.3524647
C	1.9273795	-2.6611396	-0.6075540
C	1.2249373	0.3472268	-3.2476960
H	2.3220333	0.4486238	-3.2038505
C	-0.7595015	-1.0988048	-3.2289035
C	-2.6782805	0.5995714	0.5685592
C	-2.8972108	0.9881335	1.9054655
H	-2.4169430	0.4415822	2.7307569
C	-3.7267657	2.0867615	2.1939805
H	-3.8906473	2.3803347	3.2432947
C	-4.3447572	2.8032799	1.1566251
H	-4.9999348	3.6587633	1.3883906
C	-4.1228744	2.4274361	-0.1807564
H	-4.6040720	2.9849390	-1.0009960
C	-3.2896712	1.3369455	-0.4741898
H	-3.1275805	1.0453959	-1.5264660
C	-2.8761992	-2.1007157	-0.4796892
C	-2.4064613	-3.3185060	-1.0237985
H	-1.3234146	-3.4917770	-1.1359816
C	-3.3103811	-4.3178160	-1.4150243
H	-2.9306245	-5.2635629	-1.8347961
C	-4.6949642	-4.1129601	-1.2737298
H	-5.4048405	-4.8967199	-1.5842263
C	-5.1682785	-2.9062066	-0.7347984
H	-6.2515534	-2.7391641	-0.6176140
C	-4.2667002	-1.9024018	-0.3371337
H	-4.6565932	-0.9644461	0.0873395
C	-0.8236713	-1.5701644	1.5396981
C	0.5950401	-1.6688368	1.5613295
C	1.1764206	-2.1756027	2.7547898
H	2.2742448	-2.2566591	2.8282691
C	0.3988411	-2.6005051	3.8435027
H	0.8922579	-2.9946770	4.7473208
C	-1.0041311	-2.5422604	3.7742424
H	-1.6222817	-2.8963128	4.6151369
C	-1.6124443	-2.0278544	2.6211336
H	-2.7127543	-1.9907872	2.5572750
C	2.9739971	-2.6021930	-1.5710883
H	3.5475399	-1.6659506	-1.6819007
C	3.3011228	-3.7031682	-2.3763992
H	4.1192119	-3.6277631	-3.1117774
C	2.5920109	-4.9112883	-2.2337781
H	2.8514824	-5.7818761	-2.8587493
C	1.5651590	-5.0096773	-1.2767623
H	1.0262633	-5.9624139	-1.1438695
C	1.2419344	-3.9009708	-0.4790309
H	0.4601143	-4.0062859	0.2916013
C	2.8059247	-0.3766007	0.5952065
C	2.6430726	1.0310865	0.5392825
C	3.6370740	1.9114956	1.0202030
H	3.4654897	3.0004671	1.0238593
C	4.8484971	1.3997278	1.5099113
H	5.6275073	2.0863079	1.8791107
C	5.0600679	0.0102479	1.5200639
H	6.0153754	-0.3997991	1.8872088
C	4.0522393	-0.8609435	1.0739809
H	4.2338636	-1.9484163	1.1070087
C	1.3412798	2.9893491	-1.3517752
C	2.6266784	3.4090120	-1.7537260
H	3.5267524	2.9571799	-1.3101032
C	2.7712910	4.4006386	-2.7411702

H	3.7821864	4.7188833	-3.0440909
C	1.6412407	4.9821295	-3.3379948
H	1.7603420	5.7616275	-4.1077875
C	0.3563740	4.5555537	-2.9569243
H	-0.5367578	4.9964910	-3.4291205
C	0.2059232	3.5593094	-1.9790678
H	-0.8077888	3.2137289	-1.7115367
C	0.3011556	2.4840191	1.3451704
C	0.3943923	1.8284412	2.5958372
H	0.8524627	0.8293395	2.6680407
C	-0.0695861	2.4550035	3.7626266
H	0.0268646	1.9378313	4.7313106
C	-0.6394847	3.7385850	3.6984907
H	-0.9929561	4.2348666	4.6171926
C	-0.7424764	4.3911301	2.4592594
H	-1.1761646	5.4026655	2.3997438
C	-0.2744059	3.7716104	1.2876845
H	-0.3359680	4.3187716	0.3353688
H	0.6702804	1.2533732	-3.5528055
C	0.6388856	-0.9335290	-3.3859901
H	1.2860778	-1.8278127	-3.3467244
H	-1.1983441	-2.1088660	-3.1933307
H	-1.4514992	-0.2855037	-3.5219174

10-*exo*

Energy = -2571.721941589

Thermal correction to Gibbs free Energy = 1547.98 KJ/mol

Pd	0.0605270	-0.1253245	-1.3864941
P	-1.6012272	-0.8851530	0.0659029
P	1.0614557	1.6263127	-0.1050744
B	1.6586674	-1.4509596	0.3882072
C	1.9116326	-2.6354138	-0.6202441
C	1.3778719	0.2393500	-3.2071067
H	1.7465129	1.2556549	-3.4180053
H	2.1385757	-0.5364547	-3.0156214
C	0.0744162	-0.1317684	-3.6041639
H	-0.5909381	0.6401345	-4.0357942
C	-0.4887295	-1.3605696	-3.1681234
H	0.1624190	-2.2382802	-3.0066613
C	-2.6405823	0.5545162	0.5647944
C	-2.8592163	0.9456924	1.9007599
H	-2.3761280	0.4026664	2.7268688
C	-3.6951025	2.0403372	2.1868737
H	-3.8603658	2.3358072	3.2354310
C	-4.3181146	2.7499156	1.1477683
H	-4.9783244	3.6020619	1.3775818
C	-4.0957661	2.3710810	-0.1888857
H	-4.5815720	2.9231832	-1.0100590
C	-3.2576217	1.2840184	-0.4801012
H	-3.0950863	0.9878254	-1.5311000
C	-2.8563938	-2.1329338	-0.4832692
C	-2.4213028	-3.3035576	-1.1453368
H	-1.3508036	-3.4509230	-1.3593819
C	-3.3444133	-4.2877330	-1.5311270
H	-2.9907483	-5.1955903	-2.0464156
C	-4.7146792	-4.1144779	-1.2658216
H	-5.4397402	-4.8856549	-1.5727555
C	-5.1549986	-2.9534609	-0.6101518
H	-6.2271258	-2.8099658	-0.3979186
C	-4.2341413	-1.9651494	-0.2194041
H	-4.5985586	-1.0608523	0.2921649
C	-0.8026216	-1.6155132	1.5487308
C	0.6172454	-1.6872689	1.5908129
C	1.1936107	-2.1791866	2.7924941
H	2.2918188	-2.2370076	2.8811394
C	0.4091247	-2.6202581	3.8700203
H	0.8973647	-3.0041597	4.7810052
C	-0.9934876	-2.5891898	3.7811838
H	-1.6159896	-2.9538443	4.6143010
C	-1.5966782	-2.0860900	2.6199345
H	-2.6966263	-2.0662232	2.5431845
C	2.9602283	-2.5736087	-1.5812944
H	3.5886040	-1.6681057	-1.6308855
C	3.2281786	-3.6388694	-2.4529774
H	4.0466262	-3.5613186	-3.1875020
C	2.4572566	-4.8148032	-2.3793103
H	2.6678943	-5.6569471	-3.0592311
C	1.4339793	-4.9214557	-1.4184804
H	0.8511807	-5.8539220	-1.3369407
C	1.1695895	-3.8477728	-0.5548131
H	0.3845017	-3.9570847	0.2115846
C	2.8239515	-0.3839309	0.6326471
C	2.6544294	1.0235443	0.5759671
C	3.6430656	1.9052768	1.0656609
H	3.4667918	2.9933896	1.0747184
C	4.8541231	1.3970189	1.5603100
H	5.6276356	2.0859154	1.9366681
C	5.0727775	0.0087105	1.5663752
H	6.0283521	-0.3981216	1.9363245
C	4.0701700	-0.8653810	1.1141711
H	4.2551924	-1.9524466	1.1467075
C	1.3621945	2.9856363	-1.3218680
C	2.6471796	3.4367486	-1.6903642
H	3.5465985	3.0059084	-1.2254445
C	2.7934609	4.4301753	-2.6759795
H	3.8039519	4.7720872	-2.9534430
C	1.6654130	4.9835426	-3.3029292
H	1.7856178	5.7644792	-4.0711079
C	0.3818767	4.5266719	-2.9538332
H	-0.5091626	4.9454857	-3.4495731
C	0.2303571	3.5275814	-1.9790312
H	-0.7807790	3.1570479	-1.7369968
C	0.3030981	2.4740653	1.3623154
C	0.4032755	1.8304583	2.6185596
H	0.8745724	0.8381714	2.6996967
C	-0.0712447	2.4606441	3.7791874
H	0.0308540	1.9535080	4.7525956
C	-0.6589161	3.7354859	3.7028380
H	-1.0204526	4.2349533	4.6166673
C	-0.7703400	4.3751724	2.4575716
H	-1.2191816	5.3794716	2.3885515

C -0.2920471 3.7520062 1.2921911
H -0.3620660 4.2882507 0.3341542
H -1.5529477 -1.5732318 -3.3635997

13
Energy = -2724.043534737
Pd 0.1633142 0.2488733 0.7596807
P -0.1641606 -0.9906004 2.8341447
P 1.9647934 -0.7327426 -0.3157538
P -1.8911101 1.1245386 0.0629471
C 4.3783516 0.3361470 -1.4785070
H 4.8444177 -0.6583154 -1.3734050
C 2.4160245 1.8143901 -1.1306493
C 3.1615498 -1.8010966 0.6317922
C 3.9918081 -1.1771410 1.5936975
H 3.9547758 -0.0809134 1.7167551
C 3.0455806 0.5480001 -1.0606730
C -1.5967804 2.3324737 -1.2951278
C -3.6249676 -1.1236619 0.0639816
H -3.7020465 -0.9529144 1.1508725
C -2.8633974 -0.2384510 -0.7355330
C 1.4471462 -1.8592938 -1.6983907
C -4.5426164 1.9554052 0.8718932
H -4.9284623 1.4262874 -0.0148324
C -2.7005039 2.6019725 2.3190664
H -1.6242992 2.5815617 2.5609363
C -0.2212911 2.5427666 -1.5872919
C 0.0545988 3.3073054 -2.7506953
H 1.1010846 3.4643784 -3.0595766
C -3.5959547 3.2870460 3.1577971
H -3.2183904 3.8067594 4.0542971
C -3.1595612 1.9348164 1.1618460
C -2.7874061 -0.4786586 -2.1252963
H -2.1926230 0.1923458 -2.7647901
C -1.7155448 -0.8068842 3.8484307
H -1.8927692 0.2668283 4.0712343
H -1.6559271 -1.3759604 4.8041896
H -2.5854968 -1.1754256 3.2636545
C 3.2051154 2.8761923 -1.6481381
H 2.7882186 3.8976230 -1.6817282
C 0.6049161 -3.6334790 -3.7343011
H 0.2768635 -4.3238207 -4.5294691
C -2.6296355 2.9267482 -2.0479785
H -3.6856565 2.7578570 -1.7772227
C -5.4392981 2.6350092 1.7144487
H -6.5158067 2.6412319 1.4739237
C 5.1191658 1.4050699 -2.0043113
H 6.1604740 1.2511520 -2.3337420
C -0.1404171 -3.5163282 -2.5482416
H -1.0605695 -4.1071430 -2.4091762
C -4.9689381 3.3047328 2.8574120
H -5.6741570 3.8378800 3.5166228
C 4.5256710 2.6787448 -2.0845710
H 5.1074453 3.5318629 -2.4743324
C 3.2308910 -3.2036628 0.4876089
H 2.6011379 -3.7106421 -0.2614428
C 4.8725490 -1.9349121 2.3813041
H 5.5204588 -1.4292153 3.1170112
C -3.4664413 -1.5670908 -2.6998773
H -3.3978263 -1.7345387 -3.7876444
C -0.9658436 3.8792279 -3.5285860
H -0.7053319 4.4691464 -4.4243789
C 0.2721541 -2.6271696 -1.5415191
H -0.3331753 -2.5083496 -0.6270086
C -2.3154214 3.7104027 -3.1690712
H -3.1186893 4.1670177 -3.7713533
C -4.2299745 -2.4336455 -1.8993336
H -4.7682569 -3.2821003 -2.3538233
C -0.0320629 -2.8444423 2.7591105
H -0.8479647 -3.2434051 2.1182424
H -0.1051023 -3.3073245 3.7695112
H 0.9394973 -3.1252924 2.2981350
C 4.9308721 -3.3335156 2.2328078
H 5.6212154 -3.9292757 2.8528135
C 2.1848021 -1.9764062 -2.8965670
H 3.0902661 -1.3672887 -3.0481659
C 1.1272344 -0.5884138 4.1116196
H 2.1312216 -0.8253468 3.6984540
H 0.9734074 -1.1630839 5.0532941
H 1.0945434 0.4990994 4.3388836
C -4.3051873 -2.2082924 -0.5127511
H -4.9032958 -2.8798357 0.1261665
C 1.7628499 -2.8557198 -3.9084188
H 2.3447953 -2.9307223 -4.8423793
C 4.1074087 -3.9630927 1.2853172
H 4.1503265 -5.0577189 1.1557357
B 0.9231780 2.0649167 -0.5414455
C 0.9845564 2.9928463 0.8075415
H -0.0027760 3.3809950 1.1402738
H 1.6180794 3.8873594 0.5769223
H 1.4823775 2.4963224 1.6771242

14-B
Energy = -3024.980302321
Thermal correction to Gibbs free Energy = 1780.59 KJ/mol
Pd -0.3099338 0.3590997 0.8761287
C -0.4537005 2.3279141 1.3986003
N -0.1452526 -0.1300127 2.9136439
B 2.1624474 -0.4033731 -0.3072076
P -0.2435690 -2.1525649 0.8278063
P -0.6196841 0.8506353 -1.4013038
C -0.2196228 2.4825929 -2.1985713
C 0.5760022 2.5694680 -3.3618303
C 0.8148326 3.8132288 -3.9735133
C 0.2627385 4.9851499 -3.4321397
C -0.5382652 4.9062186 -2.2795067
C -0.7795669 3.6666178 -1.6665535
C -2.4059242 0.6835987 -1.8869641
C -3.3186508 0.1448235 -0.9527586
C -4.6845393 0.0353509 -1.2665696
C -5.1558185 0.4667443 -2.5179792

C	-4.2586009	1.0196314	-3.4489581
C	-2.8947121	1.1369664	-3.1335692
C	-1.2643247	-2.9671902	2.1496628
C	-2.5194568	-3.5310184	1.8269144
C	-3.3419389	-4.0776106	2.8269806
C	-2.9199912	-4.0790990	4.1667137
C	-1.6695604	-3.5301557	4.4972897
C	-0.8488388	-2.9721116	3.5019806
C	-0.5790301	-3.3184520	-0.6015912
C	-1.6876870	-3.0719759	-1.4410223
C	-2.0269861	-3.9678718	-2.4699741
C	-1.2510381	-5.1177987	-2.6881763
C	-0.1306246	-5.3606828	-1.8749630
C	0.2032297	-4.4696659	-0.8413180
C	3.0194259	0.9222028	-0.2237603
C	3.2936732	1.6919731	-1.3857959
C	4.1272566	2.8202566	-1.3525474
C	4.7206663	3.2196543	-0.1425280
C	4.4753872	2.4788498	1.0283926
C	3.6419907	1.3514103	0.9823824
C	2.4878369	-1.5906094	0.7159962
C	3.8502680	-1.8586601	1.0109747
C	4.2456947	-2.9728926	1.7720686
C	3.2770676	-3.8562523	2.2702765
C	1.9174483	-3.6180112	2.0003728
C	1.5229212	-2.5020053	1.2356873
C	1.5595666	-0.8451189	-1.7344305
C	2.3360557	-1.7834966	-2.4613173
C	1.9658862	-2.2274596	-3.7410812
C	0.7824506	-1.7565801	-4.3323043
C	-0.0116510	-0.8298873	-3.6390790
C	0.3756679	-0.3681338	-2.3640767
C	0.6482691	3.2066833	1.4950664
C	-1.7407962	2.8402350	1.7071479
C	0.4853178	4.5433847	1.8920130
C	-1.9246911	4.1748937	2.1002600
C	-0.8029397	5.0156120	2.1882724
C	1.0227796	0.3538283	3.6180284
C	-1.3561436	0.1759007	3.6525699
N	-0.9852782	6.4246345	2.5950611
O	-2.1351501	6.8083084	2.8385675
O	0.0211450	7.1384856	2.6650269
H	1.0064102	1.6582882	-3.8064154
H	1.4370242	3.8610753	-4.8828595
H	0.4533655	5.9608035	-3.9091067
H	-0.9801478	5.8190160	-1.8472799
H	-1.4130679	3.6245526	-0.7671650
H	-2.9498053	-0.1781764	0.0366162
H	-5.3844362	-0.3804884	-0.5231038
H	-6.2277002	0.3861457	-2.7635830
H	-4.6248584	1.3757334	-4.4261321
H	-2.2097690	1.6085848	-3.8571697
H	-2.8627939	-3.5584858	0.7809297
H	-4.3172497	-4.5116141	2.5503336
H	-3.5621780	-4.5123862	4.9514967
H	-1.3247183	-3.5305874	5.5448981
H	0.1158043	-2.5246932	3.7759344
H	-2.3002135	-2.1681475	-1.2955948
H	-2.9009623	-3.7573258	-3.1084032
H	-1.5135945	-5.8209245	-3.4961186
H	0.4923122	-6.2551507	-2.0429810
H	1.0853760	-4.6761173	-0.2156415
H	2.8574990	1.3817668	-2.3476329
H	4.3174537	3.3914727	-2.2763678
H	5.3762239	4.1063316	-0.1114069
H	4.9368723	2.7840467	1.9824078
H	3.4603765	0.7870480	1.9106033
H	4.6265413	-1.1788248	0.6227427
H	5.3153420	-3.1458404	1.9783988
H	3.5731476	-4.7316256	2.8721451
H	1.1593152	-4.3130453	2.3952389
H	3.2557875	-2.1868992	-2.0042543
H	2.5956577	-2.9620543	-4.2703287
H	0.4689323	-2.1185614	-5.3252698
H	-0.9516644	-0.4756178	-4.0920661
H	1.6614985	2.8500810	1.2542493
H	-2.6310521	2.1924904	1.6389041
H	1.3402233	5.2307922	1.9703164
H	-2.9191195	4.5792473	2.3394284
H	1.9508334	0.0304995	3.0999686
H	1.0481347	-0.0920403	4.6481710
H	1.0668165	1.4694274	3.7550586
H	-2.2525257	-0.2255166	3.1271316
H	-1.5248348	1.2707739	3.8434608
H	-1.3223146	-0.3197171	4.6579518

15-B

Energy = -3024.968983248

Thermal correction to Gibbs free Energy = 1777.25 KJ/mol

Imaginary frequency = 271.84 cm^{-1}

Pd	-0.3313217	0.2897352	0.8211785
C	-0.5713709	2.1589563	1.6493600
N	-0.3927788	0.5144992	2.9165144
B	2.2258684	-0.4969446	-0.4148481
P	-0.2016513	-2.2321888	0.7510055
P	-0.5524893	0.7838636	-1.4370810
C	-0.0854794	2.4273971	-2.1685327
C	0.6422790	2.5332717	-3.3746427
H	0.9896675	1.6254922	-3.8937444
C	0.9264602	3.7946155	-3.9272435
H	1.4932510	3.8596213	-4.8711695
C	0.4909992	4.9637123	-3.2811564
H	0.7173910	5.9531059	-3.7120749
C	-0.2362819	4.8653501	-2.0825743
H	-0.5817332	5.7754970	-1.5656999
C	-0.5252759	3.6076023	-1.5283970
H	-1.1023134	3.5496620	-0.5926994
C	-2.3343910	0.6907986	-1.9597754
C	-3.2899228	0.1912612	-1.0476808
H	-2.9541507	-0.1433801	-0.0504232
C	-4.6525024	0.1428416	-1.3891841

H	-5.3861490	-0.2419496	-0.6615389
C	-5.0781174	0.5977638	-2.6485927
H	-6.1472398	0.5659540	-2.9164718
C	-4.1382264	1.1151268	-3.5573804
H	-4.4683111	1.4927276	-4.5393651
C	-2.7773515	1.1715733	-3.2130831
H	-2.0587285	1.6190731	-3.9191289
C	-1.0749904	-2.9989633	2.1978192
C	-2.2568059	-3.7586109	2.0533435
H	-2.6503681	-3.9845132	1.0495983
C	-2.9342293	-4.2497619	3.1829498
H	-3.8522883	-4.8458724	3.0487036
C	-2.4408540	-3.9939470	4.4732622
H	-2.9708493	-4.3830470	5.3584249
C	-1.2621324	-3.2433704	4.6266748
H	-0.8617484	-3.0418722	5.6343973
C	-0.5874502	-2.7435781	3.5013704
H	0.3334977	-2.1558756	3.6372010
C	-0.6884292	-3.3586457	-0.6466694
C	-1.9233366	-3.1370971	-1.2966262
H	-2.5588358	-2.2870318	-1.0013461
C	-2.3541704	-3.9858884	-2.3297317
H	-3.3230356	-3.7963414	-2.8208997
C	-1.5479212	-5.0596069	-2.7423546
H	-1.8814470	-5.7239294	-3.5569489
C	-0.3080435	-5.2749069	-2.1172946
H	0.3366323	-6.1097140	-2.4398186
C	0.1198701	-4.4329517	-1.0772957
H	1.0941459	-4.6165007	-0.5979383
C	3.0254155	0.8399103	-0.1871690
C	3.2713369	1.7512055	-1.2486069
H	2.8556414	1.5386699	-2.2454974
C	4.0471720	2.9062819	-1.0692476
H	4.2159750	3.5927041	-1.9155015
C	4.6074545	3.1881164	0.1889482
H	5.2152672	4.0970573	0.3344141
C	4.3919705	2.3027585	1.2615407
H	4.8317774	2.5155209	2.2502450
C	3.6171692	1.1496292	1.0703985
H	3.4667480	0.4595644	1.9167855
C	2.5463382	-1.7401567	0.5383021
C	3.9105430	-2.0503487	0.7819760
H	4.6918092	-1.3717740	0.4005939
C	4.2983425	-3.2001342	1.4910969
H	5.3687487	-3.4073681	1.6584307
C	3.3218435	-4.0733245	1.9954089
H	3.6138659	-4.9736072	2.5613563
C	1.9625540	-3.7896621	1.7798245
H	1.1953803	-4.4671530	2.1881236
C	1.5751304	-2.6438499	1.0537992
C	1.6326481	-0.8787241	-1.8593355
C	2.4178587	-1.7739379	-2.6302434
H	3.3633917	-2.1579276	-2.2104384
C	2.0219549	-2.2039406	-3.9072944
H	2.6599878	-2.9034994	-4.4730332
C	0.8008633	-1.7681215	-4.4460764
H	0.4639855	-2.1257202	-5.4329818
C	0.0008528	-0.8792305	-3.7103974
H	-0.9649353	-0.5488244	-4.1248503
C	0.4175490	-0.4235122	-2.4429085
C	0.5246478	3.0609773	1.7820887
C	-1.8902883	2.7043617	1.7321708
C	0.3191716	4.4244947	1.9996758
H	1.5569161	2.6906871	1.6871791
C	-2.1007259	4.0690944	1.9466460
H	-2.7700972	2.0534488	1.6060439
C	-0.9943727	4.9317752	2.0828131
H	1.1642742	5.1223283	2.0898467
H	-3.1142393	4.4929526	2.0009362
C	0.7317877	0.8413594	3.7622097
H	0.6457214	1.8536531	4.2429505
H	0.8258868	0.0882273	4.5867695
H	1.6788107	0.8211909	3.1871959
C	-1.6476425	0.4417193	3.6331803
H	-1.5440407	-0.2150162	4.5310998
H	-2.0051763	1.4463317	3.9896445
H	-2.4469573	0.0012127	2.9997579
N	-1.2109752	6.3589431	2.2946566
O	-2.3820040	6.7665822	2.3666635
O	-0.2126770	7.0920624	2.3884181

***N,N*-dimethyl-4-nitroaniline**

Energy = -570.3403485962

Thermal correction to Gibbs free Energy = 346.03 KJ/mol

C	-1.7802913	-0.8855041	0.7203246
C	-0.3941680	-1.0173092	0.6454815
C	0.4066993	-0.0365035	-0.0203020
C	-0.2683323	1.0816035	-0.6041778
C	-1.6548540	1.2076474	-0.5260599
C	-2.4117364	0.2253479	0.1351110
H	-2.3973797	-1.6385091	1.2313949
H	0.0727066	-1.8959292	1.1114866
H	0.2985516	1.8636339	-1.1276864
H	-2.1746293	2.0664792	-0.9747756
N	-3.8706874	0.3595663	0.2141969
O	-4.3875180	1.3510793	-0.3168196
O	-4.4987632	-0.5272792	0.8069766
C	2.4391843	-1.3155258	0.4997817
H	2.0894283	-2.2755842	0.0516318
H	3.5310800	-1.2395882	0.3297721
H	2.2679190	-1.3701346	1.6009505
C	2.5691771	0.8478872	-0.7808982
H	2.4551140	1.8535054	-0.3109283
H	3.6403780	0.5709315	-0.7310480
H	2.2897192	0.9386218	-1.8573702
N	1.7784019	-0.1644365	-0.0970421

14-O

Energy = -2843.821886560

Thermal correction to Gibbs free Energy = 1560.69 KJ/mol

O	1.0761768	-0.5424897	-2.3272881
P	-1.1824201	1.2443084	-1.2798935
P	1.5476834	-0.5106095	0.5661211
C	2.4329997	-2.1417658	0.5088347
C	3.0919299	-2.5884633	-0.6571198
H	3.1225887	-1.9546779	-1.5558281
C	3.7235012	-3.8437798	-0.6830112
H	4.2354459	-4.1749681	-1.6019243
C	3.7105667	-4.6679126	0.4545923
H	4.2094969	-5.6509691	0.4342093
C	3.0574324	-4.2306085	1.6196229
H	3.0367947	-4.8689061	2.5180318
C	2.4173906	-2.9810536	1.6469786
H	1.9072632	-2.6564920	2.5675787
C	2.1270295	0.2059194	2.1750423
C	1.2344001	1.0176389	2.9073504
H	0.2076987	1.1608435	2.5262149
C	1.6330942	1.6109522	4.1174585
H	0.9226837	2.2391614	4.6798616
C	2.9284565	1.3899997	4.6147342
H	3.2412871	1.8473745	5.5680570
C	3.8213360	0.5723835	3.8992690
H	4.8358217	0.3872236	4.2898173
C	3.4250091	-0.0176468	2.6873216
H	4.1291610	-0.6657381	2.1408793
C	-2.9221592	1.8595141	-1.2898914
C	-3.2465658	3.1737780	-0.8933182
H	-2.4477277	3.8990514	-0.6720790
C	-4.5903114	3.5741863	-0.7750679
H	-4.8259896	4.6070377	-0.4691722
C	-5.6231214	2.6620333	-1.0475834
H	-6.6767520	2.9748449	-0.9552964
C	-5.3097544	1.3515074	-1.4481163
H	-6.1162140	0.6342710	-1.6745648
C	-3.9689842	0.9414611	-1.5559649
H	-3.7396187	-0.0778201	-1.8929445
C	-0.2258723	2.8377691	-1.3303462
C	0.3066056	3.3389051	-0.1246002
H	0.2199578	2.7432350	0.7984563
C	0.9427630	4.5923762	-0.0897415
H	1.3514753	4.9711414	0.8616711
C	1.0607692	5.3542146	-1.2637614
H	1.5609851	6.3366839	-1.2385244
C	0.5417488	4.8573813	-2.4728961
H	0.6317733	5.4499550	-3.3986659
C	-0.0984975	3.6080884	-2.5068016
H	-0.5086197	3.2317824	-3.4583690
C	0.1278769	-0.2713597	-3.3042255
C	0.2620022	-0.8999486	-4.5524529
H	1.1213554	-1.5682360	-4.7184789
C	-0.6928796	-0.6693108	-5.5553011
H	-0.5892784	-1.1626698	-6.5356091
C	-1.7715177	0.1937666	-5.3026051
H	-2.5212386	0.3910456	-6.0860282
C	-1.9088849	0.7994834	-4.0420517
H	-2.7738963	1.4527696	-3.8477831
C	-0.9724360	0.5708872	-3.0085830
C	2.0433573	0.4088414	-2.0425182
C	2.6171865	1.2086060	-3.0454086
H	2.2626593	1.1195873	-4.0837095
C	3.6378646	2.1097328	-2.7074066
H	4.0891868	2.7376364	-3.4930732
C	4.0800940	2.2097366	-1.3777657
H	4.8792749	2.9190573	-1.1089545
C	3.4939947	1.4095789	-0.3839360
H	3.8310914	1.5106567	0.6591714
C	2.4656660	0.4955086	-0.6955785
C	-0.8284801	-2.0812067	1.7615699
C	-0.7894722	-3.4177554	1.2902768
C	-0.9321889	-1.8800196	3.1598930
C	-0.8609203	-4.5095181	2.1673280
H	-0.7022991	-3.6186754	0.2095722
C	-0.9978007	-2.9587382	4.0561751
H	-0.9649364	-0.8588086	3.5738511
C	-0.9602202	-4.2670395	3.5475573
H	-0.8345518	-5.5479632	1.8061973
H	-1.0786965	-2.8078405	5.1426888
Pd	-0.8076785	-0.5371609	0.4345282
N	-1.0224334	-5.4092194	4.4821249
O	-1.1151359	-5.1605273	5.6898041
O	-0.9740987	-6.5482099	4.0037277
C	-3.4457222	0.0855116	1.6033423
H	-4.5543898	0.2045405	1.4727851
H	-3.2967946	-0.4019187	2.6079890
H	-3.0087663	1.1097068	1.6513008
C	-3.5500830	-1.9352077	0.3357951
H	-3.5411421	-2.5917036	1.2478204
H	-4.6315423	-1.7678290	0.0801328
H	-3.0975982	-2.5136231	-0.4999538
N	-2.8952449	-0.6532855	0.4884594

15-O

Energy = -2843.811055445

Thermal correction to Gibbs free Energy = 1,563.60 KJ/mol

Imaginary frequency = -275 cm^{-1}

Pd	-0.8624268	-0.4521712	0.0357801
C	-1.6228599	-2.3568049	0.0542703
N	-2.9411039	-0.7533811	0.1214345
O	1.2275003	1.0497691	-2.1076962
P	-0.7757186	2.0305392	-0.0373646
P	1.4007034	-1.0201591	-0.0214617
C	1.8924493	-2.2842529	-1.2815258
C	2.6546559	-1.9581601	-2.4240193
H	3.0185698	-0.9302862	-2.5735114
C	2.9667335	-2.9448276	-3.3747769
H	3.5659536	-2.6755834	-4.2605751
C	2.5249224	-4.2668478	-3.1954825
H	2.7712450	-5.0400944	-3.9419944
C	1.7678045	-4.5992217	-2.0590092
H	1.4108275	-5.6313803	-1.9093405
C	1.4476361	-3.6156360	-1.1091198

H	0.8546031	-3.8936311	-0.2233613
C	1.9885415	-1.7804496	1.5633309
C	1.2677661	-1.5161386	2.7475003
H	0.3518240	-0.9026780	2.6898457
C	1.6902305	-2.0488713	3.9773114
H	1.1137582	-1.8381364	4.8932185
C	2.8331722	-2.8639296	4.0338700
H	3.1601631	-3.2925623	4.9957098
C	3.5505253	-3.1445113	2.8574047
H	4.4416672	-3.7930254	2.8944266
C	3.1322232	-2.6076935	1.6283916
H	3.6957572	-2.8425954	0.7106053
C	-2.4115415	2.7581503	0.4365362
C	-2.5838461	3.5407229	1.5985622
H	-1.7133311	3.8160680	2.2144429
C	-3.8649857	3.9765146	1.9815817
H	-3.9808182	4.5888280	2.8914391
C	-4.9887803	3.6395630	1.2095662
H	-5.9922249	3.9840313	1.5097857
C	-4.8257981	2.8610364	0.0495922
H	-5.7012886	2.5928183	-0.5651229
C	-3.5503063	2.4140126	-0.3289661
H	-3.4385389	1.8044843	-1.2388240
C	0.3949239	3.0325844	0.9988244
C	0.8819238	2.4665469	2.1957809
H	0.6170157	1.4281901	2.4533290
C	1.7014117	3.2156113	3.0580905
H	2.0736081	2.7605936	3.9907558
C	2.0508923	4.5353301	2.7269357
H	2.6962015	5.1228162	3.4010982
C	1.5792548	5.1033507	1.5297373
H	1.8529130	6.1377273	1.2625727
C	0.7552540	4.3581706	0.6710957
H	0.3862279	4.8142137	-0.2622381
C	0.4571244	2.0974221	-2.5906890
C	0.6141766	2.4768823	-3.9333746
H	1.3626308	1.9513606	-4.5467080
C	-0.1822876	3.5034835	-4.4652017
H	-0.0606096	3.8015338	-5.5194337
C	-1.1294296	4.1440278	-3.6491824
H	-1.7546221	4.9554648	-4.0560846
C	-1.2943669	3.7429513	-2.3125692
H	-2.0604096	4.2344002	-1.6919390
C	-0.5133168	2.7056073	-1.7547267
C	2.3151729	1.3190057	-1.2933525
C	3.1226066	2.4545275	-1.4813910
H	2.8657331	3.1831905	-2.2654790
C	4.2510479	2.6408368	-0.6679817
H	4.8850530	3.5303715	-0.8154786
C	4.5700776	1.6987461	0.3241029
H	5.4547756	1.8422412	0.9648018
C	3.7512997	0.5722386	0.5074240
H	3.9944647	-0.1518905	1.3000255
C	2.6089692	0.3613282	-0.2932348
C	-1.7643246	-3.0708857	-1.1764426
C	-1.6593692	-3.1220473	1.2612457
C	-1.9366082	-4.4555399	-1.2017410
H	-1.7143518	-2.5297937	-2.1347826
C	-1.8223017	-4.5088427	1.2414395
H	-1.5383335	-2.6232563	2.2357702
C	-1.9585148	-5.1803838	0.0079327
H	-2.0315337	-5.0035768	-2.1505737
H	-1.8321397	-5.0987035	2.1698692
N	-2.0991860	-6.6301248	-0.0170333
O	-2.1386210	-7.2325407	1.0690271
O	-2.1679303	-7.1908364	-1.1245963
C	-3.6263377	-0.8476905	1.3905788
H	-4.4889927	-0.1348860	1.4161578
H	-4.0286275	-1.8768166	1.6013456
H	-2.9501094	-0.5720154	2.2271163
C	-3.8037287	-0.9558617	-1.0196186
H	-4.1820681	-2.0108879	-1.1104449
H	-4.7005475	-0.2899574	-0.9464794
H	-3.2797547	-0.7077259	-1.9667343

16-O
Energy = -2273.518640421
Thermal correction to Gibbs free Energy = 1150.9 KJ/mol

Pd	-0.7834936	-0.1113693	1.2790602
O	0.4993133	-0.9308192	-1.2541486
P	-1.9027967	0.8940486	-0.4269266
P	1.2682341	-1.0760680	1.5733196
C	1.3868974	-2.9115033	1.3242306
C	2.3461428	-3.5181960	0.4855725
H	3.0721268	-2.8921161	-0.0594072
C	2.3785334	-4.9162570	0.3368999
H	3.1305067	-5.3781522	-0.3249957
C	1.4583819	-5.7230149	1.0277077
H	1.4842875	-6.8191218	0.9086554
C	0.4981290	-5.1258575	1.8643688
H	-0.2328950	-5.7517931	2.4030968
C	0.4568536	-3.7294148	2.0040064
H	-0.3131240	-3.2596922	2.6404776
C	2.3546931	-0.7948629	3.0558442
C	2.1273203	0.3787379	3.8091258
H	1.3270126	1.0720323	3.4941961
C	2.8971803	0.6563189	4.9502842
H	2.7097776	1.5769317	5.5279792
C	3.8926754	-0.2465745	5.3647441
H	4.4896691	-0.0367383	6.2680412
C	4.1173415	-1.4232115	4.6290655
H	4.8922531	-2.1383632	4.9528764
C	3.3559486	-1.6958663	3.4789649
H	3.5390117	-2.6216425	2.9090460
C	-3.7589582	1.0367019	-0.4850878
C	-4.4509126	2.2268346	-0.7966034
H	-3.8893207	3.1354421	-1.0678669
C	-5.8571605	2.2636685	-0.7582504
H	-6.3840750	3.2025096	-0.9987279
C	-6.5877580	1.1132333	-0.4183701
H	-7.6896536	1.1445685	-0.3886730

C	-5.9052803	-0.0775084	-0.1059325
H	-6.4705331	-0.9835144	0.1700085
C	-4.5028867	-0.1119853	-0.1276634
H	-3.9699064	-1.0401461	0.1444457
C	-1.3507219	2.6442329	-0.7255545
C	-1.1476862	3.4576205	0.4128371
H	-1.2959842	3.0230582	1.4168150
C	-0.7457767	4.7954952	0.2737341
H	-0.5940042	5.4172495	1.1718199
C	-0.5205810	5.3353139	-1.0055235
H	-0.1926779	6.3825734	-1.1150727
C	-0.7054940	4.5307624	-2.1423120
H	-0.5240226	4.9446915	-3.1484810
C	-1.1216985	3.1942149	-2.0051146
H	-1.2642298	2.5754306	-2.9059710
C	-0.3813950	-0.7012040	-2.2933213
C	-0.1546237	-1.3523027	-3.5206743
H	0.7537017	-1.9652244	-3.6314759
C	-1.0848940	-1.2387730	-4.5638206
H	-0.9010332	-1.7538951	-5.5211005
C	-2.2545677	-0.4849949	-4.3719416
H	-2.9996488	-0.3961813	-5.1795587
C	-2.4841079	0.1472101	-3.1396039
H	-3.4164119	0.7168905	-2.9936929
C	-1.5582491	0.0647324	-2.0732486
C	1.7090361	-0.2922413	-1.1092251
C	2.3503982	0.3922733	-2.1549017
H	1.8646007	0.4677212	-3.1398251
C	3.6085842	0.9777791	-1.9308093
H	4.1091046	1.5131610	-2.7546967
C	4.2216137	0.8807978	-0.6721905
H	5.2106537	1.3341335	-0.4964936
C	3.5607960	0.2137265	0.3747182
H	4.0315971	0.1636382	1.3697318
C	2.2941868	-0.3751290	0.18558

14-N

Energy = -3054.845831766

Thermal correction to Gibbs free Energy = 1,785.86 KJ/mol

Pd	-0.5046362	0.3759136	1.0166302
C	-0.9033074	2.3051408	1.5205824
N	-0.6656357	-0.0219392	3.0612542
N	2.2701537	-0.6095055	-0.6020019
P	-0.0851485	-2.1177778	0.8263159
P	-0.4339967	0.8778940	-1.2841962
C	0.1977841	2.4777704	-1.9971912
C	1.0644574	2.5065609	-3.1105818
H	1.4336049	1.5658016	-3.5460079
C	1.4497806	3.7296504	-3.6872471
H	2.1261123	3.7307417	-4.5585302
C	0.9736156	4.9408643	-3.1610698
H	1.2764873	5.9001677	-3.6126634
C	0.1012640	4.9214283	-2.0591688
H	-0.2872066	5.8646701	-1.6412303
C	-0.2861854	3.7019871	-1.4816156
H	-0.9851152	3.7074644	-0.6324380
C	-2.1758015	0.9301983	-1.9496714
C	-3.2226605	0.4355114	-1.1415711
H	-2.9786426	0.0311274	-0.1423337
C	-4.5571865	0.4887912	-1.5799902
H	-5.3616608	0.1037349	-0.9317052
C	-4.8635641	1.0466151	-2.8324331
H	-5.9101569	1.0960575	-3.1761836
C	-3.8317765	1.5624896	-3.6365040
H	-4.0673301	2.0202106	-4.6117614
C	-2.4984876	1.5132552	-3.1963607
H	-1.7051985	1.9520411	-3.8232499
C	-0.7699662	-3.0106550	2.2965208
C	-1.9164454	-3.8267891	2.1984714
H	-2.3633165	-4.0385899	1.2144550
C	-2.5013493	-4.3840827	3.3506054
H	-3.3942127	-5.0236389	3.2524691
C	-1.9464567	-4.1297706	4.6154715
H	-2.4033218	-4.5662604	5.5195697
C	-0.7981420	-3.3258946	4.7228589
H	-0.3488103	-3.1327535	5.7112886
C	-0.2159987	-2.7582115	3.5761534
H	0.6948142	-2.1533003	3.6749912
C	-0.6752555	-3.2221092	-0.5569853
C	-1.9202890	-2.9471263	-1.1616467
H	-2.4842754	-2.0472707	-0.8705212
C	-2.4564493	-3.8116817	-2.1316190
H	-3.4323623	-3.5781013	-2.5886115
C	-1.7483900	-4.9609579	-2.5187346
H	-2.1666874	-5.6411300	-3.2794108
C	-0.5006396	-5.2367425	-1.9329970
H	0.0642239	-6.1358165	-2.2319438
C	0.0324159	-4.3751453	-0.9601935
H	1.0084385	-4.6096338	-0.5056770
C	3.0487804	0.5915356	-0.5871840
C	3.6911270	1.0772819	-1.7456501
H	3.5835525	0.5334407	-2.6967545
C	4.4822490	2.2370275	-1.6810543
H	4.9759599	2.6064209	-2.5951862
C	4.6584058	2.9142178	-0.4640096
H	5.2825689	3.8211856	-0.4163379
C	4.0301879	2.4225336	0.6959983
H	4.1596491	2.9453324	1.6580932
C	3.2251747	1.2771392	0.6367210
H	2.7225991	0.8914897	1.5381448
C	2.6867718	-1.6551900	0.2844464
C	4.0680910	-1.8778824	0.4998549
H	4.7952518	-1.2089627	0.0140631
C	4.5131676	-2.9375530	1.2991912
H	5.5946969	-3.0936678	1.4467071
C	3.5803909	-3.8086201	1.8875464
H	3.9167084	-4.6574804	2.5048656
C	2.2090491	-3.5815871	1.6987491
H	1.4830554	-4.2483882	2.1894472
C	1.7337267	-2.4968453	0.9251504
C	1.6399497	-1.0199551	-1.8279422
C	2.2245692	-2.0730626	-2.5662318

H	3.1434631	-2.5392131	-2.1776136
C	1.6601759	-2.5214770	-3.7672701
H	2.1378809	-3.3434025	-4.3252551
C	0.4897763	-1.9159155	-4.2507822
H	0.0276203	-2.2606136	-5.1899745
C	-0.1045744	-0.8740454	-3.5223113
H	-1.0394619	-0.4310283	-3.8976859
C	0.4455044	-0.4056220	-2.3084367
C	0.1372369	3.2103898	1.8468737
C	-2.2357326	2.7838362	1.5946014
C	-0.1296792	4.5302830	2.2380583
H	1.1882141	2.8860578	1.7877959
C	-2.5248903	4.1032716	1.9768876
H	-3.0788150	2.1199521	1.3432820
C	-1.4637190	4.9658587	2.2958484
H	0.6747217	5.2351336	2.4941938
H	-3.5568792	4.4795984	2.0335270
C	0.1678269	0.7142999	3.9878837
H	-0.1649962	1.7662548	4.2042527
H	0.1828512	0.1810615	4.9763914
H	1.2184542	0.7613399	3.6236729
C	-2.0375158	-0.0715014	3.5204000
H	-2.0813287	-0.5291799	4.5442480
H	-2.5408756	0.9322900	3.5983237
H	-2.6533247	-0.7127790	2.8489365
N	-1.7559584	6.3568535	2.6972361
O	-2.9398418	6.7117715	2.7247823
O	-0.7989030	7.0869120	2.9799151

15-N

Energy = -3054.835634246

Thermal correction to Gibbs free Energy = 1782.28 KJ/mol

Imaginary frequency = -277.82 cm^{-1}

Pd	-0.5335190	0.3288891	0.9413910
C	-0.9809533	2.1579437	1.7556187
N	-0.9217787	0.5057183	2.9978124
N	2.2782597	-0.6984854	-0.6310213
P	-0.0970944	-2.1414872	0.8001609
P	-0.3842462	0.8492240	-1.3278188
C	0.3320529	2.4399944	-1.9619223
C	1.1250412	2.4964992	-3.1282916
H	1.3997454	1.5686717	-3.6546824
C	1.5595889	3.7338797	-3.6338609
H	2.1762711	3.7626448	-4.5479534
C	1.2095611	4.9269675	-2.9799281
H	1.5518173	5.8973056	-3.3766599
C	0.4186025	4.8779711	-1.8193051
H	0.1363609	5.8070978	-1.2977375
C	-0.0200599	3.6441036	-1.3121960
H	-0.6481561	3.6222984	-0.4094744
C	-2.0977858	0.9721488	-2.0529484
C	-3.1855630	0.4689124	-1.3070550
H	-2.9894929	0.0206028	-0.3162851
C	-4.5004331	0.5694999	-1.7931452
H	-5.3380025	0.1779963	-1.1922427
C	-4.7461037	1.1844039	-3.0322630
H	-5.7773476	1.2722034	-3.4126988
C	-3.6730239	1.7084655	-3.7742818
H	-3.8602188	2.2110628	-4.7377796
C	-2.3591473	1.6109412	-3.2861121
H	-1.5320274	2.0541674	-3.8641258
C	-0.7967705	-2.9504041	2.3173917
C	-1.8906432	-3.8406049	2.2668444
H	-2.3054146	-4.1538557	1.2958008
C	-2.4595814	-4.3424756	3.4513547
H	-3.3121455	-5.0393912	3.3909600
C	-1.9419362	-3.9665354	4.7014453
H	-2.3865726	-4.3632408	5.6292668
C	-0.8476444	-3.0849023	4.7611118
H	-0.4279634	-2.7886104	5.7371068
C	-0.2857005	-2.5731126	3.5813701
H	0.5742826	-1.8889821	3.6446141
C	-0.7115124	-3.2882407	-0.5318374
C	-1.9566853	-3.0173877	-1.1390192
H	-2.5115653	-2.1049188	-0.8694575
C	-2.5011630	-3.9005485	-2.0870435
H	-3.4760634	-3.6705019	-2.5478317
C	-1.8016590	-5.0633604	-2.4497262
H	-2.2264509	-5.7569989	-3.1943835
C	-0.5541978	-5.3347492	-1.8614507
H	0.0038770	-6.2437926	-2.1424361
C	-0.0120610	-4.4549066	-0.9100995
H	0.9638351	-4.6852314	-0.4531057
C	3.0609844	0.5009886	-0.6282177
C	3.7186137	0.9600888	-1.7886962
H	3.6173139	0.3987333	-2.7303698
C	4.5102004	2.1198432	-1.7382680
H	5.0162527	2.4696766	-2.6532670
C	4.6682401	2.8244142	-0.5342137
H	5.2906873	3.7330649	-0.4982828
C	4.0208057	2.3616130	0.6268586
H	4.1319692	2.9097535	1.5768988
C	3.2176543	1.2145141	0.5815584
H	2.6986076	0.8513148	1.4827183
C	2.6777858	-1.7309557	0.2763593
C	4.0546010	-1.9592166	0.5140358
H	4.7927297	-1.3102108	0.0179108
C	4.4812190	-2.9976942	1.3505931
H	5.5596990	-3.1585490	1.5142172
C	3.5351897	-3.8407654	1.9590756
H	3.8584263	-4.6701911	2.6089427
C	2.1678640	-3.6097541	1.7471012
H	1.4306045	-4.2523086	2.2538030
C	1.7116127	-2.5494340	0.9296420
C	1.6375978	-1.1087539	-1.8495829
C	2.1862235	-2.1936956	-2.5689510
H	3.0859652	-2.6874073	-2.1694093
C	1.6117514	-2.6367495	-3.7674298
H	2.0610049	-3.4839022	-4.3111877
C	0.4698111	-1.9922952	-4.2679155
H	0.0020573	-2.3304136	-5.2067449

C	-0.0898430	-0.9191144	-3.5566084
H	-1.0049591	-0.4454728	-3.9436563
C	0.4687179	-0.4567669	-2.3445746
C	0.0602508	3.0786993	2.0921225
C	-2.3059463	2.6856715	1.6470317
C	-0.2030647	4.4296695	2.3213769
H	1.1032257	2.7315512	2.1488677
C	-2.5748357	4.0378304	1.8693382
H	-3.1403867	2.0268002	1.3603301
C	-1.5230079	4.9143948	2.2085126
H	0.6007064	5.1378846	2.5704424
H	-3.5920703	4.4446198	1.7717840
C	0.0515972	0.8467289	4.0097977
H	-0.0974018	1.8736595	4.4433182
H	-0.0043068	0.1200002	4.8595256
H	1.0841840	0.8026462	3.6038537
C	-2.2623791	0.3487672	3.5168048
H	-2.2641979	-0.3817083	4.3640071
H	-2.7037463	1.3112571	3.8952415
H	-2.9439782	-0.0535233	2.7381338
N	-1.7981590	6.3281567	2.4288611
O	-2.9741203	6.7167643	2.3324592
O	-0.8403625	7.0728880	2.6997823

16-N
Energy = -2484.543076205
Thermal correction to Gibbs free Energy = 1376.89 KJ/mol

Pd	-0.9349181	-0.2524007	1.2250914
N	0.1012967	-0.8588363	-1.5491257
P	-2.0275318	1.1625073	-0.1597782
P	1.2625955	-0.8358320	1.4343085
C	1.9854091	-2.5409380	1.5928472
C	3.2908559	-2.8687373	1.1643370
H	3.9175852	-2.1051195	0.6734849
C	3.7982991	-4.1643084	1.3561496
H	4.8194895	-4.4089036	1.0178424
C	3.0074065	-5.1476405	1.9779642
H	3.4063588	-6.1650254	2.1273797
C	1.7059781	-4.8311865	2.4027668
H	1.0781598	-5.5995795	2.8839456
C	1.1967186	-3.5357246	2.2070226
H	0.1702090	-3.2865369	2.5266559
C	2.1678312	0.0932472	2.7698562
C	1.8418497	1.4595911	2.9457673
H	1.1069866	1.9317949	2.2706713
C	2.4359868	2.2101314	3.9705529
H	2.1749693	3.2753247	4.0872748
C	3.3491274	1.6051047	4.8549376
H	3.8072933	2.1923364	5.6683179
C	3.6659704	0.2465022	4.6987559
H	4.3753160	-0.2398859	5.3894761
C	3.0844564	-0.5052277	3.6604378
H	3.3473942	-1.5689980	3.5500814
C	-3.8509911	1.5433769	-0.0361115
C	-4.4038244	2.8365938	-0.1615803
H	-3.7494442	3.6967291	-0.3777203
C	-5.7872426	3.0384791	-0.0062067
H	-6.2038990	4.0554849	-0.1013112
C	-6.6359844	1.9527659	0.2669690
H	-7.7200491	2.1138455	0.3901039
C	-6.0939921	0.6605973	0.3940054
H	-6.7516488	-0.1959991	0.6178906
C	-4.7119050	0.4608539	0.2551787
H	-4.2844986	-0.5489400	0.3859254
C	-1.2777938	2.8613928	-0.1247610
C	-1.2261989	3.5279935	1.1227439
H	-1.6272704	3.0290643	2.0220531
C	-0.6680849	4.8113591	1.2250763
H	-0.6455442	5.3213830	2.2028885
C	-0.1284358	5.4405547	0.0872999
H	0.3206703	6.4443485	0.1690085
C	-0.1602946	4.7796037	-1.1509342
H	0.2654844	5.2622956	-2.0466673
C	-0.7366429	3.5003459	-1.2597533
H	-0.7612973	2.9962053	-2.2389446
C	0.1262650	-2.2833043	-1.4502066
C	1.3055920	-3.0262551	-1.6777085
H	2.2434026	-2.5010752	-1.9152596
C	1.2817411	-4.4290459	-1.6098000
H	2.2124363	-4.9924008	-1.7882588
C	0.0875377	-5.1126670	-1.3287721
H	0.0736740	-6.2136705	-1.2805672
C	-1.0904614	-4.3749171	-1.1065738
H	-2.0350289	-4.8960608	-0.8770685
C	-1.0740369	-2.9747316	-1.1586325
H	-1.9910952	-2.3952535	-0.9690163
C	-0.9287023	-0.3160338	-2.3997281
C	-0.9948910	-0.7909706	-3.7313125
H	-0.2712445	-1.5597092	-4.0464920
C	-1.9443766	-0.3028858	-4.6370054
H	-1.9667591	-0.6859787	-5.6707261
C	-2.8563764	0.6802481	-4.2178937
H	-3.6082785	1.0825119	-4.9167120
C	-2.8214088	1.1353304	-2.8921715
H	-3.5671101	1.8773739	-2.5647544
C	-1.8798606	0.6476664	-1.9536212
C	1.3109562	-0.1180111	-1.3658665
C	1.8228188	0.6408982	-2.4432327
H	1.2635027	0.6588365	-3.3913081
C	3.0301985	1.3467149	-2.3345638
H	3.4029424	1.9253771	-3.1961593
C	3.7632658	1.2935494	-1.1399392
H	4.7200963	1.8319718	-1.0419386
C	3.2537270	0.5644056	-0.0525016
H	3.8091660	0.5690826	0.8990219
C	2.0261019	-0.1309884	-0.1234054

17
Energy = -1620.247383279
Thermal correction to Gibbs free Energy = 859.52 KJ/mol

Pd	-0.1143069	0.3931598	0.0056504
P	-0.4305314	2.7894048	0.0244612
P	2.2012828	0.2487955	0.1998626
C	0.9194234	4.0698913	0.1967231
H	1.6660986	3.9533013	-0.6184077
H	0.5059323	5.1021003	0.1489063
H	1.4435218	3.9476712	1.1694561
C	-1.5916310	3.3292905	1.3603132
H	-1.8435203	4.4088052	1.2644219
H	-2.5108253	2.7163098	1.2669747
H	-1.1326034	3.1477820	2.3560253
C	2.9462098	1.0322150	1.7096037
H	4.0526922	0.9123756	1.7269271
H	2.6965979	2.1134477	1.7392359
H	2.5150453	0.5539848	2.6154134
C	2.9610893	-1.4383970	0.2602178
H	2.6869282	-2.0024442	-0.6559526
H	4.0687983	-1.3747338	0.3443408
H	2.5518012	-1.9980553	1.1275635
C	3.1620022	1.0298388	-1.1840217
H	4.2592947	0.9222922	-1.0314836
H	2.8787444	0.5401328	-2.1405399
H	2.9084725	2.1078542	-1.2618318
C	-1.2754507	3.3783318	-1.5130392
H	-2.1863548	2.7597821	-1.6458336
H	-1.5557611	4.4524410	-1.4362448
H	-0.6082791	3.2349380	-2.3900787
N	-2.2134812	0.5280692	-0.1915267
C	-0.2464765	-1.6440024	-0.0464696
C	-0.4115651	-2.4010444	1.1422099
C	-0.1858825	-2.3604944	-1.2696059
C	-0.5102441	-3.8009853	1.1218549
H	-0.4717252	-1.8878421	2.1178493
C	-0.2810259	-3.7601545	-1.3141101
H	-0.0667535	-1.8154292	-2.2220348
C	-0.4394644	-4.4683541	-0.1116779
H	-0.6414909	-4.3899598	2.0417419
H	-0.2360484	-4.3184529	-2.2610529
N	-0.5307878	-5.9422932	-0.1447939
O	-0.4605490	-6.4984605	-1.2467290
O	-0.6690839	-6.5345099	0.9315004
C	-2.7197243	-0.0058548	-1.4419373
H	-2.6808171	-1.1277431	-1.5297125
H	-3.7985908	0.2810492	-1.5705455
H	-2.1614673	0.4148300	-2.3100972
C	-2.9437142	-0.0387312	0.9261392
H	-4.0287833	0.2454321	0.8566021
H	-2.9167562	-1.1623917	0.9890896
H	-2.5602395	0.3568078	1.8946427

18

Energy = -1620.230595259

Thermal correction to Gibbs free Energy = 846.21 KJ/mol

Imaginary frequency = -278.26 cm^{-1}

Pd	-0.0972604	0.4794178	-0.0004519
C	-0.3649465	-1.5493171	0.0050713
N	-2.0168193	-0.3692618	-0.0010146
P	-0.7130509	2.8372470	-0.0102418
P	2.2082274	0.5049571	0.0030205
C	0.4471628	4.2957958	0.0219094
H	1.1209061	4.2580290	-0.8614863
H	-0.1021215	5.2643146	0.0097342
H	1.0755572	4.2545076	0.9379626
C	-1.8445621	3.2523906	1.4030027
H	-2.2621818	4.2806112	1.3151664
H	-2.6767883	2.5172395	1.4246516
H	-1.2872804	3.1667459	2.3609289
C	3.0173280	1.3222382	1.4599624
H	4.1271157	1.2674654	1.3954641
H	2.7077409	2.3875317	1.5147480
H	2.6828225	0.8171043	2.3914924
C	3.0172442	-1.1567036	-0.0091932
H	2.6835406	-1.7185120	-0.9077977
H	4.1266506	-1.0736870	-0.0057255
H	2.6788581	-1.7335686	0.8780820
C	3.0274036	1.3472414	-1.4341257
H	4.1366575	1.2866633	-1.3654491
H	2.6955362	0.8618975	-2.3770555
H	2.7227427	2.4147511	-1.4696405
C	-1.7711368	3.2569511	-1.4781172
H	-2.6025115	2.5233482	-1.5433167
H	-2.1913119	4.2855707	-1.4094310
H	-1.1665124	3.1722414	-2.4069981
C	-0.2480483	-2.2860612	1.2298985
C	-0.2461461	-2.2953600	-1.2138775
C	-0.0510131	-3.6670984	1.2361939
H	-0.3000300	-1.7573909	2.1960538
C	-0.0494804	-3.6764133	-1.2093409
H	-0.2962915	-1.7739729	-2.1840477
C	0.0486039	-4.3705123	0.0161561
H	0.0453719	-4.2288565	2.1773428
H	0.0481617	-4.2453282	-2.1460484
N	0.2736432	-5.8059551	0.0216939
O	0.3650090	-6.3883732	-1.0735660
O	0.3653327	-6.3797067	1.1216161
C	-2.7536878	-0.6475364	-1.2106103
H	-2.9898567	-1.7398782	-1.3356266
H	-3.7279248	-0.0929333	-1.2167390
H	-2.1855714	-0.3246142	-2.1080564
C	-2.7577664	-0.6409858	1.2076015
H	-3.7338177	-0.0896070	1.2056462
H	-2.9910015	-1.7331444	1.3395835
H	-2.1944972	-0.3094822	2.1049748

19

Energy = -1049.943172202

Thermal correction to Gibbs free Energy = 451.61 KJ/mol

Pd	0.0144173	-0.0013053	0.0100029
P	-0.8390558	1.5933830	1.4076410
P	0.8503088	-1.5941980	-1.4005482

C	0.3739801	2.5696164	2.4279338
H	1.0904866	3.0829008	1.7514070
H	-0.1344751	3.3268056	3.0682535
H	0.9540640	1.8740612	3.0715902
C	-2.0276487	1.0193197	2.7204555
H	-2.3942751	1.8624874	3.3500686
H	-2.8931153	0.5192751	2.2354207
H	-1.5221572	0.2707648	3.3675820
C	2.6668233	-1.5067709	-1.7979483
H	2.9824384	-2.3210642	-2.4898523
H	2.8918637	-0.5225811	-2.2621223
H	3.2505164	-1.5779498	-0.8553056
C	0.6903117	-3.3676877	-0.8573717
H	-0.3835784	-3.6071610	-0.7018213
H	1.1207632	-4.0743198	-1.6037652
H	1.2109549	-3.4988643	0.1154673
C	0.1145094	-1.6768208	-3.1084648
H	0.5785723	-2.4809126	-3.7248199
H	-0.9779512	-1.8617419	-3.0257423
H	0.2555884	-0.6982447	-3.6154457
C	-1.8192968	2.9625225	0.6137908
H	-2.6762793	2.5229633	0.0598902
H	-2.2009161	3.6920643	1.3645946
H	-1.1768497	3.4934581	-0.1208906

17-B

Energy = -1646.837275506

Thermal correction to Gibbs free Energy = 927.75 KJ/mol

Pd	-0.2676735	0.1881640	0.2279768
P	-1.4611129	1.5815191	1.8471712
P	1.8146142	1.2790786	0.0211245
C	-1.6638005	0.7139056	3.4717710
H	-0.6721466	0.5548179	3.9478453
H	-2.3063003	1.3045506	4.1617844
H	-2.1352478	-0.2718647	3.2821686
C	-3.2032417	1.9074006	1.3361397
H	-3.7679181	2.4125906	2.1503341
H	-3.2080028	2.5404543	0.4245671
H	-3.6657381	0.9279533	1.0987552
C	1.9760724	3.1227895	0.0598953
H	3.0483178	3.4159824	0.0174550
H	1.4379612	3.5536584	-0.8095215
H	1.5228781	3.5288818	0.9871640
C	2.8830669	0.7561409	1.4461904
H	2.9664887	-0.3514466	1.4568016
H	3.9029946	1.1937472	1.3663153
H	2.4215688	1.0809818	2.4031363
C	2.8580648	0.8406875	-1.4388036
H	3.8295258	1.3802818	-1.3975621
H	3.0365018	-0.2540297	-1.4552148
H	2.3168348	1.1170289	-2.3676251
C	-0.9071088	3.2699415	2.4053492
H	-0.8244238	3.9413210	1.5244701
H	-1.6312235	3.7119704	3.1258118
H	0.0864583	3.2058518	2.9002669
N	-1.9663903	-1.0170560	0.6366710
C	0.4325220	-1.2771210	-1.0304875
C	1.2551086	-2.3060137	-0.5047212
C	0.1079016	-1.3321474	-2.4064757
C	1.7465528	-3.3439965	-1.3124612
H	1.5212460	-2.3168045	0.5671512
C	0.5837589	-2.3646211	-3.2298875
H	-0.5330802	-0.5545280	-2.8517692
C	1.4048925	-3.3582875	-2.6736289
H	2.3858964	-4.1440947	-0.9112732
H	0.3324008	-2.4156581	-4.2996369
N	1.9209566	-4.4431480	-3.5365591
O	2.6494761	-5.2940943	-3.0142458
O	1.5956660	-4.4329602	-4.7279722
B	-0.8769581	1.7908105	-1.4040510
C	-2.8232151	-1.3489523	-0.4856256
H	-3.7669214	-1.8221720	-0.1054280
H	-3.1118817	-0.4346009	-1.0475286
H	-2.3757220	-2.0750861	-1.2194271
C	-1.5773501	-2.2254391	1.3425582
H	-2.4850632	-2.7044731	1.7981210
H	-1.0908694	-3.0134755	0.7041929
H	-0.8782053	-1.9962903	2.1808247
H	-1.9129964	1.2008316	-1.6830149
H	-0.9681972	2.8360667	-0.7611315
H	0.0430626	1.7409530	-2.2179603

18-B

Energy = -1646.822548181

Thermal correction to Gibbs free Energy = 924.86 KJ/mol

Imaginary frequency = -273.44 cm^{-1}

Pd	-0.1129674	0.4397307	-0.0372274
C	-0.4317814	-1.5929847	-0.0545928
N	-2.0812430	-0.3274002	0.0293048
P	-0.6764873	2.8495611	0.2633879
P	2.1957980	0.3293265	0.1235687
C	-1.4295676	3.2224295	1.9224596
H	-0.6709688	3.0790376	2.7223876
H	-1.8180127	4.2641800	1.9761119
H	-2.2656908	2.5156537	2.1111935
C	-2.0384230	3.3268234	-0.8945440
H	-2.4053940	4.3591433	-0.7005868
H	-1.6656544	3.2510429	-1.9379217
H	-2.8733450	2.6036794	-0.7787196
C	3.3035879	1.6182105	-0.6048148
H	4.3651328	1.4145643	-0.3418453
H	3.1867102	1.6188446	-1.7082333
H	3.0166466	2.6210678	-0.2252381
C	2.6599299	0.3608028	1.9195663
H	2.1323558	-0.4636237	2.4451517
H	3.7569766	0.2311617	2.0544845
H	2.3490810	1.3255090	2.3751052
C	2.9513184	-1.2481231	-0.4607335
H	4.0454917	-1.2601694	-0.2612677
H	2.4650179	-2.1009855	0.0580442

H	2.7674971	-1.3573344	-1.5499279
C	0.5115520	4.2650058	0.0671019
H	0.9887542	4.2021681	-0.9341006
H	-0.0016435	5.2481801	0.1634525
H	1.3066917	4.2005682	0.8412964
C	-0.2569800	-2.2834890	1.1855748
C	-0.4080365	-2.3671586	-1.2524946
C	-0.0680367	-3.6675737	1.2264547
H	-0.2667587	-1.7289167	2.1392284
C	-0.2285537	-3.7508878	-1.2132272
H	-0.5089306	-1.8701004	-2.2293369
C	-0.0541758	-4.4042776	0.0253679
H	0.0736409	-4.2013719	2.1778999
H	-0.2022794	-4.3505455	-2.1352166
N	0.1492551	-5.8481687	0.0652378
O	0.3064941	-6.3829213	1.1752799
O	0.1562412	-6.4651005	-1.0119665
B	0.3651826	0.9021923	-2.2853267
C	-2.9063809	-0.7044410	-1.0925543
H	-3.8906189	-0.1719922	-1.0411934
H	-2.4271215	-0.4165061	-2.0503895
H	-3.1207246	-1.8068654	-1.1291460
C	-2.7082213	-0.5381475	1.3128673
H	-3.7020765	-0.0219426	1.3534179
H	-2.8868101	-1.6242509	1.5442242
H	-2.0888887	-0.1248897	2.1392005
H	-0.8048141	0.7106599	-2.6041254
H	0.7595080	2.0681064	-2.2637467
H	1.1877234	0.0525187	-2.6188927

19-B

Energy = -1076.553701410

Thermal correction to Gibbs free Energy = 524.98 KJ/mol

Pd	0.1306370	-0.1030409	0.1975838
P	-0.7877366	1.4580427	1.6438949
P	1.0064954	-1.7379530	-1.1887836
C	-0.2424545	1.3147532	3.4165234
H	0.8620994	1.4230497	3.4749803
H	-0.7182574	2.0915182	4.0570273
H	-0.5107125	0.3092325	3.8063237
C	-2.6332013	1.4417176	1.8062535
H	-2.9906589	2.2302890	2.5059987
H	-3.0757174	1.6044417	0.8007657
H	-2.9620890	0.4454668	2.1716236
C	2.0375052	-1.1501812	-2.6089495
H	2.3806107	-1.9960712	-3.2456242
H	1.4311372	-0.4434356	-3.2136935
H	2.9183646	-0.5990488	-2.2161352
C	2.1425124	-2.9501422	-0.3514986
H	1.5954301	-3.4656809	0.4667254
H	2.5296570	-3.7112345	-1.0661306
H	3.0003990	-2.4050991	0.0978932
C	-0.2179200	-2.8608398	-2.0085752
H	0.2843202	-3.6097590	-2.6617043
H	-0.8118525	-3.3879120	-1.2313832
H	-0.9123573	-2.2409093	-2.6139498
C	-0.4284826	3.2335931	1.2644394
H	-0.7653375	3.4461697	0.2279660
H	-0.9403150	3.9178973	1.9775015
H	0.6685246	3.4016679	1.3102556
B	-0.6917548	0.9798216	-1.5510942
H	-1.7155577	1.5394099	-1.1398210
H	-0.8872033	0.1213879	-2.4196023
H	0.3039156	1.7028484	-1.7088105

[1] SMART, *Bruker ACX Inc.* **2001**, Madison, Wisconsin, USA.
[2] SAINT+, *Buker AXS Inc.,* **2009**, Madison, Wisconsin, USA.
[3] SADABS, *Bruker ACX Inc.* **2008**, Madison, Wisconsin, USA.
[4] a) X-Area Recipe, Stoe & Cie GmbH, Darmstadt, Germany, **2015** b) X-Area Pilatus3_SV, Stoe & Cie GmbH, Darmstadt, Germany, **2016**, c) X-Area Intergrate, Stoe & Cie GmbH, Darmstadt, Germany, **2016**; d) X-Area LANA, Stoe & Cie GmbH, Darmstadt, Germany, **2016**.
[5] O. V. Dolomanov, L. J. Bourhis, R. J. Gildea, J. A. K. Howard, H. Puschmann, *J. Appl. Cryst.* **2009**, *42*, 339-341.
[6] G. M. Sheldrick, *Acta Cryst.* **2008**, *A64*, 112-122.
[7] L. J. Farrugia, *J. Appl. Cryst.* **2012**, *45*, 849-854.
[8] a) F. Furche, R. Ahlrichs, C. Hattig, W. Klopper, M. Sierka, F. Weigend, *WIREs Comput. Mol. Sci.* **2014**, *4*, 91-100; b) F. Weigend, *Phys. Chem. Chem. Phys.* **2006**, *8*, 1057-1065.
[9] S. Grimme, J. Antony, S. Ehrlich, H. Krieg, *J. Chem. Phys.* **2010**, *132*, 154104-154123.
[10] a) J. P. Perdew, *Phys. Rev. B* **1986**, *33*, 8822-8824; b) F. Weigend, R. Ahlrichs, *Phys. Chem. Chem. Phys.* **2005**, *7*, 3297-3305.
[11] a) K. Eichkorn, O. Treutler, H. Öhm, M. Häser, R. Ahlrichs, *Chem. Phys. Lett.* **1995**, *242*, 652-660; b) K. Eichkorn, F. Weigend, O. Treutler, R. Ahlrichs, *Theor. Chem. Acc.* **1997**, *87*, 119-124.
[12] a) T. Schindler, M. Lux, M. Peters, L. T. Scharf, H. Osseili, L. Maron, M. E. Tauchert, *Organometallics* **2015**, *34*, 1978-1984; b) D. Schuhknecht, F. Ritter, M. E. Tauchert, *Chem. Commun.* **2016**, *52*, 11823-11826; c) P. Steinhoff, M. E. Tauchert, *Beilstein J. Org. Chem.* **2016**, *12*, 1573-1576.
[13] E. D. Glendening, J. K. Badenhoop, A. E. Reed, J. E. Carpenter, J. A. Bohmann, C. M. Morales, C. R. Landis, F. Weinhold, *NBO 6.0* **2013**, Theoretical Chemistry Institute, University of Wisconsin.
[14] M. J. Frisch, G. W. Trucks, H. B. Schlegel, G. E. Scuseria, M. A. Robb, J. R. Cheeseman, G. Scalmani, V. Barone, B. Mennucci, G. A. Petersson, H. Nakatsuji, M. Caricato, X. Li, H. P. Hratchian, A. F. Izmaylov, J. Bloino, G. Zheng, J. L. Sonnenberg, M. Hada, M. Ehara, K. Toyota, R. Fukuda, J. Hasegawa, M. Ishida, T. Nakajima, Y. Honda, O. Kitao, H. Nakai, T. Vreven, J. A. Montgomery, J. E. Peralta, F. Ogliaro, M. Bearpark, J. J. Heyd, E. Brothers, K. N. Kudin, V. N. Staroverov, R. Kobayashi, J. Normand, K. Raghavachari, A. Rendell, J. C. Burant, S. S. Iyengar, J. Tomasi, M. Cossi, N. Rega, J. M. Millam, M. Klene, J. E. Knox, J. B. Cross, V. Bakken, C. Adamo, J. Jaramillo, R. Gomperts, R. E. Stratmann, O. Yazyev, A. J. Austin, R. Cammi, C. Pomelli, J. W. Ochterski, R. L. Martin, K. Morokuma, V. G. Zakrzewski, G. A. Voth, P. Salvador, J. J. Dannenberg, S. Dapprich, A. D. Daniels, Farkas, J. B. Foresman, J. V. Ortiz, J. Cioslowski, D. J. Fox, *Gaussian 09, Revision D.01* **2013**, Gaussian Inc., Wallingford CT.
[15] a) G. A. Petersson, A. Bennett, T. G. Tensfeldt, M. A. Al-Laham, W. A. Shirley, *J. Chem. Phys.* **1988**, *89*, 2193-2218; b) G. A. Petersson, M. A. Al-Laham, *J. Chem. Phys.* **1991**, *94*, 6081-6090.
[16] A. Bergner, M. Dolg, W. Küchle, H. Stoll, H. Preuß, *Mol. Phys.* **1993**, *80*, 1431-1441.
[17] M. Dolg, U. Wedig, H. Stoll, H. Preuss, *J. Chem. Phys.* **1987**, *86*, 866-872.
